# Identifying research priorities for post-collision care in the United Kingdom: outcomes and methodological adaptations from the final prioritisation workshop

**DOI:** 10.1186/s13049-026-01628-y

**Published:** 2026-05-27

**Authors:** Tim Nutbeam, Laura Cottey, Kerry Dungay, Lauren R. Rodgers, Emily Foote, Caroline Leech, Claire E. Baker, Elizabeth Box, Louise Johnson, Brian Lee, Marilyn MacQueen, Rob Fenwick, Nigel Lang, Ianto Guy, Ian Marritt, Ian Dunbar, Ed B. G. Barnard, Mini -Review Authors, Nick Aveyard, Lily Eckersley-Jones, Cally Greiner-Cooper, Kershaun Mathew, Nigel Lang, Celia Lugt, Kirsten Raphael, Gregory Smith, Luke Tester, Jennifer Todd, Rosanna Watts, George Russam

**Affiliations:** 1 IMPACT, Centre for Post-Collision Research Innovation and Translation, Exeter, UK; 2 University of Plymouth, Plymouth, UK; 3 Academic Department of Military Emergency Medicine, Royal Centre for Defence Medicine, Birmingham, UK; 4 The Emergency Department, University Hospitals Plymouth NHSTrust, Plymouth, UK; 5 Devon Air Ambulance Trust, Exeter, UK; 6 University Hospitals Coventry & Warwickshire NHS Trust, Coventry, UK; 7 The Air Ambulance Service, Rugby, UK; 8 I-X and Dyson School of Design Engineering, Imperial College London, London, UK; 9 RAC Foundation, 89-91 Pall Mall, London, UK; 10 Leeds Teaching Hospitals NHS Trust, Leeds, UK; 11 Betsi Cadwaladr University Health Board, Wrexham Maelor Hospital, Croesnewedd Road, Wrexham, UK; 12 Wrexham University, Wrexham, UK; 13 Transport Research Laboratory, Birmingham, UK; 14 United Kingdom Rescue Organisation, World Rescue Organisation, Kingston upon Hull, UK; 15 Humberside Fire and Rescue, Kingston upon Hull, UK; 16 Federation Internationale de l’Automobile (FIA), Geneva, Switzerland; 17 Ian Dunbar Training and Consultancy, Hereford, UK; 18 IDEX Fire and Safety, Hereford, UK; 19 Emergency and Urgent Care Research in Cambridge (EUReCa), PACE Section, Department of Medicine, Cambridge University, Cambridge, UK

**Keywords:** Road traffic injury, Trauma, Post-collision care, Research priorities, Patient and public involvement, Emergency care, Rehabilitation

## Abstract

**Background:**

Road traffic injury remains a leading cause of death and serious injury in the United Kingdom, yet the post-collision phase of care has received comparatively little research attention. The Road Injury Chain of Survival framework identifies five interdependent links where coordinated action can improve outcomes. To address evidence gaps across this pathway, we conducted the first UK Priority Setting Partnership focused specifically on post-collision care, following James Lind Alliance methodology.

**Methods:**

A national open survey collected research uncertainties from patients, carers, bystanders, clinicians, emergency responders and policy stakeholders between July and August 2025. This was supplemented by a targeted literature review identifying research uncertainties from clinical guidelines and systematic reviews. All submissions underwent evidence checking using the BestBETs methodology. The Steering Group, comprising patients with lived experience, emergency service representatives, clinicians, and researchers, conducted interim prioritisation to produce a shortlist. A final prioritisation workshop was held in November 2025, using nominal group technique across three facilitated small-group rounds followed by plenary consensus. Methodological adaptations enabled structured remote participation for contributors unable to attend due to injury-related barriers.

**Results:**

In total, 179 survey submissions and 73 literature-derived questions were consolidated into 57 indicative uncertainties. Following evidence checking and interim prioritisation, 23 questions proceeded to the final workshop. Thirty-nine participants reached consensus on ten priorities. These emphasised preventable deaths and critical intervention windows, recognition of occult life-threatening injuries, multi-agency coordination, technology-assisted bystander care, automatic crash notification, first aid training effectiveness, emergency call-handler decision support, inequalities in injury patterns and care, treatment of entrapped casualties, and patient-centred recovery outcomes.

**Conclusion:**

This national Priority Setting Partnership provides an inclusive, stakeholder-driven foundation for research commissioning and policy development in post-collision care. The priorities highlight significant evidence gaps in the earliest phases of the care pathway and underscore the importance of addressing inequalities and aligning research with outcomes meaningful to survivors.

**Protocol:**

Nutbeam, T., Leech, C., Baker, CE. et al Identifying research priorities for post-collision care in the United Kingdom: protocol for a road injury priority setting partnership. *Scand J Trauma Resusc Emerg Med* 33, 203 (2025). https://doi.org/10.1186/s13049-025-01513-0.

**Supplementary Information:**

The online version contains supplementary material available at https://doi.org/10.1186/s13049-026-01628-y.

## Background

**Fig. 1:**
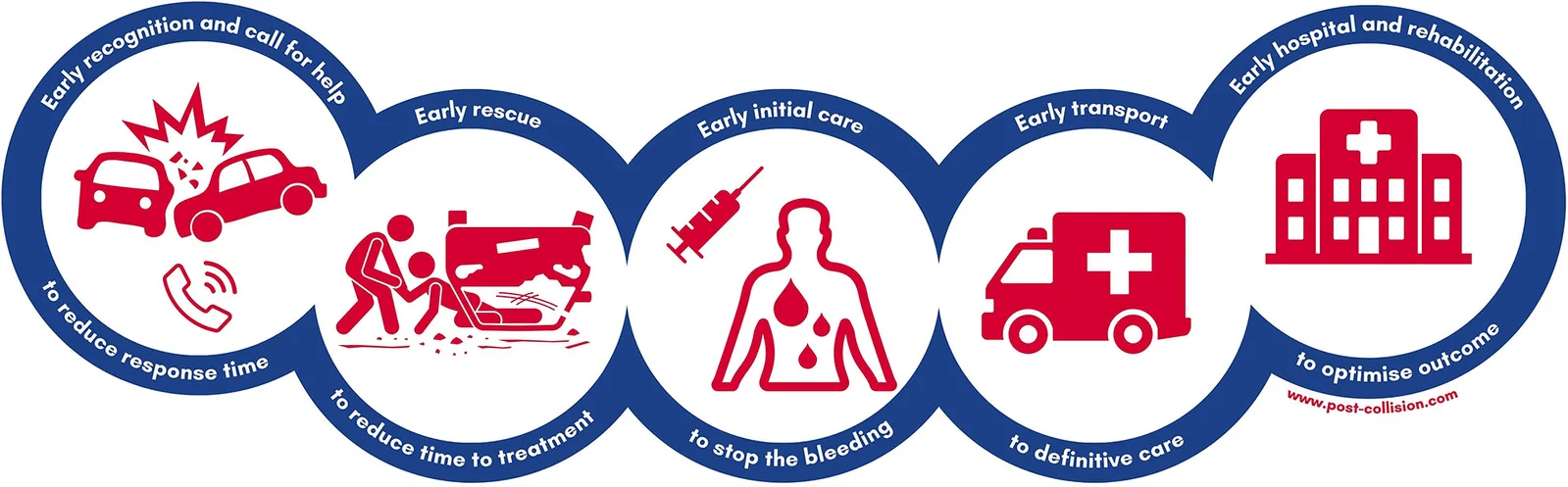
The Road Injury Chain of Survival

Road traffic injury remains a leading cause of death and serious injury worldwide and continues to impose a substantial human and economic burden in the United Kingdom [1, 2]. A recent UK road safety strategy has renewed emphasis on the post-collision response as a core component of the Safe System. The Safe System approach recognises that collisions will continue to occur and seeks to minimise death and serious injury through coordinated action across prevention, response, and recovery, highlighting the need not only to prevent collisions but also to improve outcomes once injury has occurred [3]. Despite sustained improvements in prevention and vehicle safety, the post-collision phase of care; spanning recognition and call for help, rescue, pre-hospital management, hospital treatment, and rehabilitation has received comparatively little research attention [4]. The Road Injury Chain of Survival framework highlights five interdependent links where timely, coordinated action can improve survival and recovery [5]. Strengthening each of these links requires high-quality evidence aligned with real-world needs (Fig. 1). Each link provides a distinct opportunity for research and evidence-driven intervention. Identifying the research priorities associated with the Road Injury Chain of Survival can underpin the coordinated action to improve survival and recovery.

To address this gap, the first UK Road Injury Priority Setting Partnership (PSP) focused specifically on post-collision care was established, based on the principles of the James Lind Alliance (JLA) methodology [6]. The PSP was designed to identify the most important unanswered research questions across the post-collision care pathway, as defined by patients, carers, bystanders, clinicians, emergency responders, researchers and policy stakeholders, including representatives from emergency services, healthcare, academia, third-sector organisations, industry, and the public. The intended end-users of the priorities are researchers, funders, policymakers, guideline developers, and organisations responsible for the planning and delivery of post-collision care, with the ultimate aim of improving outcomes for people injured on UK roads. The published protocol described a mixed-methods approach incorporating a national open survey, an evidence check of existing literature, and a final in-person prioritisation workshop to rank and agree the “Top 10” research priorities [7].

This paper reports the outcomes of the final prioritisation workshop and describes the methodological approach used to agree the final “Top 10” research priorities in post-collision care.

## Methods

Methods for this UK wide PSP followed the published protocol and is reported to REPRISE guidelines [7, 8]. The scope of the PSP was ‘post-collision care’, as previously defined, spanning from the point of collision through to early rehabilitation, with primary prevention excluded. Consistent with the Safe System framework, the PSP focused exclusively on the post-collision response pillar and did not consider the upstream pillars of safe roads, safe speeds, safe vehicles, or safe road users [9]. The PSP was overseen by a Steering Group comprising patients with lived experience of serious road traffic injury, family members, representatives from fire and ambulance services, clinicians, trauma psychologists, researchers, and policy partners. The Steering Group provided strategic direction throughout the process, including protocol development, oversight of scope, uncertainty verification, and preparation for the final prioritisation workshop.

During planning for the final workshop, it became apparent that some contributors with lived experience of severe road traffic injury were unable to attend in person due to ongoing physical limitations or logistical barriers. To uphold inclusivity and the principle that all stakeholder voices carry equal weight, the Steering Group approved methodological adaptations enabling structured remote participation. These adaptations included pre-workshop ranking of shortlisted questions and facilitated integration of remote participants’ rankings into the in-person consensus process. Bias was minimised through independent workshop facilitation, use of nominal group technique, mixed stakeholder group composition, equal weighting of all rankings and a documented decision trail at each stage. All Steering Group members were asked to declare potential conflicts of interest at the outset; none were identified that required exclusion or modification of roles.

### Collection of research uncertainties: 

Research uncertainties were submitted through a national open survey distributed across patients, carers, bystanders, clinicians, rescuers and other professionals. The survey opened on 4 July 2025 and closed on 31 August 2025, and was publicised on the IMPACT website (www.post-collision.com), shared through IMPACT and partner organisation social media channels, and circulated via the Centre’s bi-monthly newsletter to broaden reach. Responses were provided in free-text format and were not restricted to predefined categories.

In parallel, a targeted scan of clinical guidelines, systematic reviews, and recent high-level publications was undertaken to identify literature-derived research uncertainties relevant to post-collision care. This included national and international guidance and evidence syntheses that explicitly highlighted gaps in evidence or made recommendations for future research. Identified uncertainties were added to the dataset to ensure that recognised gaps in the literature were considered alongside stakeholder-generated questions.

### Data processing and organisation: 

All submissions were anonymised and reviewed for clarity. Similar or overlapping uncertainties were grouped, and where appropriate, merged into a single indicative question reflecting the core intent. This process was undertaken by a core group of the authorship team, with full transparency of decisions and opportunity for review by the Steering Group. Questions outside the agreed PSP scope were removed with recorded rationale.This stage produced a consolidated longlist of indicative uncertainties representing the breadth of public, professional and literature-derived submissions.

### Evidence checking: 

Each indicative uncertainty underwent an evidence check using BestBETs type methodology undertaken by resident doctors and prehospital professionals with supervision from Steering Group members [10]. Reviewers searched for recent systematic reviews, national and international clinical guidelines and relevant peer-reviewed studies using sources such as the Cochrane Library and PubMed. Searches focused on the most recent available evidence (typically within the previous ten years) and were limited to publications in English. The purpose of evidence checking was to determine whether a question remained an unanswered uncertainty, rather than to undertake an exhaustive review.

### Shortlisting: 

Questions the steering group deemed to be answered by current moderate- or high-certainty evidence were removed. Uncertainties with limited, conflicting or absent evidence remained within scope. All decisions and supporting evidence were documented in a decision trail (Supplementary materials - Sheet 1).

The verified longlist underwent interim prioritisation by the Steering Group. Each member independently assigned a single composite score to every question on a 1–5 scale, informed by their overall judgement of perceived priority, relevance, and breadth of potential impact. Individual scores were then summed to produce an aggregate score for each question. Questions were ordered primarily by median score, with the interquartile range (IQR) used to resolve ties where necessary. A predefined retention threshold was applied, with questions achieving a median score of 3.5 or higher progressing to the next stage. This process resulted in a shortlist of 23 questions, consistent with the practical number of items manageable within a one-day final prioritisation workshop (approximately 20–25) [6]. Where further discrimination between questions would have been required, the median score from patient and public involvement (PPIE) members was prespecified as an additional tie-breaker, although this was not needed in practice. The 23 questions were subsequently rewritten into plain English to support accessibility for all participants. This process was undertaken with review and input from PPIE Steering Group members to ensure clarity, relevance, and accessibility, and a lay summary was produced (supplementary materials).

### Pre-workshop ranking: 

Prior to the final prioritisation workshop, participants were asked to review the shortlist, reflect on their own priorities beforehand and complete a pre-workshop ranking. The same pre-workshop ranking exercise was also completed by participants who were unable to attend in person because of injury-related or logistical barriers. These individuals were asked to review and rank the 23 shortlisted questions in advance, and were offered additional support where required. Their rankings were returned prior to the workshop and were noted during the small group discussions.

## Final stage prioritisation: workshop process and outcomes

### Workshop format: 

An independent Chair led the final prioritisation workshop on the 12th November 2025 at the Royal College of Surgeons of Edinburgh. 39 participants attended including patients, carers, bystanders, clinicians, emergency services personnel, researchers, and stakeholders (Table 1).

**Table 1:** Stakeholder, professionals and public attendance at workshop

Stakeholder Group	Number
Professionals	33
Members of the public	6
Total	39
**Professionals breakdown**:	
Police/Fire and Rescue	4
Ambulance and Emergency Healthcare Professionals (including Technicians, Paramedics, Doctors, Advanced Care Practitioner, Nurses)	7
Medical College	5
Third Sector (included organisations involved with support and advocacy, government, health, research and work-related road safety)	9
Private (included consultancies who specialise in data and research in post-collision investigation and car safety, legal sector and rescue services)	4
University	1
Public Breakdown: *some members fall into more than one category	
Survivor of road injury	3
Bereaved family member	3
Family member-bystanders*	3

*Participants in this category were family members of a road-injured person who were also present at the collision as a Bystander

Three patient representatives were unable to attend in person for health or logistical reasons; therefore their contributions were incorporated as described above. The workshop consisted of three rounds of small-group discussions, one whole-group session, and a final consensus ranking. To ensure equitable participation and fair representation, each small group included at least two patient representatives at every stage, and facilitators were specifically briefed to ensure that patient perspectives were actively invited, protected and integrated throughout the discussions.

In order to support confidence and meaningful engagement, patient participants were offered a preparatory briefing call in advance of the workshop, during which the process, format and expectations were explained.

Three facilitators led small-group discussions of 11–13 participants using nominal group technique. Groups were purposively mixed to include representation from across stakeholder categories. The workshop was structured into three rounds: an initial discussion of participants’ pre-rankings, followed by two iterative ranking rounds with aggregation and feedback between rounds to inform subsequent discussion.

A final review of the combined ranking then took place with all participants, led by the independent Chair, to ensure all participants achieved consensus on a Top 10. At this point, two questions were felt to be suitable for combining and this took place in agreement with everyone. The final ordering was agreed when stability of statement positions was reached without unresolved objections across stakeholder groups.

### Integration of remote contributions

Rankings submitted by remote participants were incorporated directly into the small-group materials to ensure continuity with the JLA approach of equal weighting of voices. Their ranks were written on the question cards used in each group and were reviewed by facilitators when discussing relative importance, areas of convergence and areas of divergence. Facilitators were briefed to ensure that these views were explicitly acknowledged during discussions. The approach enabled meaningful influence on the workshop’s early and mid-stage ordering, while preserving the integrity of the in-person consensus process.

## Results

**Fig. 2:**
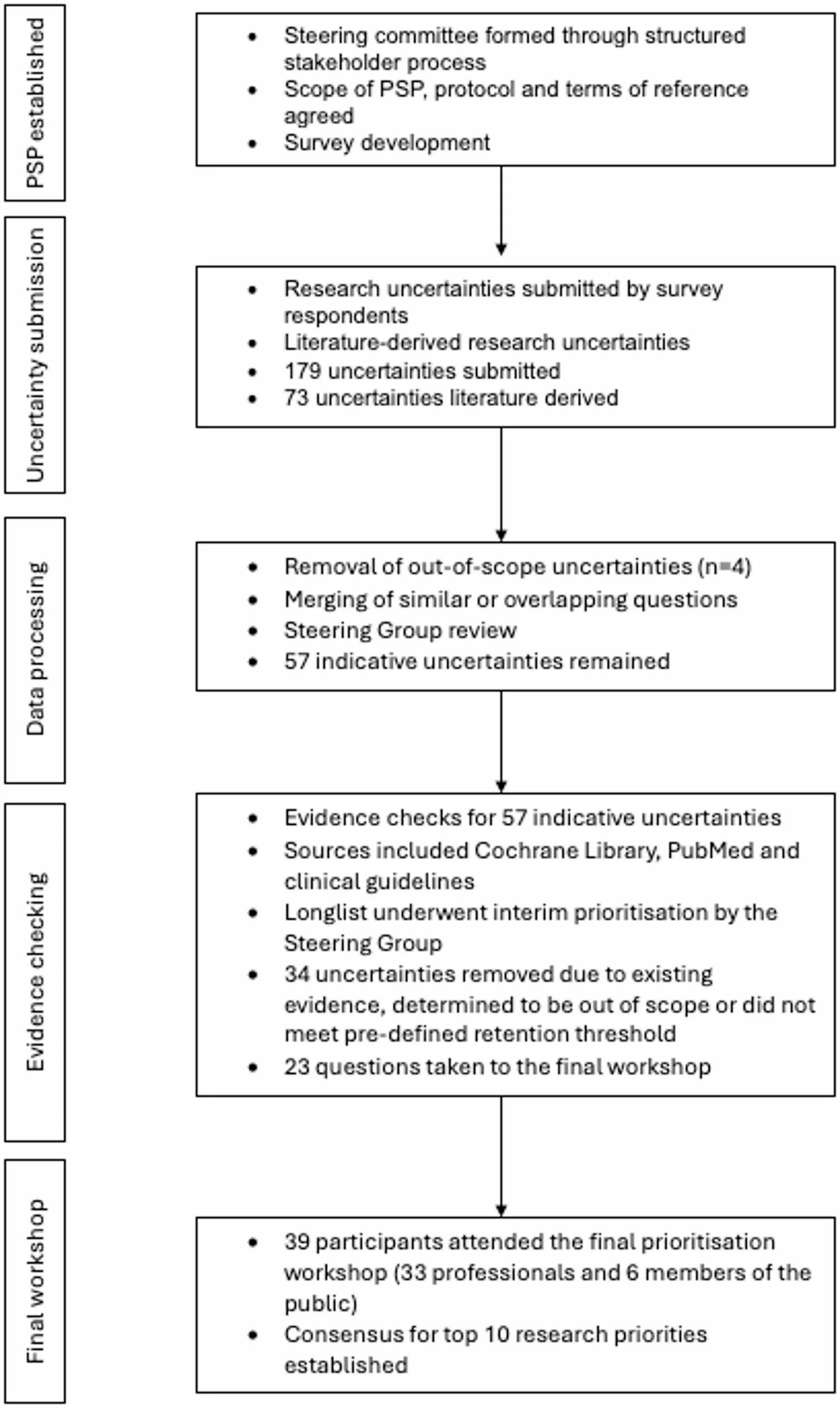
Flow chart of priority setting partnership process

Progression of uncertainties through the PSP is summarised in Fig. 2.

Progression of uncertainties through the PSP is summarised in Fig. 2. In total, 179 uncertainties were submitted via the online survey and 73 additional uncertainties were derived from the targeted literature review. Following consolidation of overlapping submissions and removal of out-of-scope items (*n* = 4), 57 indicative uncertainties underwent evidence checking. After this process, the Steering Group agreed a shortlist of 23 questions for discussion at the final prioritisation workshop.

During the workshop, participants worked in three facilitated small groups and independently ranked the 23 questions. Rankings were converted into ordinal point scores (1 for highest rank, 2 for second, 3 for third, and so on), which were then aggregated across the three groups and fed back to participants. Groups were then re-formed and the ranking exercise repeated using the combined results to inform discussion. Following this iterative process, all participants reconvened in plenary to review the final ordering. At this stage, two closely related questions were combined by agreement, and the final Top 10 research priorities were agreed by consensus.

### Final consensus outcome

**Figure:**
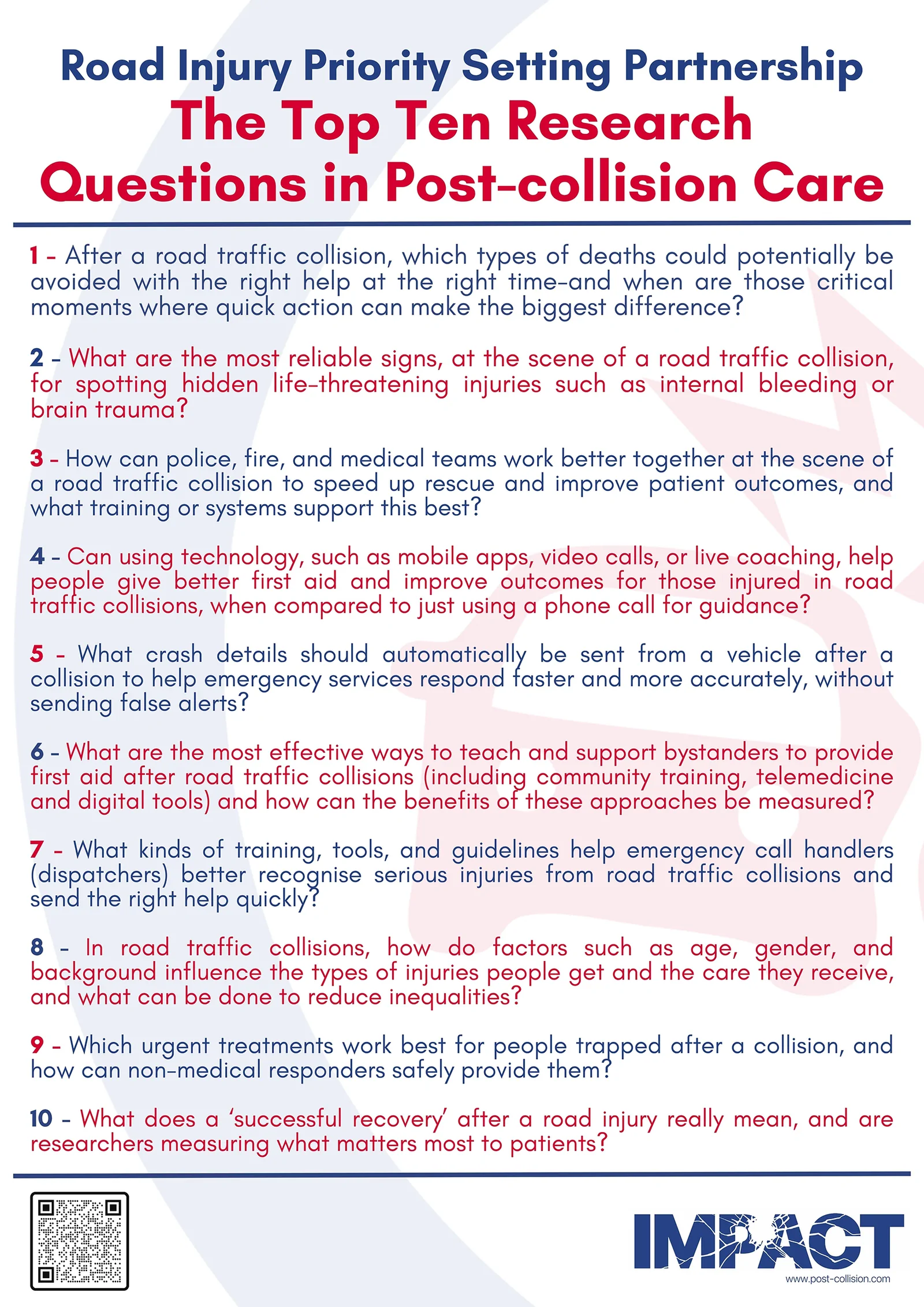

**Fig. 3:**
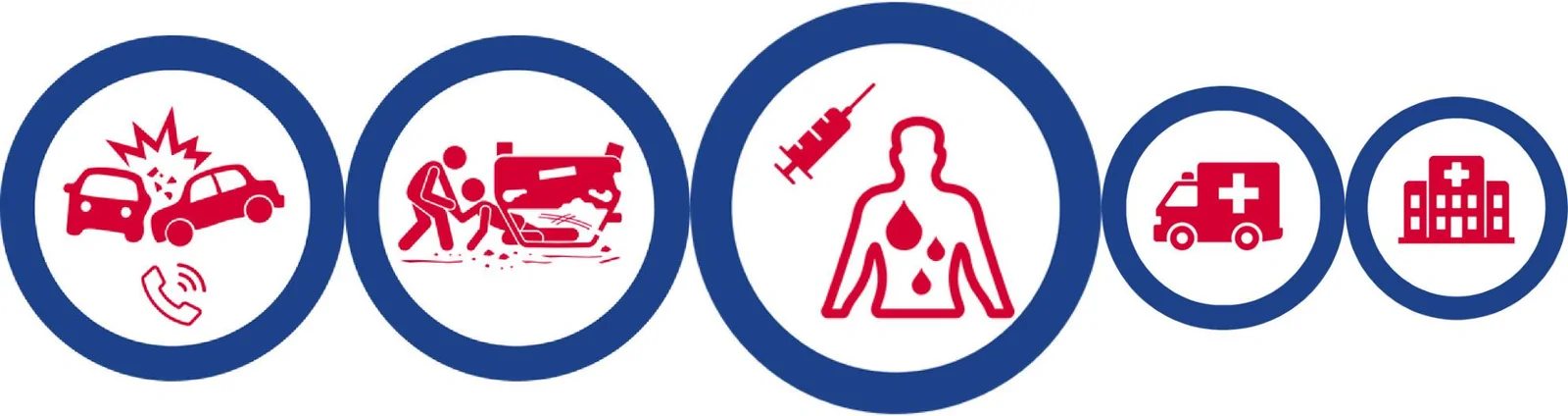
Distribution of top 10 research priorities across the Road Injury Chain of Survival Circle area is proportional to the number of Top 10 priorities addressing that link in the Road Injury Chain of Survival. Individual priorities may map to more than one link where their scope spans multiple phases of care. Three priorities were identified as cross-cutting; these include inequalities in injury patterns and care, patient-centred recovery outcomes, and integration of national collision, emergency and rehabilitation data

The ten priorities were mapped to the five links of the Road Injury Chain of Survival (Fig. 3). Several priorities addressed more than one link where their scope spanned multiple phases of care. The majority of priorities related to the first three links: Early Recognition and Call for Help, Early Rescue, and Early Initial Care. Fewer priorities mapped exclusively to Early Transport or Early Hospital and Rehabilitation.

## Discussion

### Principal findings

This national PSP has identified the ten most important unanswered research questions in post-collision care in the UK. Although the priorities collectively span the Road Injury Chain of Survival, they cluster predominantly within the first three links: Early Recognition, Early Rescue and Early Initial Care. The highest-ranked uncertainties focus on potentially preventable deaths and the identification of critical time points where rapid intervention has the greatest impact, alongside improving recognition of occult life-threatening injury such as internal haemorrhage and traumatic brain injury at the scene. A second dominant theme relates to system coordination and decision-making, including how police, fire and medical services can work more effectively together, how emergency call handlers can be better supported to recognise serious injury, and how automatic crash notification and vehicle-generated data might enable faster and more accurate emergency response.

Participants also prioritised the role of bystanders and technology in strengthening early care, highlighting the need to understand how community training, telemedicine, live video guidance and digital tools can improve first aid delivery and patient outcomes. A further priority concerned urgent treatments for people who are trapped following a collision and how non-medical responders might safely deliver time-critical interventions. Three priorities were identified as explicitly cross-cutting, spanning the entire Road Injury Chain of Survival. These overarching questions address system-level determinants of outcomes rather than isolated points of care. The first concerns inequalities in injury patterns and care, emphasising the importance of understanding how factors such as age, sex, gender and background influence both risk and treatment, and how disparities might be reduced. The second focuses on recovery, underscoring the need to define success in terms that reflect what matters most to patients themselves and to ensure that future research measures outcomes that are meaningful to survivors. The third addresses the integration of national road traffic collision, emergency and rehabilitation data, examining how linking datasets through tools such as telematics, patient identifiers and shared records could improve system performance, care quality, research capability and long-term recovery following road injury.

### Strengths and weaknesses in relation to other studies

PSPs have been completed across multiple related areas, including trauma and emergency care [11, 12]. Two previous exercises have examined post-collision care specifically [13, 14]. The Western Cape PSP in South Africa highlighted challenges around Emergency Medical Services safety, communication and major incident readiness, reflecting the operational pressures and resource constraints faced by responders in that setting [13]. The US national post-collision research initiative was developed largely through expert consensus and focused on system design, data integration and technological development [14]. When considered alongside these studies, the present UK PSP offers a complementary perspective shaped by the structure and capabilities of the contemporary UK trauma network. Together, these differing emphases illustrate how post-collision research needs to vary across contexts and how each exercise contributes distinct insights relevant to improving care at a global level.

### Meaning of the study: implications for clinicians or policymakers

The priorities identified through this PSP point towards a need for renewed focus on the earliest phases of post-collision care. Collectively, they suggest that the most significant opportunities to improve survival, reduce harm and promote equitable outcomes lie before hospital arrival, where recognition of life-threatening injury, early decision-making and coordinated rescue have the greatest influence on patient trajectories [15, 16]. Several priorities indicate the need for clearer operational frameworks across emergency services, including shared situational awareness, interoperable communication and coordinated scene management. However, these findings underline the importance of viewing post-collision care not as a series of isolated professional actions but as a system-wide response that depends on consistent collaboration between all emergency services, National Highways traffic officers, bystanders, and 999 dispatch. For policymakers, these insights highlight areas where national guidance, training standards, and joint operating procedures may benefit from further development.

The PSP also signals increasing public and professional readiness for the considered use of technology to augment early care. Interest in remote guidance, enhanced call-handling, and automatic crash notification systems suggests that future improvements may depend on integrating digital tools in ways that support, rather than replace, human decision-making. Importantly, an explicit top ten priority around inequalities underlines that system improvement must be accompanied by attention to sex, gender, age, and other social and demographic determinants of both injury and care. This emphasis aligns with wider national conversations on equitable trauma systems and points to opportunities for targeted interventions and more representative datasets. Finally, the presence of a recovery-focused priority demonstrates the importance of aligning research and service design with outcomes that are meaningful to patients themselves. This indicates that improvements in early care should be understood not only in terms of survival but also in terms of long-term function, participation and quality of life; an important message for service planners, commissioners and researchers seeking to optimise the post-collision care pathway.

### Strengths and weaknesses

A key strength of this PSP is the breadth and diversity of stakeholder involvement, incorporating the perspectives of patients with lived experience of serious injury, bystanders, emergency services, clinicians, researchers and policy representatives. The process followed a published protocol, supporting transparency and reproducibility. Adapting the approach to enable remote participation for individuals unable to travel because of injury or care needs broadened representation and mitigated the risk of excluding voices central to post-collision care. Evaluation feedback from patient participants was positive, with contributors reporting that they felt their voices were heard and that their views were meaningfully represented within the prioritisation process (Supplementary materials). This supports the acceptability of the methodological adaptations and reinforces the value of embedding patient involvement throughout priority setting in post-collision care.

Several limitations must be acknowledged. While remote contributions informed the workshop, these participants were small in number and unable to join real-time deliberation, and some nuance may have been lost as a result. As with most PSPs, self-selection may have influenced who responded to the survey and who attended the workshop, potentially under-representing certain demographic or professional groups. The process of merging complex uncertainties into single indicative questions, although necessary, inevitably simplifies issues that may be multifaceted or context dependent. It was also noted in the final prioritisation workshop that many of the priorities could be perceived as linked or interdependent and may rely on research in one area to inform another. Whilst this was acknowledged during the prioritisation process, the workshop aim was to consider each research priority in isolation in order to achieve a Top 10.

Importantly, prioritisation was based on perceived importance to patients, the public and professionals, rather than on assessments of feasibility, cost or ease of delivery. As such, the Top 10 should be understood as a statement of what matters most, rather than what is necessarily simplest to study. That said, many of the priorities are well suited to mixed-methods, observational, implementation and systems-based research designs and are likely to be deliverable within moderate budgets over a medium-term timescale. This creates a clear opportunity for funders, policymakers and research organisations to align commissioning with questions of greatest relevance to those directly affected by road injury.

Finally, the priorities identified reflect the structure and specificity of the UK trauma system; while they offer insights of wider relevance, they may not generalise directly to settings with different resources, risks or care pathways.

### Unanswered questions and future research

The priorities identified through this PSP provide a foundation for future research commissioning, policy development and system improvement across the post-collision care pathway. Dissemination will occur through national professional bodies, emergency service networks, patient groups, road safety organisations and academic channels, ensuring that the findings are accessible to those involved in post-collision care and research. The priorities will also inform the work of IMPACT and partner organisations as they develop research bids, training initiatives and collaborative programmes aligned with these evidence gaps. Monitoring the uptake and influence of the priorities will form part of ongoing activity, with future review planned to assess progress, identify areas of conceptual, instrumental and/or capacity building impacts and determine whether emerging evidence warrants priority refinement.

## Conclusions

This national road safety PSP has identified the key unanswered research questions in post-collision care, as defined by people with lived experience, bystanders, emergency responders, clinicians, researchers and policy stakeholders. The priorities focus on early recognition, coordinated rescue, patient-centred recovery and reducing inequalities across the UK post-collision care pathway, providing an inclusive foundation for future research, policy and system improvement.

## Supplementary Information

Below is the link to the electronic supplementary material.

## Data Availability

All data generated or analysed during this study are included in this published article [and its supplementary information files].
