# Correction: Identifying research priorities for post-collision care in the United Kingdom: outcomes and methodological adaptations from the final prioritisation workshop

**DOI:** 10.1186/s13049-026-01671-9

**Published:** 2026-09-01

**Authors:** Tim Nutbeam, Laura Cottey, Kerry Dungay, Lauren R. Rodgers, Emily Foote, Caroline Leech, Claire E. Baker, Elizabeth Box, Louise Johnson, Brian Lee, Marilyn MacQueen, Rob Fenwick, Nigel Lang, Ianto Guy, Ian Marritt, Ian Dunbar, Ed B. G. Barnard, Mini -Review Authors

**Affiliations:** 1 IMPACT, Centre for Post-Collision Research Innovation and Translation, Exeter, UK; 2 University of Plymouth, Plymouth, UK; 3 Academic Department of Military Emergency Medicine, Royal Centre for Defence Medicine, Birmingham, UK; 4 The Emergency Department, University Hospitals Plymouth NHSTrust, Plymouth, UK; 5 Devon Air Ambulance Trust, Exeter, UK; 6 University Hospitals Coventry & Warwickshire NHS Trust, Coventry, UK; 7 The Air Ambulance Service, Rugby, UK; 8 I-X and Dyson School of Design Engineering, Imperial College London, London, UK; 9 RAC Foundation, 89-91 Pall Mall, London, UK; 10 Leeds Teaching Hospitals NHS Trust, Leeds, UK; 11 Betsi Cadwaladr University Health Board, Wrexham Maelor Hospital, Croesnewedd Road, Wrexham, UK; 12 Wrexham University, Wrexham, UK; 13 Transport Research Laboratory, Birmingham, UK; 14 United Kingdom Rescue Organisation, World Rescue Organisation, Kingston upon Hull, UK; 15 Humberside Fire and Rescue, Kingston upon Hull, UK; 16 Federation Internationale de l’Automobile (FIA), Geneva, Switzerland; 17 Ian Dunbar Training and Consultancy, Hereford, UK; 18 IDEX Fire and Safety, Hereford, UK; 19 Emergency and Urgent Care Research in Cambridge (EUReCa), PACE Section, Department of Medicine, Cambridge University, Cambridge, UK

**Correction: Scandinavian Journal of Trauma, Resuscitation and Emergency Medicine (2026) 34:127**

**https://doi.org/10.1186/s13049-026-01628-y**

In this article the mini-review author’s name Kershaun Mathew was incorrectly written as Kershun Mathew.

The original article has been corrected.

