# Supplementary material for: Identifying research priorities for post-collision care in the United Kingdom: outcomes and methodological adaptations from the final prioritisation workshop
Source: Scand J Trauma Resusc Emerg Med. 2026 May 27;34:127. doi: 10.1186/s13049-026-01628-y (PMC13397751; doi:10.1186/s13049-026-01628-y)
Supplement: Supplementary file 2 — Supplementary Material 2 [file 13049_2026_1628_MOESM2_ESM.docx]

# REPRISE Reporting Checklist Identifying Research Priorities for Post-Collision Care in the United Kingdom: Outcomes and Methodological Adaptations from the Final Prioritisation Workshop

Adapted from: *Tong, A., Synnot, A., Crowe, S., Hill, S., Matus, A., Scholes-Robertson, N., Oliver, S., Cowan, K., Nasser, M., Bhaumik, S., Gutman, T., Baumgart, A., & Craig, J. (2019) 'Reporting guideline for priority setting of health research (REPRISE)', BMC Medical Research Methodology, 19(1). Available at: 10.1186/ s12874-019-0889-3*

| **REPRISE Item** | **How addressed in manuscript** | **Manuscript section(s)** |
| --- | --- | --- |
| 1. Geographical scope | UK-wide Priority Setting Partnership | Abstract; Methods – Scope |
| 2. Health area / field | Post-collision care across the Road Injury Chain of Survival | Background; Methods |
| 3. Intended beneficiaries | People injured on UK roads, survivors, carers | Background |
| 4. Target audience | Researchers, funders, policymakers, guideline developers | Background |
| 5. Research area | Emergency care, prehospital care, trauma systems, rehabilitation | Background |
| 6. Type of research questions | Diagnosis, systems, treatment, bystander care, recovery | Results; Discussion |
| 7. Time frame | Medium-term relevance with future review planned | Discussion – Future research |
| 8. Leadership and governance | Steering Group oversight and governance structure | Methods – Steering Group |
| 9. Team characteristics | Multidisciplinary team including patients and professionals | Author list; Methods |
| 10. Relevant training/experience | Use of JLA methodology and independent facilitation | Methods |
| 11. Framework used | James Lind Alliance Priority Setting Partnership | Methods |
| 12. Inclusion criteria for stakeholders | Patients, carers, bystanders, clinicians, responders, policy | Methods |
| 13. Stakeholder recruitment | Open national survey and partner dissemination | Methods – Collection of uncertainties |
| 14. Number of participants | 39 participants in final workshop | Results – Workshop attendance |
| 15. Stakeholder characteristics | Breakdown by professional and public groups | Results – Table 1 |
| 16. Reimbursement | Support, facilitation and preparatory briefing provided | Methods – Workshop preparation |
| 17. Collection of priorities | National survey and targeted literature review | Methods |
| 18. Collation and categorisation | Grouping, merging and scope filtering with decision trail | Methods – Data processing |
| 19. Modification of priorities | Removal of out-of-scope and answered questions with rationale | Methods – Evidence checking |
| 20. Refinement into questions | Indicative uncertainties rewritten into plain English | Methods – Shortlisting |
| 21. Evidence checking | BestBETs-style evidence checking | Methods – Evidence checking |
| 22. Number of questions | 179 → 57 → 23 → Top 10 progression | Results; Figure 2 |
| 23. Prioritisation methods | Nominal group technique, ranking and plenary consensus | Methods – Final prioritisation |
| 24. Exclusion thresholds | Median score ≥3.5 threshold for progression | Methods – Shortlisting |
| 25. Output format | Top 10 research priorities mapped to Chain of Survival | Results; Figure 3 |
| 26. Process evaluation | Positive participant feedback reported | Discussion – Strengths |
| 27. Feedback to stakeholders | Dissemination through networks and organisations | Discussion – Future research |
| 28. Implementation strategy | Alignment with funders, policy and commissioning | Discussion – Implications |
| 29. Impact evaluation plans | Planned monitoring and review of uptake | Discussion – Future research |
| 30. Funding sources | Transport Scotland and Vision Zero South West | Funding |
| 31. Conflicts of interest | Declared and managed via Steering Group | Declarations |
