# Supplementary material for: Identifying research priorities for post-collision care in the United Kingdom: outcomes and methodological adaptations from the final prioritisation workshop
Source: Scand J Trauma Resusc Emerg Med. 2026 May 27;34:127. doi: 10.1186/s13049-026-01628-y (PMC13397751; doi:10.1186/s13049-026-01628-y)
Supplement: Supplementary file 3 — Supplementary Material 3 [file 13049_2026_1628_MOESM3_ESM.docx]

## Group 1: Bystander Emergency Recognition and Calling for Help

*What are the barriers to immediate bystander emergency calls following road traffic collisions, and what interventions (educational, legal, or systemic) are most effective in reducing call delay times across diverse populations?*

**Review**

**Barriers to Immediate Calling**

Fear & Low Confidence: Many bystanders lack knowledge of emergency procedures. For example, in Ghana, although the majority would help crash victims, over half reported not knowing what to do, and around 60% cited concerns about slow or complex dispatch systems.

Distrust & Legal Worries: In various settings, individuals hesitate to call due to fear of police involvement, financial liability, or immigration consequences. A U.S. study on 911 use identified key deterrents including distrust of authorities and concerns about cost or deportation.

Environmental & Scene Factors: Chaos at accident sites, difficulty finding precise locations, and noisy or crowded environments can hinder emergency calls. Providers in Tehran emphasised issues with roadside communication and coordination.

**Effective Interventions**

Education & Public Campaigns: Training programs that simplify knowledge—how and when to dial emergency lines—prove effective. Emphasis on “what to say” and basic first aid boosts confidence and reduces hesitation.

Legal Protections: Promoting Good Samaritan or similar laws can ease fears about legal consequences. Awareness initiatives linked to these laws may increase calls and bystander engagement.

Systemic Improvements: Enhancing dispatcher protocols, implementing GPS-enhanced dispatch, and optimising call handling processes reduce confusion and inquiry overhead. Clear, structured guidance improves call clarity and urgency.

**Gaps in the Evidence**

Despite these promising interventions, the literature shows two gaps:

- Outcome Measurement: Few studies have systematically measured whether these interventions reduce actual call delays, EMS response times, or improve health outcomes.
- Long-Term Impact: There's limited evidence on whether training or legal reforms sustain improved bystander behaviour over months or years, across diverse cultural environments.

**An understanding of UK specific challenges and potential solutions**

Future research should test integrated intervention models—combining simple public education, legal assurances, dispatcher training, and improved infrastructure. Rigorous evaluation across different countries and cultures is needed to determine effectiveness in reducing call delays and improving post-crash survival.

**Plain English Summary**

People often hesitate to call emergency services after a road crash. Some don’t know what to do, fear legal trouble, or worry about police, cost, or immigration issues. In busy or confusing crash scenes, it can be hard to describe the location or situation. To help, public education campaigns and first aid training can build confidence. Legal protections like Good Samaritan laws can ease fears. Improving dispatch systems—like clearer instructions and GPS—can speed up response. However, we still don’t know how well these solutions work long-term or across different countries. More research is needed to test combined, practical approaches.

**References**

1. Sasson, C., Haukoos, J.S., Ben-Youssef, L., Ramirez, L., Bull, S., Eigel, B., Magid, D.J. and Padilla, R. Barriers to calling 911 and learning and performing cardiopulmonary resuscitation for residents of primarily Latino, high-risk neighborhoods in Denver, Colorado. Annals of emergency medicine. 2015; 65(5), 545-552.
2. Khalique, N. and Ahmad, A. Facilitators and barriers influencing the post-crash emergency care of road traffic injuries in district Aligarh of Uttar Pradesh. Journal of family medicine and primary care. 2022; 11(10), 5934-5939.

## Group 2: Public Access to Life-Saving Medications and Equipment

*What is the effectiveness, acceptability, and safety of public access tranexamic acid (TXA) administration by untrained bystanders in road traffic collision casualties with suspected haemorrhage?*

**Review**

Tranexamic acid (TXA) is one of the few interventions for bleeding trauma patients that has consistently demonstrated a clear mortality benefit. The landmark CRASH-2 trial established that early intravenous administration of TXA significantly reduces the risk of death from bleeding in trauma patients. This benefit is most pronounced when the drug is given within three hours of injury, with diminishing returns—and potential harm—if administered later. TXA is also cost-effective and safe, making it a valuable component of haemorrhage control strategies in both high- and low-resource settings.

Subsequent studies have extended these findings to the prehospital environment. Prehospital TXA has been associated with improved early survival, prolonged time to death, and reduced overall mortality in civilian trauma systems. Importantly, a 2-gram bolus administered out-of-hospital to patients with traumatic brain injury has demonstrated a reduction in 28-day mortality and improved neurological outcomes compared to both placebo and standard dosing regimens. These results have encouraged interest in ensuring the earliest possible delivery of TXA, ideally at or near the point of injury.

Recent pharmacokinetic data show that intramuscular (IM) administration of TXA achieves therapeutic plasma concentrations rapidly—often within minutes—raising the possibility that IM TXA may be a viable and potentially preferred alternative to intravenous routes. This is particularly relevant in time-critical settings or in circumstances where intravenous access is difficult or delayed, such as in rural environments, mass casualty events, or entrapment scenarios.

If scheduling changes allow for broader non-intravenous use, IM TXA could be administered not only by professionals but also by trained lay responders or bystanders. This could extend the window of effective haemorrhage control and significantly improve access to life-saving care for road traffic collision victims, particularly in settings with prolonged response times. As such, IM TXA offers a compelling opportunity to expand the reach of one of trauma care’s few proven mortality-reducing interventions.

**Plain English Summary**

Tranexamic acid (TXA) is a medicine that helps stop severe bleeding after injury and is one of the few treatments proven to save lives. It works best when given early—ideally within three hours. New research shows that TXA can be safely and quickly given by injection into a muscle, which means it might soon be possible for trained bystanders or first responders to give it before emergency services arrive. This could be especially important for people hurt in road crashes, helping to reduce deaths while waiting for hospital care.

**References**

1. Crash-2 Collaborators. The importance of early treatment with tranexamic acid in bleeding trauma patients: an exploratory analysis of the CRASH-2 randomised controlled trial. The Lancet. 2011; 377(9771), 1096-1101.
2. Grassin-Delyle, S., Shakur-Still, H., Picetti, R., Frimley, L., Jarman, H., Davenport, R., McGuinness, W., Moss, P., Pott, J., Tai, N. and Lamy, E. Pharmacokinetics of intramuscular tranexamic acid in bleeding trauma patients: a clinical trial. British journal of anaesthesia. 2021; 126(1), 201-209.
3. McKinley, W.I., Lazaridis, C., Mansour, A., Hoefer, L., Polcari, A., Benjamin, A., Schreiber, M. and Rowell, S.E. Association between prehospital tranexamic acid and cerebral edema in patients with moderate or severe traumatic brain injury. Journal of Trauma and Acute Care Surgery. 2025; 98(5), 794-797.

## Group 3: Bystander First Aid Training and Effectiveness

*What are the most effective methods and delivery models for training the public in trauma first aid, and does community-wide implementation reduce road traffic collision mortality?*

**Review**

Community-based first aid training plays a critical role in improving emergency preparedness and reducing mortality from road traffic collisions (RTCs), especially in low-resource and high-burden settings. Such training programmes typically include basic trauma care; focusing on airway, breathing, and circulation (ABCs), bleeding control, fracture management, and disaster response principles and aim to equip laypersons, first responders, and key community figures with practical skills to stabilise patients before professional help arrives (1).

The Emergency First Aid Responder (EFAR) model, developed for resource-limited areas in South Africa, is an example of sustainable community integration. It trains community members to provide immediate assistance in emergencies before ambulance arrival. Research by Sun and Wallis (2) demonstrated that EFAR participants not only retained key skills over time but also regularly applied them in real emergencies, particularly road traffic injuries, thereby improving local trauma care capacity.

In Indonesia, a targeted first aid training programme for police officers, a key group often first on scene at RTCs, found significant improvements in both theoretical knowledge and practical skills in assisting crash victims post-training.

Together, these findings support broader adoption of community-based trauma first aid education. To maximise impact, training should be context-specific, co-developed with emergency services, and embedded into a broader emergency care system. Programmes that leverage existing social structures—such as police, religious groups, and schools—may offer effective, scalable solutions in both urban and rural contexts.

**Plain English Summary**

Teaching first aid to people in the community helps save lives after road crashes, especially in places where ambulances take time to arrive. Training includes how to stop bleeding, open airways, and treat serious injuries. In South Africa, local volunteers trained as Emergency First Aid Responders kept their skills and used them to help crash victims. In Indonesia, police officers given first aid training became better at helping after road accidents. Studies show that building first aid knowledge across a community, especially when linked with local emergency services, can reduce deaths and improve how quickly and safely people are helped after crashes.

**References**

1. Fatoni F, Panduragan SL, Sansuwito T, Pusporini LS. Community first aid training for disaster preparedness: a review of education content. KnE Life Sciences. 2022 Feb 7:549-58.
2. Sun JH, Wallis LA. The emergency first aid responder system model: using community members to assist life-threatening emergencies in violent, developing areas of need. Emergency medicine journal. 2012 Aug 1;29(8):673-8.

## Group 4: Technology-Enhanced Bystander Support

*Do technology-enhanced interventions (mobile apps, video calling, real-time coaching) improve bystander first aid performance and patient outcomes in road traffic collisions compared to standard telephone guidance?*

**Review**

Technology-enhanced interventions are increasingly used to support bystanders during medical emergencies. These include mobile applications, real-time video calling, and dispatcher-assisted coaching via audio or video. Most existing evidence comes from studies on out-of-hospital cardiac arrest (OHCA), where interventions like mobile app alerts for trained volunteers and simplified CPR instructions have improved key outcomes; such as bystander CPR rates, return of spontaneous circulation, and survival to hospital discharge (1, 2).

These technologies may deliver faster, clearer, or more confidence-boosting guidance compared to traditional telephone-only dispatcher support. Real-time video, for example, allows dispatchers to observe the scene and provide tailored advice. Feedback-enabled CPR apps or devices can also support better chest compression quality. However, some studies highlight potential downsides. Using a smartphone for guidance may cause delays in CPR initiation due to the time required to activate or understand the app (2). Moreover, the majority of current tools are developed for cardiac arrest scenarios and do not account for the broader range of injuries encountered in road traffic collisions (RTCs).

When it comes to trauma-specific emergencies such as bleeding control, airway support, or scene safety, the evidence is limited. A post-hoc analysis by Bakke et al. (3) found that dispatcher telephone guidance during trauma events did not significantly improve bystander first aid performance, although it may support some life-saving interventions, particularly when the caller is untrained. Promising innovations are in development, including mobile platforms that dispatch trained responders, deliver just-in-time video tutorials, or use augmented reality to guide interventions such as applying tourniquets.

In summary, technology-enhanced bystander support shows considerable promise, especially in cardiac arrest, but its effectiveness in trauma and RTCs remains under-explored. To optimise outcomes, further research is needed to tailor interventions to the complex needs of trauma care and ensure that they are fast, accessible, and easy to use under pressure.

**Plain English Summary**

New technology like apps, video calling, and real-time coaching can help bystanders give better first aid in emergencies. These tools have been shown to improve survival after cardiac arrest by helping people start CPR quickly and do it properly. However, most tools focus on heart-related emergencies, not the kinds of injuries seen after road crashes. For trauma, evidence is still limited. Some studies show dispatcher help by phone may support life-saving actions, but results are mixed. More research is needed to develop technology that works in crash situations and gives clear, fast help for bleeding, airway problems, and other trauma injuries.

**References**

1. Tong Q, Zhou M, Liu X, Long J, Li L, Pan X, Gao H, Hu R. Mobile applications enhance out-of-hospital cardiac arrest outcomes: a systematic review and meta-analysis. BMC Health Services Research. 2025 Feb 15;25(1):256.
2. Chen KY, Ko YC, Hsieh MJ, Chiang WC, Ma MH. Interventions to improve the quality of bystander cardiopulmonary resuscitation: A systematic review. PLoS One. 2019 Feb 13;14(2):e0211792.
3. Bakke HK, Steinvik T, Ruud H, Wisborg T. Effect and accuracy of emergency dispatch telephone guidance to bystanders in trauma: post-hoc analysis of a prospective observational study. Scandinavian journal of trauma, resuscitation and emergency medicine. 2017 Mar 7;25(1):27.

## Group 5: Lay Extrication and Self-Extrication

*Under what circumstances is lay-assisted or self-extrication safer and more beneficial than waiting for professional rescue, and how can bystanders identify when immediate extrication is indicated?*

**Review**

Recent research has prompted a re-evaluation of traditional extrication strategies following motor vehicle collisions (MVCs), shifting the emphasis toward patient-focused, evidence-informed approaches. A growing body of evidence supports self-extrication as the preferred method for most patients, provided it is safe and feasible. This approach reduces entrapment time, minimises movement-related risks, and enables quicker access to definitive care. Nutbeam et al. (1) recommend self-extrication across all age groups unless the patient is unable to exit the vehicle independently due to injury, structural obstruction, or environmental hazards.

To support this decision-making process, the U-STEP OUT algorithm has been developed and endorsed. It guides emergency responders in assessing whether a patient can safely exit a vehicle unaided or if assistance is required. The algorithm accounts for physiological status, injury patterns, and scene safety, promoting a structured, reproducible approach to extrication planning (1).

For patients who cannot self-extricate—particularly older adults—research advises a minimally invasive extrication approach. Individuals aged over 80, for example, face significantly higher mortality and morbidity risks associated with prolonged entrapment and unnecessary movement. Minimally invasive techniques aim to stabilise the vehicle, reduce manipulation, and expedite transfer to care while preserving spinal and global stability (2).

Expert consensus further highlights the importance of interdisciplinary collaboration between clinical teams, fire and rescue services, and other on-scene responders. Training programmes that bring together medical and non-medical personnel are essential to reinforce a shared mental model, improve communication, and promote patient-centred care during extrication. This holistic view reflects a broader commitment to aligning rescue strategies with modern trauma care principles and available evidence (3).

In summary, extrication strategies are evolving toward minimally harmful, patient-focused approaches, with self-extrication promoted as the first-line method. This shift underscores the importance of structured algorithms, risk stratification, and collaborative training to optimise patient outcomes after MVCs.

**Plain English Summary**

New research recommends a shift in how people are rescued from crashed vehicles. If safe, patients should be encouraged to get out by themselves—called self-extrication—as it’s quicker and reduces harm. A new tool, the U-STEP OUT algorithm, helps responders decide whether this is safe. If patients can’t get out on their own, especially older adults, a gentle, low-movement rescue is best. Experts also stress the importance of teamwork between medics, fire crews, and other responders. Joint training helps everyone work better together. Overall, the focus is now on safer, faster, and more coordinated rescues tailored to the patient’s needs.

**References**

1. Nutbeam T, Fenwick R, Haldane C, Leech C, Foote E, Todd S, Lockey D. Extrication following a motor vehicle collision: a consensus statement on behalf of The Faculty of Pre-hospital Care, Royal College of Surgeons of Edinburgh. Scandinavian Journal of Trauma, Resuscitation and Emergency Medicine. 2025 Jan 6;33(1):3.
2. Nutbeam T, Kehoe A, Fenwick R, Smith J, Bouamra O, Wallis L, Stassen W. Do entrapment, injuries, outcomes and potential for self-extrication vary with age? A pre-specified analysis of the UK trauma registry (TARN). Scandinavian journal of trauma, resuscitation and emergency medicine. 2022 Mar 5;30(1):14.
3. Nutbeam T, Fenwick R, Smith JE, Dayson M, Carlin B, Wilson M, Wallis L, Stassen W. A Delphi study of rescue and clinical subject matter experts on the extrication of patients following a motor vehicle collision. Scandinavian journal of trauma, resuscitation and emergency medicine. 2022 Jun 20;30(1):41.

## Group 6: Specialist Vehicle Extrication Techniques

*What are the most effective rescue techniques for specialist vehicles (HGVs, military vehicles) and which elements of professional rescue protocols can be safely adapted for bystander use?*

**Review**

Despite the growing focus on extrication science, the literature specifically addressing rescue from specialist vehicles—such as heavy goods vehicles (HGVs) and military vehicles—is limited. These vehicles present unique challenges, including elevated cabins, greater structural mass, and restricted access routes, often requiring different tools, techniques, and responder positioning.

Professional rescue approaches emphasise vehicle-specific strategies, including stabilisation techniques for high-centre-of-gravity vehicles and adapted access points via steps, ladders, or windscreen removal. Techniques often rely on coordinated crew actions and specialised equipment such as hydraulic platforms, long-reach stabilisers, and custom entry tools.

The Vidal-Gomel et al. (1) study contributes insight into the driving and operational dynamics of heavy rescue vehicles but does not address extrication directly. However, it underscores the complexity and collective nature of emergency operations involving such vehicles. Key findings include the importance of shared situational awareness, communication, and cognitive synchronisation between team members—elements difficult to replicate in untrained bystanders.

At present, no validated bystander-adapted protocols exist for extricating occupants from HGVs or military vehicles. Existing bystander interventions—such as self-extrication guidance or basic scene safety—may be difficult to apply safely in these contexts due to height, mass, and stability concerns. Even stabilisation techniques (e.g. choking or cribbing) may not be feasible without appropriate training and equipment.

This reveals a substantial gap in both research and practice: the need for bystander-accessible guidance for complex vehicle types. Future work should consider whether simplified tools (e.g. step stools, tethered ladders), targeted public education, or remote coaching (via 999) could support safe layperson action in the first minutes after impact. Rigorous simulation-based studies are needed to test feasibility, safety, and psychological impact.

**Plain English Summary**

Rescuing people from lorries or military vehicles is much harder than from regular cars because the vehicles are bigger, higher, and harder to access. Professional rescue teams use ladders, tools, and teamwork to help people safely. However, there’s very little guidance for members of the public who arrive first. Unlike in car crashes, bystanders can’t easily help due to the height and size of these vehicles. More research is needed to see if simple tools or training—like using a small ladder or giving advice over the phone—can help bystanders assist safely before emergency services arrive.

**References**

1. Vidal-Gomel C, Delgoulet C, Gébaï D. Specialisation and training for fire-fighters driving heavy rescue vehicles: consequences for the development of operators?. Work. 2012 Mar;41(S1):5177-83.

## Group 7: Extrication Time Thresholds and Impacts

*What are the critical time thresholds for extrication beyond which mortality and morbidity (including psychological outcomes) increase significantly, and how do different extrication methods impact these outcomes?*

**Review**

Research on vehicle extrication and trauma outcomes highlights that trapped motor vehicle collision (MVC) patients experience higher mortality, more severe injuries, and worse physiological status than non-trapped counterparts (1). Prehospital time has long been recognised as a factor influencing trauma outcomes; however, most studies focus narrowly on in-hospital mortality, with limited consideration of morbidity, long-term function, or psychological sequelae (2,3).

Despite widespread clinical and operational interest, the evidence base lacks clearly defined critical time thresholds for extrication beyond which morbidity or mortality increases significantly. Without time-stamped, high-resolution data capturing the moment of entrapment through to release, it remains difficult to determine when delays begin to cause harm. Similarly, there is limited understanding of the specific physiological or psychological tipping points associated with prolonged entrapment.

A Delphi consensus study (4) outlined principles of patient-centred extrication, including the need to balance speed with safety. However, it did not establish evidence-based durations or compare the impact of different extrication methods on long-term outcomes.

**Key gaps in the literature**

- Lack of validated time thresholds for extrication linked to morbidity or psychological harm.
- Minimal prospective or longitudinal studies examining outcomes such as PTSD, chronic pain, or disability.
- Limited comparative studies of extrication techniques in terms of speed, safety, and patient-centred outcomes.
- Future research should prioritise linking extrication timelines and methods to multidimensional outcomes across physical, functional, and psychological domains.

**Plain English Summary**

Studies show that people trapped in cars after crashes are more seriously hurt and more likely to die than those who aren’t trapped. Long delays before they are rescued may make things worse, but research hasn’t yet found the exact amount of time after which harm increases. Most studies look only at death rates and not other problems like long-term pain or emotional trauma. It’s also unclear which rescue methods are safest and quickest. More research is needed to understand how long is too long to be trapped, and whether faster, simpler rescue techniques could improve recovery and save lives.

**References**

1. Nutbeam T, Fenwick R, Smith J, Bouamra O, Wallis L, Stassen W. A comparison of the demographics, injury patterns and outcome data for patients injured in motor vehicle collisions who are trapped compared to those patients who are not trapped. Scandinavian journal of trauma, resuscitation and emergency medicine. 2021 Jan 14;29(1):17.
2. Bedard AF, Mata LV, Dymond C, Moreira F, Dixon J, Schauer SG, Ginde AA, Bebarta V, Moore EE, Mould-Millman NK. A scoping review of worldwide studies evaluating the effects of prehospital time on trauma outcomes. International journal of emergency medicine. 2020 Dec;13(1):64.
3. Chen CH, Shin SD, Sun JT, Jamaluddin SF, Tanaka H, Song KJ, Kajino K, Kimura A, Huang EP, Hsieh MJ, Ma MH. Association between prehospital time and outcome of trauma patients in 4 Asian countries: a cross-national, multicenter cohort study. PLoS medicine. 2020 Oct 6;17(10):e1003360.
4. Nutbeam T, Fenwick R, Smith JE, Dayson M, Carlin B, Wilson M, Wallis L, Stassen W. A Delphi study of rescue and clinical subject matter experts on the extrication of patients following a motor vehicle collision. Scandinavian journal of trauma, resuscitation and emergency medicine. 2022 Jun 20;30(1):41.

## Group 8: Automatic Crash Detection and eCall Systems

*What crash parameters and data elements should be transmitted through eCall systems to optimise triage accuracy and response times while minimising false positive rates?*

**Review**

To optimise triage accuracy and response times while minimising false positive rates, eCall systems—particularly those using Advanced Automatic Crash Notification (AACN)—must transmit a carefully selected set of crash parameters and data elements. These systems are designed to assess injury severity and initiate timely emergency response based on automated crash analysis, especially when occupants are incapacitated.

Key data elements transmitted through the eCall Minimum Set of Data (MSD) include crash severity metrics (e.g. delta-V, principal direction of force), restraint use (seatbelt status, airbag deployment), vehicle type and model, number of occupants, and location coordinates. These factors are crucial for estimating injury likelihood and guiding appropriate resource dispatch. Studies such as Stitzel et al. (1) demonstrate that AACN algorithms using such variables can reduce undertriage rates to under 5% and overtriage to below 50% in side-impact collisions—potentially improving outcomes for over 165,000 individuals annually.

Accurate transmission of these data relies on robust signal strength and system reliability, as explored in Öörni and Korhonen’s (2) field test of eCall systems in Finland. Transmission failure, particularly in rural areas or post-collision vehicle instability, remains a barrier to consistent MSD delivery. Integrating redundancy (e.g. dual-SIM transmission) and verifying transmission success are therefore essential.

From a dispatch optimisation perspective, Ceklic et al. (3) evaluated a predictive algorithm combining crash characteristics with Medical Priority Dispatch System (MPDS) codes. While the system achieved low undertriage rates (2.7%), the high overtriage rate (84.8%) reflects the trade-off between safety and efficiency. High overtriage can strain emergency services, suggesting a need for real-time, adaptive algorithms that consider both crash dynamics and historical outcome data.

To advance eCall utility, future AACN systems should incorporate machine learning models trained on large, linked crash and trauma datasets. This would allow continuous refinement of triage predictions and the potential inclusion of novel inputs such as occupant age, pre-crash braking behaviour, or telematics-linked health data. A harmonised European or international framework for eCall data transmission could further improve standardisation and interoperability.

In summary, effective AACN and eCall systems require transmission of high-fidelity crash, occupant, and vehicle data. Optimising triage accuracy will depend on balancing under- and overtriage rates, ensuring robust data delivery, and integrating evolving predictive technologies.

**Plain English Summary**

eCall and Advanced Automatic Crash Notification (AACN) systems help emergency services respond quickly to serious road crashes. They work by automatically sending key information—like crash severity, seatbelt use, airbag deployment, and exact location—to dispatch teams. This helps decide what level of help is needed, even if the people involved can’t call for help themselves. Research shows these systems can reduce missed serious injuries and improve response. However, they can also send too many false alarms. To improve, systems must reliably send data, even in rural areas, and use smarter algorithms that learn from past crashes to better predict who needs urgent care.

**References**

1. Stitzel JD, Weaver AA, Talton JW, Barnard RT, Schoell SL, Doud AN, Martin RS, Meredith JW. An injury severity-, time sensitivity-, and predictability-based advanced automatic crash notification algorithm improves motor vehicle crash occupant triage. Journal of the American College of Surgeons. 2016 Jun 1;222(6):1211-9.
2. Öörni R, Korhonen TO. eCall minimum set of data transmission–results from a field test in Finland. IET intelligent transport systems. 2014 Dec;8(8):639-47.
3. Ceklic E, Tohira H, Ball S, Brown E, Brink D, Bailey P, Brits R, Finn J. A predictive ambulance dispatch algorithm to the scene of a motor vehicle crash: the search for optimal over and under triage rates. BMC emergency medicine. 2022 May 6;22(1):74.

## Group 9: Dispatcher Triage and Decision Support

*What structured protocols, training interventions, and decision support tools most effectively improve dispatcher accuracy in identifying severe road traffic collision injuries and triaging appropriate resources?*

**Review**

Accurate early triage of Road Traffic Collision (RTC) injuries by emergency dispatchers is critical for timely resource allocation and optimal patient outcomes. Current research has explored a range of tools and interventions, yet significant gaps remain in developing and validating structured, road injury-specific dispatcher support systems.

Several promising innovations have emerged. Kuan-Chen Chin et al. (1) demonstrated that machine learning algorithms applied to emergency call transcripts can assist dispatchers in identifying severe injuries, particularly when human judgment is uncertain. However, these tools are largely unvalidated in diverse real-world contexts, and the models often lack transparency in decision-making—posing barriers to clinical acceptance.

Matthew Miller et al. (2) highlighted the fragmented nature of existing research, calling for standardised outcome measures and unified metrics to evaluate dispatch accuracy. Their review also noted that clinician oversight—for example, involving paramedic consultants or medical dispatch advisors—can improve triage accuracy. However, this adds resource demands and is not scalable in many low-resource or high-demand settings.

International studies reveal ongoing challenges. In South Africa and the Netherlands, both over-triage (dispatching more resources than needed) and under-triage (missing critical injuries) remain common (3,4). These studies emphasise the importance of protocol refinement and dispatcher training, yet do not delineate which components of training or protocol structures are most effective, particularly for RTCs as a distinct injury mechanism.

Gaps in the literature

- Lack of RTC-specific protocols: Most existing systems are generalised for medical emergencies and not tailored to the mechanisms and injury patterns of road traffic collisions.
- Limited integration of real-time crash data: Few systems incorporate vehicle telemetry, caller emotional state, or crash mechanism details (e.g., ejection, rollover) into triage decisions.
- Insufficient long-term outcome data: There is limited evidence linking dispatcher triage decisions to downstream clinical outcomes such as morbidity, disability, or recovery times.

**Plain English Summary**

When someone is badly hurt in a road crash, emergency call handlers (dispatchers) must quickly decide what help to send. Current systems often miss serious injuries or send too much help. Some new tools, like computer programmes that analyse what callers say, can support dispatchers, especially when they are unsure. Training and clear protocols also help, but many are not specific to road crashes. Most research comes from wealthy countries, so we don’t know what works best elsewhere. More research is needed to create simple, road-specific tools that help dispatchers send the right help, quickly and reliably, every time.

**References**

1. Chin KC, Cheng YC, Sun JT, Ou CY, Hu CH, Tsai MC, Ma MH, Chiang WC, Chen AY. Machine learning–based text analysis to predict severely injured patients in emergency medical dispatch: model development and validation. Journal of medical Internet research. 2022 Jun 10;24(6):e30210.
2. Miller M, Bootland D, Jorm L, Gallego B. Improving ambulance dispatch triage to trauma: A scoping review using the framework of development and evaluation of clinical prediction rules. Injury. 2022 Jun 1;53(6):1746-55.
3. Alshehri MF, Pigoga JL, Wallis LA. Dispatcher triage accuracy in the western cape government emergency medical services system, Cape Town, South Africa. Prehospital and Disaster Medicine. 2020 Dec;35(6):638-44.
4. Waalwijk JF, Lokerman RD, van der Sluijs R, Fiddelers AA, Leenen LP, van Heijl M, Poeze M. Priority accuracy by dispatch centers and Emergency Medical Services professionals in trauma patients: a cohort study. European journal of trauma and emergency surgery. 2022 Apr;48(2):1111-20.

## Group 10: Video-Enabled Dispatch and Visual Triage

##

*Does video-enabled dispatch improve injury severity recognition and triage accuracy compared to voice-only communication, and what are the privacy and implementation considerations?*

**Review**

Emerging research suggests that video-enabled dispatch systems may significantly improve emergency triage accuracy, particularly in recognising injury severity and tailoring the response accordingly. Compared to voice-only communication, video triage allows dispatchers and clinicians to visually assess patients and scene context, thereby reducing reliance on caller descriptions, which may be incomplete or inaccurate.

A cluster-randomised trial by Gude et al. (1) demonstrated that video triage reduced high-urgency dispatches by 5%, suggesting more precise allocation of resources. Similarly, Idland et al. (2) reported that video communication improved recognition of first aid needs in injured patients (OR 5.30). In helicopter emergency medical services (HEMS), Ulvin et al. (3) reported fewer inappropriate dispatches with video calls (28.4% vs 40.3%).

These studies suggest that video triage can refine decision-making, reduce unnecessary dispatches, and support more accurate identification of critical injury. Importantly, these improvements have been achieved without significant increases in call length or adverse patient outcomes. Moreover, both patient and provider feedback has been positive, and early evidence supports cost-effectiveness (4).

Despite promising results, several gaps remain:

- Trauma-specific validation: Most studies have not stratified by mechanism of injury. There is limited evidence specifically evaluating whether video triage improves recognition of high-severity trauma from road traffic collisions (RTCs).
- Outcome linkage: Few studies link dispatch accuracy with patient outcomes (e.g. morbidity, mortality, hospital length of stay).
- Privacy and legal frameworks: Ethical concerns—including data protection, informed consent, and video retention—are underexplored. Public comfort and legal acceptability vary across jurisdictions.
- Equity and access: Widespread implementation depends on network connectivity, smartphone availability, and digital literacy, which may disadvantage certain populations.

**Plain English Summary**

Using video during emergency calls helps dispatchers better understand how badly someone is hurt and decide what help to send. Studies show video can reduce unnecessary ambulance callouts, improve first aid advice, and help avoid sending specialist teams when they’re not needed—all without delaying care. People using video feel positive about it, and it may also save money. However, more research is needed to see how well it works specifically for serious road crashes. Questions also remain about privacy, data protection, and whether everyone has access to video technology. These issues must be addressed before video dispatch can be widely used.

**References**

1. Gude MF, Valentin JB, Meisner-Jensen M, Bohnstedt-Pedersen NH, Dalgaard AK, Væggemose U, Blauenfeldt RA. Video Streaming or Telephone Communication During Emergency Medical Services Dispatch Calls: A Cluster Randomized Clinical Trial. JAMA Network Open. 2025 Jul 1;8(7):e2519020-.
2. Idland S, Kramer-Johansen J, Bakke HK, Hagen M, Tønsager K, Platou HC, Hjortdahl M. Can video streaming improve first aid for injured patients? A prospective observational study from Norway. BMC emergency medicine. 2024 May 28;24(1):89.
3. Ulvin OE, Skjærseth EÅ, Krüger AJ, Thorsen K, Nordseth T, Haugland H. Can video communication in the emergency medical communication Centre improve dispatch precision? A before–after study in Norwegian helicopter emergency medical services. BMJ open. 2023 Oct 1;13(10):e077395.
4. Nehme, E., Magnuson, N., Mackay, L., Becker, G., Wilson, M., & Smith, K. (2023). Study of prehospital video telehealth for callers with mental health-related complaints. Emergency Medicine Journal, 40(2), 128–133. https://doi.org/10.1136/emermed-2022-212456.

## Group 11: Artificial Intelligence and Machine Learning in Dispatch

*Can artificial intelligence and machine learning technologies improve the identification of serious injuries from crash scene photos, call audio, or historical data compared to human dispatcher assessment?*

**Review**

Artificial Intelligence (AI) and Machine Learning (ML) have demonstrated considerable potential to enhance trauma care and road safety by improving injury prediction and resource allocation (1). Recent studies show that AI/ML algorithms can outperform conventional trauma triage tools in predicting outcomes such as mortality, hospitalisation, and critical care admission (2). These tools may assist in identifying serious injuries early—ideally before the arrival of first responders—using data sources such as crash photos, emergency call audio, and historical driver or crash data.

AI models have shown promise in predicting injury severity from crash parameters and vehicle telemetry (3), and in supporting dispatch decisions based on structured and unstructured data. For example, AI can process call transcripts or ambient audio to detect stress indicators or abnormal speech, potentially identifying serious cases more reliably than humans alone.

Despite these advances, several key gaps remain in the literature:

- Lack of application to image and audio data: While AI has been used to process vehicle kinematics and structured data, fewer studies have evaluated its performance in analysing crash scene images or real-time call audio—both of which may offer rich contextual clues about injury severity.
- Limited direct comparison with human dispatchers: Few studies have benchmarked AI performance against dispatcher decision-making using the same data inputs. This limits our understanding of where AI adds the most value.
- Prospective validation in real-world dispatch settings is scarce. Most models are retrospective and trained on registry data, which may not reflect the chaotic and time-pressured context of real dispatch.
- Interpretability and trust remain challenges. Complex AI models often act as "black boxes", making it difficult for dispatchers to understand or trust their recommendations without explainable outputs.
- Bias and generalisability: AI systems may inherit biases from the datasets they are trained on (e.g. under-representation of older adults or non-frontal crashes), limiting their reliability across diverse crash scenarios.

**Plain English Summary**

Artificial intelligence (AI) and machine learning (ML) could help emergency teams quickly identify serious injuries after a crash by analysing photos, call recordings, and past crash data. Some studies show AI can predict who needs hospital care better than current methods. However, there’s little research comparing AI directly to human dispatchers, especially using real crash scene images or audio. Most systems haven’t been tested in real emergencies, and some may not work well for all types of crashes or people. More research is needed to see if AI can safely and fairly support faster, more accurate emergency decisions.

**References**

1. Torbaghan ME, Sasidharan M, Reardon L, Muchanga-Hvelplund LC. Understanding the potential of emerging digital technologies for improving road safety. Accident Analysis & Prevention. 2022 Mar 1;166:106543.
2. Adebayo O, Bhuiyan ZA, Ahmed Z. Exploring the effectiveness of artificial intelligence, machine learning and deep learning in trauma triage: A systematic review and meta-analysis. Digital health. 2023 Oct;9:20552076231205736.
3. Hunter OF, Perry F, Salehi M, Bandurski H, Hubbard A, Ball CG, Morad Hameed S. Science fiction or clinical reality: a review of the applications of artificial intelligence along the continuum of trauma care. World Journal of Emergency Surgery. 2023 Mar 6;18(1):16.

## Group 12: Injury Recognition by Bystanders and First Responders

*What are the most reliable and teachable methods for training bystanders and first responders to identify life-threatening injuries and high-risk collision characteristics without clinical equipment?*

**Review**

**Simplified Triage Scores**
Nutbeam et al. (1) introduced the simplified Bleeding Audit Triage Trauma (sBATT)—a score for major trauma patients using visible indicators: age >65, altered consciousness, absent radial pulse, rapid heartbeat, or being trapped in a vehicle. It predicts 24‑hour mortality with impressive accuracy (AUC 0.90; sensitivity 96%; specificity 72%) This tool excels without equipment, making it teachable to non-medical responders.

Gap: Validation occurred only in retrospective UK data—its usability in real-world, layperson scenarios remains untested, as does effectiveness in low-income or culturally diverse settings.

**Lay Responder Training on Warning Signs**
Pellegrino et al. (2) conducted a systematic review on lay responder training outcomes and highlighted improved recognition of life-threatening bleeding following structured programmes. These sessions typically focused on visible warning signs—uncontrolled bleeding, airway obstruction, or chest injuries—which are easy for laypersons to learn.

Gap: The confidence in identifying collision-specific cues—such as ejection, rollover, or vehicle deformation—remains under-evaluated. Also, most programs lack clear metrics on real-world application beyond simulated settings.

**Simplified Visual / Auditory Cues**
While not always formalised, protocols often emphasise checking consciousness, pulse at the wrist, visible bleeding, open chest wounds, and patient responsiveness. These visual and auditory signs are intuitive but lack consistency across different training programmes.

Gap: There's a need for standardised, concise cue sets that are easy to teach, remember, and apply under stress.

Several key gaps remain in the literature:

- Real-world validation of tools like sBATT by actual bystanders in emergencies.
- Cultural and geographic adaptation: how might scores and signs translate across global contexts?
- Standardised training tools for identifying serious injuries stemming from vehicle mechanics (e.g., crushed cabin, ejection risk).

**Plain English Summary**

Teaching people how to spot life-threatening injuries after a crash can save lives. Simple tools like the sBATT score help identify serious bleeding and trauma using visible signs like confusion, fast heartbeat, or being trapped in a vehicle—without any medical equipment. Training programmes also teach bystanders to look for heavy bleeding, airway problems, and chest wounds. These signs are easy to learn but are not always taught in the same way. Gaps remain: most tools haven’t been tested with real bystanders, and few include clues from the crash scene like car damage or ejection. More real-world testing is needed.

**References**

1. Nutbeam T, Stassen W, Foote E, Ageron FX. Derivation and validation of the simplified Bleeding Audit Triage Trauma (sBATT) score: a simplified trauma score for major trauma patients injured in motor vehicle collisions. BMJ open. 2024 Dec 1;14(12):e090517.
2. Pellegrino JL, Smith SE, Banton E, Sudhir A. Systematic Review of Lay Responders Educational Outcomes to Identify Life-Threatening Bleeding. International Journal of First Aid Education. 2022 Apr 10;4(2).

## Group 13: Wearable Technology and Collision Detection

*Can wearable technology devices be validated and optimised for detecting road traffic collisions and transmitting useful information to emergency services in unwitnessed crashes?*

**Review**

Wearable technology holds significant promise in advancing road traffic safety through real-time monitoring, collision detection, and automated emergency response. These devices, which may include smartwatches, wearable sensors, and portable GPS-enabled systems, offer new pathways for early crash identification and enhanced post-collision care.

For post-crash scenarios, Sontakke and Gawande proposed a wearable crash notification system using GPS and accelerometers to automatically alert emergency services (1). This concept could reduce response times by providing precise location and impact data immediately after a collision (2).

Despite this potential, key gaps remain in the evidence base:

- Limited real-world validation: Most studies are proof-of-concept or simulation-based. There is a lack of large-scale, real-world trials demonstrating these technologies’ effectiveness in reducing injury or improving outcomes.
- User acceptability and adherence: The success of wearable technologies depends on regular use and wearer compliance, which is inconsistently studied.
- Data accuracy and false alarms: There is limited research on the sensitivity and specificity of these systems in actual crashes, particularly distinguishing true collisions from non-injury events (e.g., sudden stops or phone drops).
- Integration with emergency systems: Few studies address how wearable alerts would interface with national or regional emergency dispatch systems in real time.

**Plain English Summary**

Wearable technology, like smartwatches and body sensors, could help keep road users safer. These devices can warn drivers if they’re stressed or drowsy—two common causes of crashes. Some systems can detect a crash using movement sensors and send an automatic alert with the location to emergency services. Others track a person’s health during recovery. While these ideas are promising, most haven’t been tested in real-life crashes. It’s also unclear how well they work, how people will use them, or how they connect to ambulance services. More research is needed to see if they really save lives and improve care.

**References**

1. Sontakke SR, Gawande AD. Crash notification system for portable devices. International Journal of Advanced Computer Technology (IJACT) I. 2013 Jun;2(3):33-8.
2. Scquizzato T, Gamberini L, Semeraro F. Integrating Data From Motor Vehicle Crash Detection Systems of Smartphones and Wearable Digital Devices—The Future of Trauma Care. JAMA surgery. 2023 Sep 1;158(9):897-8.

## Group 14: Rural and Remote Collision Detection

*What is the burden of undetected collisions in rural and remote areas, and what technologies (sensors, drones, satellite monitoring) are most effective for rapid detection and response?*

**Review**

Rural and remote regions face unique challenges in post-collision response, where sparse population density, vast geography, and delayed emergency services contribute to higher rates of road trauma and mortality. Emerging technologies—particularly drones, satellite communications, and sensor networks—offer new avenues to detect collisions rapidly and deliver timely medical assistance in areas with limited infrastructure.

Recent work demonstrates the potential of drones for rapid accident detection and response, showing their ability to monitor traffic incidents in real-time, deliver essential supplies, and assist first responders with scene assessment. Their flexibility and speed make them well-suited for terrain inaccessible to ground vehicles. However, weather sensitivity and limited flight duration constrain their operational reliability in extreme conditions—a common feature of remote environments.

Complementing drone capabilities, satellite communications and high-altitude platforms enable real-time data transmission even in areas lacking terrestrial networks. Giambene et al. (1) discuss the role of such platforms in supporting IoT-based early warning systems (e.g. for wildfires), which may be adaptable to road traffic collision (RTC) contexts. Sensor integration with vehicle or roadside infrastructure, satellites, and drones—as described by Hart et al. (2)—could provide a multi-layered approach to emergency detection and response in disconnected environments.

Despite these advances, road trauma rates in regional Australia and New Zealand remain disproportionately high (3). Many of these regions lack sufficient infrastructure to support even standard vehicle safety technologies like Advanced Driver Assistance Systems (ADAS), let alone advanced aerial or satellite-based monitoring tools. Furthermore, evidence is sparse regarding real-world effectiveness: few studies link these technologies to improved patient outcomes, reduced response times, or lower mortality in rural collisions.

Gaps in the Literature

- Lack of outcome-focused studies evaluating whether these technologies improve survival or morbidity after RTCs.
- Limited integration frameworks: There is little guidance on how drones, sensors, and satellites might interface with national emergency dispatch systems.
- Scalability and cost: Research has yet to address long-term maintenance, affordability, and training for rural implementation.
- Equity concerns: Regions already underserved by basic road safety measures may lack the resources to adopt advanced systems.

**Plain English Summary**

New technologies like drones, satellites, and smart sensors could help detect car crashes faster in remote areas where emergency services are far away. Drones can quickly find crashes and send real-time information to help responders. Satellite systems can work where mobile phone networks don’t reach. Together, these tools could save lives by speeding up help. But many rural places still don’t have the basic road safety infrastructure needed to support these technologies. More research is needed to test if they really improve survival rates, how to connect them to emergency services, and how to make them affordable and reliable.

**References**

1. Giambene G, Addo EO, Kota S. 5G aerial component for IoT support in remote rural areas. In2019 IEEE 2nd 5G World Forum (5GWF) 2019 Sep 30 (pp. 572-577). IEEE.
2. Hart, J.K. and Martinez, K. (2006) Environmental sensor networks: A revolution in the earth system science?, Earth-Science Reviews, 78(3–4), pp. 177–191. doi:10.1016/j.earscirev.2006.05.001
3. Peiris S, Berecki-Gisolf J, Chen B, Fildes B. Road trauma in regional and remote Australia and New Zealand in preparedness for ADAS technologies and autonomous vehicles. Sustainability. 2020 May 26;12(11):4347.

## Group 15: Specific Interventions for Entrapped Patients

*What time-critical medical interventions (pharmacological and non-pharmacological) are most effective for entrapped patients, and how can these be optimally delivered by non-clinical responders?*

**Review**

Entrapped patients are more likely to suffer major haemorrhage, tension pneumothorax, and spinal injuries, and are more likely to require time-critical intervention (1). Evidence on treating this group is limited, based largely on low- and middle-income countries following structural collapse, and mainly consists of case series and expert consensus rather than high-quality randomised trials. Recommended pharmacological interventions include early aggressive fluid resuscitation with potassium-free solutions for those at risk of crush syndrome (2,3) and prompt hyperkalaemia treatment (2). These are thought to reduce complications, but there is no robust evidence of improved survival in this cohort.

Non-pharmacological interventions that are recommended in the literature include effective communication to support patient wellbeing (1), avoidance of hypothermia, and airway management (2). While these have strong evidence in general trauma care, data in entrapped patients are lacking.

A search on the use of lay-people to deliver interventions in entrapped patients yielded no relevant studies. Limited evidence exists for bystander involvement in prehospital trauma care, particularly in haemorrhage and airway management, with possible improvement in mortality in those with haemorrhage and airway compromise (4).

In summary, there are few evidence-based pharmacological and non-pharmacological interventions proven to improve outcomes in entrapped patients. Current practice relies on extrapolation from broader trauma literature and expert opinion. Research into bystander roles in trauma care offers insight but has not been applied to this specific cohort.

The scarcity of high-quality studies likely reflects the logistical and ethical challenges of researching physically trapped patients who require rapid extrication and intervention. Entrapment is also relatively uncommon compared with medical causes of immobility, making trial recruitment slow and unpredictable (1).

**Plain English Summary**

Entrapped patients are at higher risk of severe injuries that require urgent treatment. There is, however, little high-quality evidence for which interventions are more effective in this group. Current practice relies on wider trauma evidence and expert consensus, focusing on early fluid resuscitation and medication to mitigate the consequences of muscle breakdown. Bystanders may have a role in initial management, but evidence is lacking. Further research is needed to identify the most effective interventions and improve survival in this group.

**References**

1. Nutbeam T, Fenwick R, Smith J, Bouamra O, Wallis L, Stassen W. A comparison of the demographics, injury patterns and outcome data for patients injured in motor vehicle collisions who are trapped compared to those patients who are not trapped. Scandinavian journal of trauma, resuscitation and emergency medicine. 2021 Jan 14;29(1):17.
2. Bosson N, Abo BN, Litchfield TD, Qasim Z, Steenberg MF, Toy J, Osuna-Garcia A, Lyng J. Prehospital trauma compendium: management of the entrapped patient–a position statement and resource document of NAEMSP. Prehospital Emergency Care. 2024 Oct 14:1-3.
3. Usuda D, Shimozawa S, Takami H, Kako Y, Sakamoto T, Shimazaki J, Inoue J, Nakayama S, Koido Y, Oba J. Crush syndrome: a review for prehospital providers and emergency clinicians. Journal of Translational Medicine. 2023 Aug 31;21(1):584.
4. Tannvik TD, Bakke HK, Wisborg T. A systematic literature review on first aid provided by laypeople to trauma victims. Acta Anaesthesiologica Scandinavica. 2012 Nov;56(10):1222-7.

##

## Group 16: Gender and Demographic Disparities in Collision Care

*Do gender, age, and demographic factors affect triage accuracy, entrapment likelihood, and treatment outcomes in road traffic collision care, and how can disparities be addressed?*

**Review**

A growing body of evidence indicates that demographic factors—particularly age, gender, and socioeconomic status—significantly influence triage decisions, injury patterns, and outcomes following road traffic collisions (RTCs). These variables affect not only the likelihood of entrapment and injury severity but also the accuracy and appropriateness of emergency responses, contributing to disparities in care and survival.

Age plays a critical role in triage and outcomes. Older adults experience more severe injuries at lower crash velocities due to age-related physiological changes (1, 2). This vulnerability raises concerns about underestimation of injury severity in seemingly minor collisions, particularly when standard triage systems do not incorporate age-specific thresholds.

Gender disparities are also well-documented. Nutbeam et al. found that women are more likely to be entrapped following an RTC and tend to suffer distinct injury patterns—particularly to the pelvis and lower limbs—compared to men (3). These differences may reflect both vehicle design biases and physiological variation. Entrapment itself is associated with higher injury severity and mortality yet triage tools rarely account for the increased risk faced by women or other demographic groups (3).

Socioeconomic status (SES) and education levels further influence emergency care pathways. Individuals with lower SES may receive less aggressive triage and delayed interventions, possibly due to implicit biases or communication barriers (4).

Gaps in the literature

- Current triage tools lack demographic sensitivity, often using one-size-fits-all thresholds that do not account for age or gender differences in injury risk and entrapment likelihood.
- Entrapment is under-researched as a demographic-specific outcome, with few studies exploring how gender, age, or SES influence extrication times or rescue strategies.
- Interventions to address disparities—such as revised vehicle safety standards, dispatcher decision support tools, or inclusive trauma triage algorithms—are underdeveloped and largely untested.

**Plain English Summary**

Who you are can affect how you're treated after a car crash. Older people often get hurt more easily, even in low-speed crashes, and women are more likely to be trapped in vehicles and have different types of injuries than men. People with lower income or education may also face delays in getting help. Current triage systems don’t always account for these differences, which can lead to unfair care. To fix this, we need better tools that consider age, gender, and background when making emergency decisions—and safer vehicles designed with all types of people in mind.

**References**

1. Adebisi, A., Ma, J., Masaki, J. and Sobanjo, J. (2019) Age-Related Differences in Motor-Vehicle Crash Severity in California, Safety, 5(3), Article 48. doi:10.3390/safety5030048. Open access modelling demonstrates that older drivers tend to have higher proportions of serious and fatal injuries in crashes compared with other age groups
2. Nutbeam T, Kehoe A, Fenwick R, Smith J, Bouamra O, Wallis L, Stassen W. Do entrapment, injuries, outcomes and potential for self-extrication vary with age? A pre-specified analysis of the UK trauma registry (TARN). Scand J Trauma Resusc Emerg Med. 2022 Mar 5;30(1):14. doi: 10.1186/s13049-021-00989-w.
3. Nutbeam T, Weekes L, Heidari S, Fenwick R, Bouamra O, Smith J, Stassen W. Sex-disaggregated analysis of the injury patterns, outcome data and trapped status of major trauma patients injured in motor vehicle collisions: a prespecified analysis of the UK trauma registry (TARN). BMJ open. 2022 May 1;12(5):e061076.
4. Verma S, Wilson F, Wang H, Smith L, Tak HJ. Impact of Community Socioeconomic Characteristics on Emergency Medical Service Delays in Responding to Fatal Vehicle Crashes. AJPM Focus. 2023 Jun 20;2(4):100129. doi: 10.1016/j.focus.2023.100129. PMID: 37790947;

## Group 17: Cultural and Language Barriers in Emergency Response

*How do language barriers, cultural factors, and socioeconomic deprivation affect emergency response to road traffic collisions, and what interventions can address these inequalities?*

**Review**

Language, culture, and socioeconomic deprivation critically shape the emergency response to road traffic collisions, influencing both patient outcomes and engagement with emergency services.

Research consistently shows that individuals from ethnic minority backgrounds and lower socioeconomic groups are disproportionately affected by RTCs. Christie et al. (1) found that ethnic minority children in deprived UK neighbourhoods were more likely to be injured as car occupants, in part due to lower seatbelt use and substandard vehicle conditions. These disparities are compounded by language and cultural barriers that influence risk perception, help-seeking behaviour, and response to safety messaging.

Fatalistic cultural beliefs—where injury or death is perceived as inevitable or divinely preordained—can hinder engagement with preventive measures, such as seatbelt use or bystander first aid.

Inequities also extend to the provision of emergency medical services (2). Patients from deprived areas or minority backgrounds face disparities in EMS assessment, treatment decisions, and transport outcomes. These groups may experience delayed recognition of injury severity, reduced analgesia, or different conveyance pathways; factors that can worsen outcomes.

Interventions proposed to mitigate these issues include culturally sensitive public health campaigns, community paramedic schemes embedded within underserved areas, multilingual dispatch services, and enhanced EMS training on unconscious bias and cultural competence. However, much of the evidence originates outside the UK, and translation into policy remains inconsistent.

Gaps in the Literature

- There is a lack of prospective UK-based studies evaluating the impact of interventions (e.g. language lines, cultural competence training) on emergency outcomes in diverse communities.
- Limited data exists on the interaction between deprivation, language barriers, and EMS triage accuracy.
- Research often fails to disaggregate data by ethnicity, language proficiency, and cultural context, making it difficult to target responses effectively.

**Plain English Summary**

People from ethnic minority backgrounds or poorer communities often have worse outcomes after road traffic collisions. This is partly because they may not speak English well, may not trust emergency services, or may hold beliefs that discourage using safety measures. For example, some may not wear seatbelts or may delay calling for help. Studies show these groups may receive different care—such as slower assessments or less pain relief. Suggested solutions include training paramedics on cultural issues, using language lines, and working with local communities. However, most research is not UK-based, and more studies are needed to test what really works here.

**References**

1. Christie N, Kimberlee RH, Lyons R, Towner E, Ward H. Ethnicity, deprivation and road traffic injury risk: a survey of risk and road safety and implications for injury prevention. International journal of health promotion and education. 2008 Jan 1;46(4):133-8.
2. Bell F, Crabtree R, Wilson C, Miller E, Byrne R. Ambulance service recognition of health inequalities and activities for reduction: An evidence and gap map of the published literature. British Paramedic Journal. 2024 Jun 1;9(1):47-57.

## Group 18: Transport Decision-Making and Bypass Protocols

*What are the optimal transport decision-making protocols for road traffic collision patients, particularly in rural settings, and which non-clinical responders should be trained to initiate these decisions?*

**Review**

Transport decision-making protocols determine whether Helicopter Emergency Medical Services (HEMS) are dispatched to a road traffic collision (RTC) and the designation of hospital destination. These protocols are essential for identifying which patients will benefit from HEMS and/or a major trauma centre (MTC), whilst not encouraging over-/under-triage.

Two large European studies, in Sweden (1) and the Netherlands (2), found that distance from a trauma centre, rather than injury pattern, was the strongest determinant of prehospital transport, with each additional kilometre significantly increasing the odds of under-triage. This suggests ambulance crews factor distance into decisions even when guidelines do not. However, neither study accounted for secondary transfers, thus under-triage may have been overestimated.

Data from London’s Air Ambulance has been used to compare three HEMS dispatch criteria (3). The use of mechanism of injury criteria alone was found to be the most inaccurate, with only 58.7% of dispatches being appropriate, whilst paramedic telephone interrogation and on-scene ambulance crew requests were significantly more accurate. These data were from an urban environment, so extrapolating this to rural areas is difficult.

One U.S. study found emergency medical service provider judgement to be the most-used triage criterion in out-of-hospital trauma triage, with patients being identified for HEMS transfer using this criterion if they had more subtle injuries, comorbidities, or just “didn’t look right” (4). A recent study across four English trauma networks found clinician judgement to be more specific than theoretical tool performance when determining which trauma patients to transfer to a MTC (5).

An English study found that non-clinically trained dispatchers were more likely than clinically trained paramedic dispatchers to dispatch HEMS to cases requiring HEMS-specific critical interventions, without increasing response times (6).

**Gaps in the evidence**

More research on triage criteria is required specifically for rural RTC patients. Future research should examine whether a transport time limit exists for when transfer to a higher-level trauma centre benefits patients. Moreover, clearer distinction needs to be made between decisions regarding the modality of transport versus the destination. More research is required to determine which non-clinical responders should be enabled to make transport decisions for RTC patients.

**Plain English summary**

Protocols help decide when helicopters should be dispatched to a crash and which hospital a patient should go to. If optimal, these protocols ensure that patients get the most appropriate care. Despite not being included in protocols, distance from a trauma hospital determines where patients are transported to. ‘How a patient gets injured’ is not a useful decision-making tool. Instead, the judgement of ambulance crews is more useful at helping to make these decisions. Non-clinically trained staff make accurate decisions about sending helicopters to patients who need them, but more research is needed to decide which non-clinical people can make these decisions.

**References**

1. Fagerlind H, Harvey L, Candefjord S, Davidsson J, Brown J. Does injury pattern among major road trauma patients influence prehospital transport decisions regardless of the distance to the nearest trauma centre?–a retrospective study. Scandinavian journal of trauma, resuscitation and emergency medicine. 2019 Feb 13;27(1):18.
2. Waalwijk JF, Lokerman RD, van der Sluijs R, Fiddelers AA, den Hartog D, Leenen LP, Poeze M, van Heijl M, Pre-hospital Trauma Triage Research Collaborative (PTTRC). The influence of inter-hospital transfers on mortality in severely injured patients. European Journal of Trauma and Emergency Surgery. 2023 Feb;49(1):441-9.
3. Wilmer I, Chalk G, Davies GE, Weaver AE, Lockey DJ. Air ambulance tasking: mechanism of injury, telephone interrogation or ambulance crew assessment?. Emergency medicine journal. 2015 Oct 1;32(10):813-6.
4. Newgard CD, Kampp M, Nelson M, Holmes JF, Zive D, Rea T, Bulger EM, Liao M, Sherck J, Hsia RY, Wang NE. Deciphering the use and predictive value of “emergency medical services provider judgment” in out-of-hospital trauma triage: a multisite, mixed methods assessment. Journal of Trauma and Acute Care Surgery. 2012 May 1;72(5):1239-48.
5. Fuller G, Baird J, Keating S, Miller J, Pilbery R, Kean N, McKnee K, Turner J, Lecky F, Edwards A, Rosser A. The accuracy of prehospital triage decisions in English trauma networks–a case-cohort study. Scandinavian journal of trauma, resuscitation and emergency medicine. 2024 May 21;32(1):47.
6. Munro S, Joy M, de Coverly R, Salmon M, Williams J, Lyon RM. A novel method of non-clinical dispatch is associated with a higher rate of critical Helicopter Emergency Medical Service intervention. Scandinavian journal of trauma, resuscitation and emergency medicine. 2018 Sep 25;26(1):84.

## Group 19: Alternative Transport Methods and Outcomes

*What are the outcomes of alternative transport methods (police, private vehicles) compared to ambulance transport*

**Sub Question**

*What is the role and outcome of transport by non-ambulance vehicles (police or private car)? Research could investigate whether trauma patients who arrive via non-ambulance have different outcomes than similar patients brought by ambulance.*

**Review**

PubMed, Google Scholar and various renowned trauma journals were searched for relevant studies up to 28 Sep 2025 using the following terms: “police transport”, “private vehicle transport”, “ambulance”, “prehospital”, “fire service transport”, “scoop and run”, “bystander transport”, “self transport”, “trauma”, “outcome”. The literature that was found primarily consisted of observational registry and cohort studies (including large urban trauma registries) rather than randomized trials or large multi-centre cohort studies.

Several large analyses report that for penetrating trauma, private vehicle transport (PVT) or police “scoop-and-run” transport is associated with shorter prehospital times and similar or lower crude mortality compared with ambulances (1-3). Several studies specifically examining police transport report equivalent or non-inferior mortality for penetrating trauma when transported via police transport versus ambulances, while some show higher unadjusted mortality driven by greater injury severity in police-transported patients (1,3). More recent registry studies of police transport in blunt trauma suggest no significant difference in survival compared with ambulance transport, including large multi-centre analyses (4,5). This evidence remains mixed and findings are not uniform across settings.

Overall, current evidence suggests non-ambulance transport can reduce time-to-hospital and may be safe or beneficial in selected penetrating trauma, but high-quality prospective data across varied health systems and other emergency conditions are lacking. Evidence is limited in the UK.

**Plain English Summary**

Some studies show that people with penetrating injuries who arrive by police car or private vehicle reach hospital faster and may survive as well as, or better than, similar patients brought in by ambulance. Older and newer analyses support this finding, though differences often reduce after adjusting for how sick the patients were. For blunt trauma, recent studies suggest outcomes are similar whether patients are brought by police or ambulance. Overall, the evidence suggests that non-ambulance transport can shorten the journey to hospital and may be safe in some situations, but we still need stronger research across different countries and types of emergencies to be sure.

**References**

1. Band RA, Salhi RA, Holena DN, Powell E, Branas CC, Carr BG. Severity-adjusted mortality in trauma patients transported by police. Annals of emergency medicine. 2014 May 1;63(5):608-14.
2. Wandling MW, Nathens AB, Shapiro MB, Haut ER. Association of prehospital mode of transport with mortality in penetrating trauma: a trauma system–level assessment of private vehicle transportation vs ground emergency medical services. JAMA surgery. 2018 Feb 1;153(2):107-13.
3. Winter E, Hynes AM, Shultz K, Holena DN, Malhotra NR, Cannon JW. Association of police transport with survival among patients with penetrating trauma in Philadelphia, Pennsylvania. JAMA network open. 2021 Jan 4;4(1):e2034868-.
4. Rahhal R, Sakr P, Bachir R, El Sayed M. Outcomes of blunt trauma patients in police versus ground ambulance transport across US trauma centers. Acute Medicine & Surgery. 2025 Jan;12(1):e70061.
5. Kaufman EJ, Jacoby SF, Sharoky CE, Carr BG, Delgado MK, Reilly PM, Holena DN. Patient characteristics and temporal trends in police transport of blunt trauma patients: a multicenter retrospective cohort study. Prehospital emergency care. 2017 Nov 2;21(6):715-21.

## Group 20: Paediatric Considerations in Road Injury

How does paediatric “readiness” across the prehospital and hospital pathway—together with accurate trauma team activation (TTA) and systematic feedback of outcomes to clinicians—affect triage accuracy and 1-year outcomes for children injured in road traffic collisions (RTCs)?

**Review**

The grouped questions highlight three linked determinants of paediatric post-crash care: (1) recognition and TTA, especially when mechanism appears minor; (2) system readiness in EMS and receiving hospitals; and (3) routine feedback of patient outcomes to frontline clinicians. Together, these define whether the right child reaches the right team at the right time—and whether services learn and improve thereafter. ￼

Under-recognition of serious injury in children is a persistent risk when relying on mechanism or adult-derived physiology alone; children can compensate haemodynamically and appear well despite significant injury. Where TTA criteria are not child-specific (size/age-adjusted thresholds, injury patterns typical of paediatrics), under-triage increases, delaying definitive care. Embedding paediatric-specific triggers, coupled with active clinical override, is a rational system countermeasure.

“Paediatric-ready” EMS and hospitals encompass trained personnel, appropriate equipment and dosing, pathways to definitive care, and governance that monitors paediatric outcomes. Although direct, RTC-specific 1-year mortality evaluations are scarce, broader trauma literature associates higher paediatric readiness with lower mortality and fewer complications; EMS-readiness evidence is emerging but less mature. Within this pathway, feedback loops—timely, case-linked outcome information to crews—are a practical lever for quality improvement, associated with better adherence to guidelines and documentation in mixed-age EMS studies; paediatric-specific causal links to long-term outcomes remain limited.

Overall, the most plausible improvement strategy is bundle-based: paediatric-specific TTA criteria and education; verified EMS and hospital readiness standards; and regular, actionable feedback to clinicians. The evidence base for each element is stronger in general trauma than in RTC-specific paediatrics; nevertheless, the direction of effect and system logic justify prioritised implementation and prospective evaluation focused on under-triage rates, time-critical process measures, and 1-year functional outcomes. ￼

**Plain English Summary**

Children can look well after a crash even when badly hurt, so they are sometimes not sent straight to a specialist trauma team. Services that are “paediatric-ready” (trained staff, child-sized kit, clear pathways) and that give crews regular feedback on patient outcomes are more likely to get children rapidly to the right care. While evidence specific to road crashes is limited, the best approach combines child-specific triage rules, paediatric-ready EMS and hospitals, and routine feedback—then measures results over a year.

**References**

1. Newgard, C.D., Lin, A., Olson, L.M., Cook, J.N., Gausche-Hill, M., Kuppermann, N., Goldhaber-Fiebert, J.D., Malveau, S., Smith, M., Dai, M. and Nathens, A.B., 2021. Evaluation of emergency department pediatric readiness and outcomes among US trauma centers. JAMA pediatrics, 175(9), pp.947-956.
2. Newgard CD, Lin A, Goldhaber-Fiebert JD, Marin JR, Smith M, Cook JN, Mohr NM, Zonfrillo MR, Puapong D, Papa L, Cloutier RL. Association of emergency department pediatric readiness with mortality to 1 year among injured children treated at trauma centers. JAMA surgery. 2022 Apr 1;157(4):e217419-.
3. Larsson G, Larsson S, Strand V, Magnusson C, Andersson Hagiwara M. Pediatric trauma patients in Swedish ambulance services-a retrospective observational study of assessments, interventions, and clinical outcomes. Scandinavian journal of trauma, resuscitation and emergency medicine. 2024 Jun 5;32(1):51.

## Group 21: Psychological Impact on Bystanders and Responders

*What are the psychological impacts on bystanders who intervene in road traffic collisions, and how can support systems be designed to encourage intervention while minimising psychological harm?*

**Review**

Research into the psychological impact on bystanders who intervene in road traffic collisions (RTCs) is limited but provides several consistent themes. Systematic reviews show that members of the public often provide first aid, summon emergency services, or assist with traffic management. Their actions are shaped by cultural expectations, prior training, fear of legal consequences, and the timeliness of professional response (1). While these studies primarily describe functional roles, they also highlight that emotional strain and stress reactions are common but often overlooked.

Literature examining bystander experiences at and after motor vehicle accidents indicates that responses are influenced by perceptions of injury severity, moral responsibility, and fear of doing harm. Some individuals report pride and resilience, while others experience acute stress, intrusive memories, or longer-term psychological effects. Many express a desire for debriefing or follow-up support, yet formal systems to provide this are rarely available (2).

Quantitative evidence from adolescent populations demonstrates that witnesses of RTCs report significantly more trauma symptoms than unexposed peers, though fewer than direct victims. Symptoms include post-traumatic stress, fear, and depression, with outcomes strongly influenced by sex, coping style, and the availability of social support (3).

Insights from disaster and first responder research reinforce these findings. Exposure to traumatic events can lead to post-traumatic stress and burnout, but structured support, early reassurance, and accessible mental health services can be protective (4,5).

Overall, bystanders who intervene may experience a spectrum of outcomes, from empowerment to clinically significant distress. Evidence on tailored support systems is sparse, but extrapolation suggests that psychological first aid, peer or professional debriefing, and normalisation of emotional responses could encourage safe intervention while reducing harm. Future research should prioritise prospective, mixed-methods studies to evaluate prevalence, risk factors, and the effectiveness of targeted interventions.

**Plain English Summary**

People who stop to help after a road crash can be deeply affected by what they see and do. Some feel proud of helping, but others may struggle with distress, guilt, or even post-traumatic stress. Research shows that even witnesses who do not suffer injuries themselves can experience lasting emotional effects. At present, there are very few formal systems to support these bystanders. Simple steps such as follow-up contact, reassurance, and access to peer or professional support could help protect their well-being while encouraging safe intervention.opportunities, and recognition of their role. More research is needed on how best to protect and support these everyday helpers.

**References**

1. Heidari M, Aryankhesal A, Khorasani-Zavareh D. Laypeople roles at road traffic crash scenes: a systematic review. International journal of injury control and safety promotion. 2019 Jan 2;26(1):82-91.
2. Hall A, Wooton K, Hutton A. Bystander Experiences at and after a Motor Vehicle Accident: A review of the literature. Australasian Journal of Paramedicine. 2013 Jan;10:1-0.
3. Tierens M, Bal S, Crombez G, Loeys T, Antrop I, Deboutte D. Differences in posttraumatic stress reactions between witnesses and direct victims of motor vehicle accidents. Journal of Traumatic Stress. 2012 Jun;25(3):280-7.
4. Benedek DM, Fullerton C, Ursano RJ. First responders: mental health consequences of natural and human-made disasters for public health and public safety workers. Annual review of public health. 2007 Apr 21;28(1):55-68.
5. North CS, Pfefferbaum B. Mental health response to community disasters: a systematic review. Jama. 2013 Aug 7;310(5).

##

## Group 22: Defining Outcomes

How do we define “successful outcome” after road injury, and are we aligning research with what patients consider success?

**Review**

**Outcome measures**

Whilst short term outcomes such as survival to hospital discharge are easy to measure and report there is no consensus on how to assess long term health outcomes in injured patients. A systematic review by Hoffman et al (1) showed large numbers of outcome measures are available but they are used inconsistently between studies. The most frequently used outcome measure is the Medical Outcome Study Short Form Health Survey (SF-36) but this is only used a third of time. Other common measures are the European Quality of Life Questionnaire (EQ-5D), the Functional Independence Measure (FIM), and the Glasgow Outcome Scale (GOS). These measures capture only a small percentage of possible health impacts as defined by the WHO International Classification of Function, Disability and Health; the most comprehensive of which captures <5% of possible health outcomes.

**Patient perspective**

Some work exists examining what patients consider a successful outcome following trauma (2,3). Major themes are vulnerability, learning to manage, adaptation and adjustment and the subjective assessment of recovery. This ‘recovery of the self’ carries as much, if not more weight, than the objective degree of functional recovery or disability (recovery of the body). The degree of engagement with the recovery process was also associated with success, with high reported engagement correlated with more positive outcome assessments (2,3).

**Gaps in the evidence**

Despite the above-described work there is no universally accepted trauma outcome measure which has been developed with a major emphasis on the patient's subjective assessment of their own recovery. Most of the commonly used outcome measures have a focus on functional recovery with the EQ-5D having the most focus on subjective assessment.

There is also no agreed upon timeframe over which successful outcomes should be measured; whether this should be in the region of months or years following injury.

**Plain English Summary**

Many outcome measures exist for defining “successful outcomes” following trauma but none of these are universally applied in research. Further the more commonly used outcome measures have not been developed in line with what patients consider to be successful outcomes, which is largely around their subjective assessment and sense of self, rather than the degree of functional disability. More work is needed to define a suitable outcome measure and align research to consistently apply it in studies.

**References**

1. Hoffman K, Cole E, Playford ED, Grill E, Soberg HL, Brohi K. Health outcome after major trauma: what are we measuring?. PloS one. 2014 Jul 22;9(7):e103082.
2. Norris S, Graham L, Wilkinson L, Savory S, Robinson L. Patient perspectives of recovery following major musculoskeletal trauma: A systematic review and qualitative synthesis. Trauma. 2024 Jul;26(3):210-9.
3. Rosenberg G, Zion SR, Shearer E, Merrell SB, Abadilla N, Spain DA, Crum AJ, Weiser TG. What constitutes a ‘successful’recovery? Patient perceptions of the recovery process after a traumatic injury. Trauma Surgery & Acute Care Open. 2020 Feb 23;5(1).

## Group 23: Helmet Removal and Spinal Management

*What is the safety and effectiveness of lay person spinal management techniques (including helmet removal and log rolling) in road traffic collision casualties, and do structured instructions improve biomechanical safety*

**Review**

No literature directly examines the impact of bystander helmet removal. Advice for professionals to always remove a helmet has existed in the UK since at least 1994, primarily for airway assessment and maintenance (1). However, there is no such consensus for lay persons. Medical guidelines from around the world generally recommend that bystanders leave the helmet in place, with the exception of the German Red Cross who advocate helmet removal if the motorcyclist is unconscious (2). Multiple biomechanical studies demonstrate that even expert two-person removal of a motorcycle helmet can cause significant cervical spine movement, with one cadaveric study demonstrating that two out of ten unstable peg fractures were dislocated during helmet removal (3). However, these studies are limited to healthy volunteers and cadavers and there are no biomechanical data on patients with acute spinal injuries, where muscular spasm may provide some protection. Evidence is therefore lacking around both helmet removal in injured patients, and around the safety and effectiveness of lay person helmet removal.

Similarly, no published research evaluates bystander log rolling. The fundamental principle behind log rolling is to maintain spinal alignment to avoid secondary injury from an unstable spinal fracture. The optimal technique is generally regarded as requiring five personnel. Even when performed by trained professionals, it is noted as potentially dangerous due to the spinal motion generated. Multiple papers have demonstrated how log rolling can cause excessive movement (4) with the UK’s Faculty of Prehospital Care describing it as ‘detrimental’ (5). Whilst analysis of spinal movement when performed by professionals exists, there is no evidence reviewing log rolling performed by bystanders.

Finally, there is no published evidence on the optimal instructions to facilitate bystanders to achieve these manoeuvres safely. No widely used ambulance telephone triage system currently has a script directing bystanders to remove a motorcycle helmet or perform a log roll.

**Plain English Summary**

Casualties of road traffic collisions may sustain injuries to their spine. If a spinal injury is suspected, professionals will aim to protect the spine and prevent further damage by using careful handling when moving them. This includes a two-person technique to remove the helmet of an injured motorcyclist, and a five-person technique to roll a person onto their side if required. There is currently no evidence exploring whether bystanders can safely perform these procedures if required before professional help arrives, or what instructions could be given by an emergency call handler. This is a potential area for new research.

**References**

1. Branfoot T. Motorcyclists, full-face helmets and neck injuries: can you take the helmet off safely, and if so, how?. Emergency Medicine Journal. 1994 Jun 1;11(2):117-20.
2. Deutsches Rotes Kreuz (German Red Cross). *Helm abnehmen beim Verkehrsunfall (Remove helmet in traffic accident).* 2025. Available from: https://www.drk.de/hilfe-in-deutschland/erste-hilfe/verkehrsunfall/helm-abnehmen/. Accessed 2025 Sep 18.
3. Laun RA, Lignitz E, Haase N, Latta LL, Ekkernkamp A, Richter D. Mobility of unstable fractures of the odontoid during helmet removal. A biomechanical study. Der Unfallchirurg. 2002 Dec 1;105(12):1092-6.
4. Conrad BP, Del Rossi G, Horodyski MB, Prasarn ML, Alemi Y, Rechtine GR. Eliminating log rolling as a spine trauma order. Surgical neurology international. 2012 Jul 17;3(Suppl 3):S188.
5. Faculty of Pre‑Hospital Care, Royal College of Surgeons of Edinburgh. *Pre‑hospital Spinal Immobilisation: An Initial Consensus Statement.* 2024.

##

## Group 24: Predicting injury

*What are the most reliable clinical, mechanistic, and scene-level indicators for identifying occult life-threatening injuries (including internal bleeding and brain injury) in road traffic collision patients who present without obvious external signs of severe trauma?*

**Review**

Occult injuries—those not immediately detectable by visual, physiological, or simple anatomical assessment—pose a significant challenge in motor vehicle collisions (MVCs). These injuries often delay diagnosis and treatment, contributing to adverse outcomes if not triaged effectively. Several studies have attempted to characterise and quantify occult injuries through predictive scoring systems. Schoell et al. (1,2) (2015, 2017) introduced the *Occult Score* and *Transfer Score*, both designed to identify high-risk patients requiring trauma centre care. These tools integrate injury patterns, injury severity scores (ISS), and trauma system variables to estimate the likelihood that a patient has life-threatening injuries not immediately apparent.

High-occult risk injuries include abdominal lacerations, hemorrhage or hematoma, and head injuries classified as Abbreviated Injury Scale (AIS) 3–5 (1-3). These are particularly dangerous due to their internal nature and potential for rapid deterioration. Importantly, the presence of normal physiological parameters at the scene does not exclude serious injury. Shah & Alinier (4) emphasised the mechanism of injury (MOI)—such as high-speed impact, ejection, or rollover—as an independent predictor of occult trauma. Their findings reinforce the idea that visible signs may be misleading and that MOI should be central in triage algorithms, especially when dealing with “well-appearing” patients.

However, despite these advances, several gaps remain:

1. External Validation and Real-World Usability: The Occult and Transfer Scores have limited validation outside of retrospective data. Their effectiveness in prehospital settings and across diverse trauma systems remains largely untested.
2. Integration with Dispatch and Triage Systems: Current emergency call and dispatch systems often rely heavily on observable injury or patient-reported symptoms. Incorporating occult injury prediction tools into dispatch algorithms or decision support platforms is a crucial, unexplored frontier.
3. Lay Bystander and First Responder Application: There is a lack of training materials or simplified protocols enabling non-clinical personnel to consider MOI or other proxies for occult injury risk.
4. Global Health Context: Most studies originate in high-income countries. There is a critical need to test and adapt these tools in low-resource or rural environments, where diagnostic delays are more likely and trauma burden is higher.

Future research should focus on prospective validation, integration into dispatch and triage tools, and usability across varied health systems.

**Plain English Summary**

Occult injuries are serious internal injuries from car crashes that are not obvious at first glance. A person might look fine at the scene, but still have life-threatening damage—especially to the head or abdomen. Researchers have developed scoring tools, like the Occult Score and Transfer Score, to help identify these hidden injuries and decide who should go to a trauma centre. These tools use crash details, not just visible injuries or vital signs. However, more work is needed to test these tools in real-life settings, especially in rural or lower-income areas, and to help dispatchers and bystanders use this knowledge effectively.

**References**

1. Schoell SL, Doud AN, Weaver AA, Talton JW, Barnard RT, Winslow JE, Stitzel JD. Characterization of the occult nature of injury for frequently occurring motor vehicle crash injuries. Accident Analysis & Prevention. 2017 Jan 1;98:149-56.
2. Schoell SL, Doud AN, Weaver AA, Talton JW, Barnard RT, Winslow JE, Stitzel JD. Characterization of the occult nature of injury for frequently occurring motor vehicle crash injuries. Accid Anal Prev. 2017 Jan;98:149-156. doi: 10.1016/j.aap.2016.10.001. Epub 2016 Oct 7. PMID: 27723516.
3. Keisham S, Singh SB, Kamei R, Ph M. A study of fatal internal injuries without significant external injuries in road traffic accidents in Imphal from 2009-2014. Journal of Indian Academy of Forensic Medicine. 2015 Mar;37(1):16-8.
4. Shah Y, Alinier G. Collecting information from the scene of a motor vehicle collision: The mechanism of injury. Journal of Local and Global Health Science. 2015 Nov 16;2015(2):56.

## Group 25: Multi-Agency Co-Working Coordination and Training

*How can inter-agency coordination between police, fire, and medical services be optimised to reduce delays in extrication and improve patient outcomes at road traffic collisions, and what evidence-based models, training strategies, or protocols best support this integration?*

**Review**

Research into inter-agency coordination during road traffic collisions (RTCs) highlights both promising approaches and persistent challenges. Studies emphasise that effective extrication requires simultaneous contributions: police managing scene safety and traffic, fire services undertaking technical rescue, and medical teams focusing on patient stabilisation. However, evidence suggests inter-agency conflict and unclear command structures can delay patient transport (1).

Emerging strategies such as the EXIT (Extrication in Trauma) approach prioritise rapid patient-centred extrication, but uptake remains inconsistent, particularly between NHS and non-NHS providers, with slower, traditional methods still taught in some fire services (2). Simulation studies and cross-training models demonstrate potential benefits: when paramedics train alongside fire crews, smoother rescues and fewer errors are reported (3). International evidence supports the use of joint checklists and unified command protocols, though robust UK-specific evaluations remain limited. Furthermore, resource congestion at scenes—too many responders or vehicles—has been linked to inefficiency and reduced coordination (1).

Despite progress, major gaps persist. Few controlled studies directly compare different coordination models, and there is limited evidence linking protocol adoption to measurable patient outcomes such as survival or functional recovery. Most research evaluates process outcomes (e.g., extrication time) rather than clinical impact. Future work should explore barriers to implementation (cultural, logistical, and policy-related) and test whether integrated training, shared governance, or national frameworks improve both efficiency and patient outcomes.

**Plain English Summary**When someone is trapped in a vehicle after a crash, police, fire, and medical teams all need to work together quickly. Research shows that delays often happen when roles are unclear or when too many responders crowd the scene. Newer strategies, like the EXIT method and joint training between fire crews and paramedics, can make rescues faster and safer. However, these approaches are not used everywhere, and still lack strong evidence on how they affect patient survival. More real-world testing and better coordination policies are urgently required.

**References**

1. Nutbeam T, Fenwick R, Marritt I, Lee B, Staveley-Wadham L, Lang N, Johnson L, Mattock N, Ogilvie J, Foote E, Screech F. Optimising the care of the trapped patient following a motor vehicle collision: A UK-Based Delphi consensus study. Scandinavian Journal of Trauma, Resuscitation and Emergency Medicine. 2025 Aug 11;33(1):137.
2. Nutbeam T, Fenwick R, Smith JE, Dayson M, Carlin B, Wilson M, Wallis L, Stassen W. A Delphi study of rescue and clinical subject matter experts on the extrication of patients following a motor vehicle collision. Scandinavian journal of trauma, resuscitation and emergency medicine. 2022 Jun 20;30(1):41.
3. Kim JS, Benjamin ER, Kashani S, Eckstein M, Demetriades D. Bringing the operating room to the field: lessons learned from on-scene field amputations. European Journal of Trauma and Emergency Surgery. 2024 Dec;50(6):3243-9.

##

## Group 26: Rehabilitation

*How can post-collision rehabilitation systems be organised and supported—through early initiation, coordinated care, family engagement, and integration of social, legal, and financial support—to optimise functional recovery, return to work, and quality of life for road injury survivors across diverse settings?*

**Review**

Rehabilitation after road traffic injury is a complex, multifactorial process that extends far beyond the acute hospital episode. Evidence suggests that outcomes are influenced not only by the timing and intensity of therapy, but also by wider system organisation, family engagement, and social support. Early initiation of rehabilitation in intensive care or during the index hospital admission has been associated with improved functional outcomes and reduced disability, particularly in patients with traumatic brain injury and severe musculoskeletal injuries.

Integrated trauma systems and the presence of major trauma coordinators have been shown to improve continuity of care, reduce delays in referral, and enhance patient and family experiences by ensuring smoother transitions from acute to rehabilitation services. Families frequently describe the transition as challenging, with unmet needs in communication and coordination; structured support and clear information improve satisfaction and engagement.

External factors such as legal disputes, insurance processes, and financial stressors also significantly affect recovery. Studies in compensation systems show prolonged claims and adversarial processes are associated with poorer psychological outcomes and delayed return to work. Access to social work, financial counselling, or legal support within the rehabilitation pathway may mitigate these effects.

Evidence also highlights disparities: patients in rural areas often face delayed access to rehabilitation, while national and regional variations in service organisation contribute to differences in return-to-work outcomes. Scalable rehabilitation strategies, particularly for traumatic brain injury, remain a major challenge given the global burden.

**Conclusion**

Optimal post-collision rehabilitation requires early, coordinated, and holistic care that integrates clinical, family, social, and system-level support to maximise recovery and reduce inequities

**Plain English**

Recovery after a road crash is not just about hospital treatment. Starting rehabilitation early, even in intensive care, can improve recovery. Having trauma coordinators and well-joined-up services helps patients and families move more smoothly from hospital to rehabilitation. Families need clear communication and support during this stage. Stress from legal claims or financial problems can also slow recovery, so services that offer social or legal help may make a difference. Rehabilitation must be accessible in both urban and rural areas.

**References**

1. Andelic N, Sigurdardottir S, Schanke AK, Sandvik L, Sveen U, Roe C. Disability, physical health and mental health 1 year after traumatic brain injury. Disability and rehabilitation. 2010 Jan 1;32(13):1122-31.
2. Naess HL, Vikane E, Wehling EI, Skouen JS, Bell RF, Johnsen LG. Effect of early interdisciplinary rehabilitation for trauma patients: a systematic review. Archives of Rehabilitation Research and Clinical Translation. 2020 Dec 1;2(4):100070.
3. Gabbe BJ, Simpson PM, Cameron PA, Ponsford J, Lyons RA, Collie A, Fitzgerald M, Judson R, Teague WJ, Braaf S, Nunn A. Long-term health status and trajectories of seriously injured patients: a population-based longitudinal study. PLoS medicine. 2017 Jul 5;14(7):e1002322.

## Group 27: Physical risk to Bystanders and Responders

*What are the risks faced by rescuers and bystanders during roadside extrication and first response, and how can their physical and physiological safety be protected?*

**Review**

Shafaee(1) conducted a Delphi study to design a guide for call-centre staff. The guide has a dedicated domain on scene management and secondary injury prevention, such as turning ignitions off, removing cigarettes, wearing plastic gloves or bags on hands, and awareness of sharp objects.

In a literature review, Hall(2) found bystanders fear infection from contagion, as well as risks from sharp metal. Persons assisting at the scene of a road traffic collision (RTC) also experience physiological distress. Hall also states the driving ability of bystanders in the immediate period post-accident could be problematic due to the state of shock.

An observational study by Thierbach(3) reported on Emergency Medical Service retrospectively rating bystander actions, and found 18% of incidents involved RTCs, where 87% of bystanders made scenes visible to oncoming traffic.

Lavabre(4) describes the development of a tool, where simple safety precautions at RTCs were identified. The study validated for use by professional responders may have potential to inform a similar study for bystanders.

The AAA Foundation for Traffic Safety(5) highlights a gap in data on injuries and fatalities amongst bystanders.

The UK Government(6) reports killed and seriously injured (KSI), however bystanders are not identified by demographics.

A scoping review by Andrews(7) identified the following psychological consequences of being a lay rescuer: anxiety, distress, flashbacks, insomnia, guilt, a desire to learn their efforts were enough, and a desire to debrief after the experience.

Hall(2) found debriefing effective in the treatment of distress for bystanders at RTCs. However, whilst most responders at an RTC wished to talk with someone about their experience, organised debriefing was found to be rare or non-existent.

Sepahvand(8) conducted interviews with bystanders' and found fears and concerns included uncertainty of bystander protection laws and of their previous negative experiences.

**Plain English Summary**

Three themes were identified:

1. Reporting incidence of bystander injury is lacking, resulting in uncertainty in frequency, and size of the problem.
2. Dangers to bystanders are infrequently defined, however, fear of physical harm and negative psychological sequel are recognised.
3. Safety advice and training for bystanders.

A method of recording KSI, psychological, and non-fatal injuries, as well as near-misses to bystanders is recommended; and there may be opportunities to reduce incidents of physical and psychological injury to bystanders through training.

**References**

1. Shafaee H, Ostadtaghizadeh A, Khorasani-Zavareh D, Nematollahi S, Hirshon JM, Mirhaghi AH, Moradian MJ. Designing a guideline for emergency medical communication center staff to help road traffic crash patients: a Delphi study. Trauma monthly. 2021 Jan 1;26(1):41-51.
2. Hall A, Wooton K, Hutton A. Bystander Experiences at and after a Motor Vehicle Accident: A review of the literature. Australasian Journal of Paramedicine. 2013 Jan;10:1-0.
3. Thierbach AR, Pelinka LE, Reuter S, Mauritz W. Comparison of bystander trauma care for moderate versus severe injury. Resuscitation. 2004 Mar 1;60(3):271-7.
4. Lavabre K, Marjanovic N, Oriot D, Chenu M, Gransagne A, Gentilleau M, Moreau A, Contal P, Mimoz O, Drugeon B, Road Rescue Study Group Clouet Jean Gabriel 7 Van Esbroeck Tom 8 Guay Sébastien 9 Knoll Ruediger 10 Manesse Julien 11 Van Maurik Frans 12 Noyens Bart 13 Mouth Raphael 14 Rigollet Cédric 15. Creation and validation of a roadside rescue skills scale for training pre-hospital medical teams: the RoadRes-Q scale. Scandinavian journal of trauma, resuscitation and emergency medicine. 2025 Apr 3;33(1):56.
5. AAA Foundation for Traffic Safety. *Roadside Assistance Providers Fatally Struck by Vehicles at the Roadside: Incidence and Characteristics.* USA; 2025. Available from: https://aaafoundation.org/wp-content/uploads/2023/11/202401-AAAFTS-Roadside-Responder-Fatalities.pdf. Accessed 2025 Sep 5.
6. Department for Transport. *Reported Road Casualties in Great Britain, Provisional Estimates: 2024.* UK; 2025. Available from: https://www.gov.uk/government/statistics/reported-road-casualties-great-britain-provisional-results-2024/reported-road-casualties-in-great-britain-provisional-estimates-2024#casualties-by-road-user-type. Accessed 2025 Sep 9.
7. Andrews K, Paganini C, Sweeney D. Truck drivers are also lay rescuers: A scoping review. Australasian Journal of Disaster and Trauma Studies. 2024;28(1):87-95.
8. Sepahvand MJ, Nourozi K, Khankeh H, Mohammadi-Shahboulaghi F, Fallahi-Khoshknab M. Fears and concerns of bystanders to help people injured in traffic accidents: a qualitative descriptive study. Emergency medicine international. 2023;2023(1):1862802.

##

## Group 28: National Safety Reporting Systems

*What is the role of national safety reporting systems in improving post-crash outcomes?*

**Review**

National safety reporting systems are designed to capture information on serious incidents, near misses, and adverse outcomes within healthcare and emergency services. Their core role is to generate learning from error and system weaknesses, rather than to assign blame. In the context of trauma and road injury, such systems can provide a structured mechanism for identifying recurrent challenges in prehospital response, hospital care, or system coordination following crashes.

Examples include the National Reporting and Learning System (NRLS) in England (now integrated into the Learn from Patient Safety Events [LFPSE] service), as well as coronial or mortality review processes and confidential enquiries. These platforms allow aggregation of data on adverse outcomes, delays in care, equipment failures, or communication breakdowns. By analysing trends and disseminating learning, reporting systems can inform practice changes, guideline development, and targeted training.

However, limitations are well recognised. Reporting is often voluntary, leading to under-reporting, and many systems lack the granularity to address trauma-specific events. Feedback loops can be slow, and actionable recommendations may not reach frontline providers in a timely way. Furthermore, few systems are configured to link directly with trauma registries or road safety databases, restricting their utility for crash-specific improvement.

National safety reporting systems can contribute to post-crash outcome improvement by highlighting systemic weaknesses, sharing lessons, and promoting a safety culture. To maximise impact, integration with trauma registries, road traffic databases, and prehospital reporting structures is required, alongside timely feedback to those delivering care.

**Plain English Summary**

National reporting systems collect information on crashes and patient safety events to spot patterns, share lessons, and prevent repeat problems. In the UK, the NHS Learn from Patient Safety Events service records incidents in hospitals, while the Department for Transport’s STATS19 dataset tracks road crashes. These systems help identify risks, guide improvements, and support safer post-crash care, although they rely on accurate reporting and timely feedback to be most effective.

**References**

1. NHS England. *Learn from Patient Safety Events (LFPSE) service.* NHS England; 2025. Available from: https://www.england.nhs.uk/patient-safety/patient-safety-insight/learning-from-patient-safety-events/learn-from-patient-safety-events-service/. Accessed 2025 Sep 17.
2. NHS England. *Policy guidance on recording patient safety events and levels of harm.* NHS England; 2025. Available from: https://www.england.nhs.uk/long-read/policy-guidance-on-recording-patient-safety-events-and-levels-of-harm/. Accessed 2025 Sep 17.
3. Department for Transport. *Reported Road Casualties Great Britain: Road Accident Safety Data (STATS19).* Data.gov.uk; 2025. Available from: https://www.data.gov.uk/dataset/cb7ae6f0-4be6-4935-9277-47e5ce24a11f/road-accidents-safety-data. Accessed 2025 Sep 17.

## Group 29: Decision Support Tools

*What is the impact of structured decision-support tools (such as checklists, real-time guidance, or performance dashboards) on the quality, timeliness, and equity of prehospital care for trauma patients?*

**Review**

Decision-support tools such as checklists, cognitive aids, and real-time feedback systems have been proposed to improve the reliability and consistency of prehospital trauma care. The checklist approach has been shown in multiple areas of emergency and critical care to reduce omission of key steps and to standardise practice. In trauma, simulation and observational studies suggest that checklists may improve adherence to protocols and reduce time to critical interventions, though evidence specific to hypotensive trauma patients or road traffic collisions is limited.

Parallel developments include the use of technology-assisted guidance systems and performance dashboards, which provide real-time prompts, feedback on interventions, and post hoc benchmarking. These tools have shown promise in improving paramedic decision-making and adherence to clinical standards. Some EMS systems have reported improved documentation quality and greater protocol compliance when dashboards and electronic prompts are in place.

Importantly, one proposed benefit of such systems is the potential to improve equity of care. By reducing reliance on individual judgement and memory, decision-support tools may help standardise treatment across diverse patient groups and settings. However, evidence directly linking these tools to measurable reductions in disparities is lacking.

Conclusion: Checklists, electronic guidance, and dashboards appear to improve reliability and timeliness of prehospital care and may support equitable practice. Evidence is promising but remains indirect, with limited trauma- and road injury–specific outcome data. Further evaluation in real-world prehospital trauma systems is needed.

**Plain English Summary**

Tools such as checklists, electronic prompts, and performance dashboards can help paramedics give more consistent and timely care after a crash. Research shows these approaches make it less likely that key steps are missed and may speed up life-saving treatments. They might also reduce unfair differences in care between patients by standardising practice. However, most studies come from simulations or general emergency care, not specifically road injuries, so more research is needed to know their exact impact on trauma outcomes.

**References**

1. Droege H, Trentzsch H, Zech A, Prückner S, Imach S. A simulation-based randomized trial of ABCDE style cognitive aid for emergency medical services CHecklist In Prehospital Settings: the CHIPS-study. Scandinavian Journal of Trauma, Resuscitation and Emergency Medicine. 2023 Nov 17;31(1):81.
2. Chen Q, Qin Y, Jin Z, Zhao X, He J, Wu C, Tang B. Enhancing performance of the national field triage guidelines using machine learning: Development of a prehospital triage model to predict severe trauma. Journal of Medical Internet Research. 2024 Sep 30;26:e58740.
3. Kamgar Amaleh MH, Heydari S, Nazari P, Bakhshi F. Evaluating the effectiveness of the pre‑hospital trauma life support (PHTLS) program for the management of trauma patients in the pre-hospital emergency based on Kirkpatrick’s evaluation model. International Journal of Emergency Medicine. 2024 Jan 29;17(1):13.

## Group 30: What is the value of integrated data linkage across the trauma system for improving outcomes?

*What is the impact of developing and implementing nationally linked prehospital, trauma, and post-acute care data systems (including crash telematics, patient identifiers, and shared registries) on surveillance, quality improvement, research, and patient outcomes following road traffic injury?*

**Review**

The integration of data across the prehospital, hospital, and post-acute phases of trauma care has been repeatedly identified as a priority for improving outcomes after road traffic injury. At present, most trauma registries and EMS databases operate in silos, limiting the ability to track patients across the full continuum of care or to link system processes to long-term outcomes.

Evidence from trauma system evaluations demonstrates that linked registries allow for better benchmarking, quality improvement, and identification of best practices. National systems such as NEMSIS in the United States and TARN in the United Kingdom illustrate the potential of large-scale, standardised datasets for monitoring care and supporting research. However, neither currently provides seamless linkage across all phases, and most remain constrained by data protection, technical, and governance challenges.

Specific innovations have been proposed, including transmission of crash telematics to trauma centres, creation of uniform patient identifiers (e.g. trauma bands), and development of secure, interoperable platforms for medication and comorbidity data sharing. These concepts are supported by feasibility studies and stakeholder reports but lack robust outcome evaluations. Barriers include privacy regulation, cost, interoperability standards, and organisational alignment across multiple agencies.

The literature suggests that national data linkage enhances research capacity and policy development, with indirect evidence of improved system surveillance and safety reporting. Direct evidence demonstrating mortality or morbidity reduction attributable solely to data linkage is, however, limited.

Conclusion: Developing nationally linked trauma data systems has clear potential to improve surveillance, quality improvement, and research. The direct impact on patient outcomes remains less well established, but the rationale is strong, and system-level benefits are well supported.

**Plain English Summary**

At present, ambulance, hospital, and rehabilitation data are often kept separate, making it difficult to see the whole patient journey after a crash. Linking these data nationally could improve care by helping hospitals and services learn from patterns, share best practice, and support research. Although we do not yet have proof that this directly saves lives, the evidence suggests it strengthens system monitoring and helps identify ways to improve outcomes.

**References**

1. NHS England. *National Major Trauma Registry (NMTR).* NHS England Outcomes and Registries Programme; 2025. Available from: https://www.england.nhs.uk/outcomes-and-registries-programme/nmtr/. Accessed 2025 Sep 17.
2. UK Government. *Linking STATS19 and TARN: an initial feasibility study.* GOV.UK; 2025. Available from: https://www.gov.uk/government/statistics/linking-stats19-and-tarn-an-initial-feasibility-study/linking-stats19-and-tarn-an-initial-feasibility-study. Accessed 2025 Sep 17.
3. Williams N. Considering non-hospital data in clinical informatics use cases, a review of the National Emergency Medical Services Information System (NEMSIS). Informatics in medicine unlocked. 2022 Jan 1;35:101129.

## Group 31: What is the role and value of prehospital blood in the management of patients with road injury?

*What is the impact, feasibility, and implementation of prehospital blood and blood product transfusion (including whole blood, red cells, plasma, freeze-dried plasma, and innovative delivery methods such as drones) on survival and outcomes for trauma patients with haemorrhagic shock, particularly in rural or prolonged transport settings?*

**Review**

Haemorrhage is the leading preventable cause of death following trauma, and interest in prehospital blood product transfusion has grown significantly over the past decade. Multiple observational studies and a limited number of randomised trials have evaluated early use of red blood cells, plasma, and whole blood in the field. Findings suggest that prehospital transfusion is feasible and may be associated with improved haemostatic resuscitation and early survival, though evidence for long-term mortality benefit remains mixed.

Whole blood is gaining renewed attention for its balanced delivery of red cells, plasma, and platelets, with some military and civilian studies showing survival benefits. Freeze-dried plasma (lyophilised plasma), which does not require cold storage, has shown logistical advantages in austere and rural environments and has been safely used in European prehospital systems. However, clinical outcome data remain limited, and most evidence derives from feasibility and safety studies rather than powered outcome trials.

Paediatric data are sparse, though small series suggest that tranexamic acid (TXA) and plasma may be beneficial in haemorrhagic shock. Novel delivery models, such as drone transport of blood products, are under investigation but remain largely theoretical outside pilot studies.

Key barriers to implementation include cost, supply chain, cold-chain logistics, training, governance, and integration with hospital-based massive transfusion protocols. Facilitators include strong system leadership, collaboration between blood services and EMS, and the use of simplified products (e.g. freeze-dried plasma) that reduce logistic constraints.

Conclusion: Prehospital blood and product transfusion is promising, particularly for patients with haemorrhagic shock in rural or prolonged transport settings. Evidence supports feasibility and potential early survival benefit, but definitive proof of long-term outcome advantage is lacking. Implementation science is critical to guide safe, equitable adoption.

**Plain English Summary**

Severe bleeding is a major cause of death after crashes. Giving blood products before hospital arrival may help, especially when transport times are long. Whole blood, red cells, plasma, and freeze-dried plasma are all being studied, with early evidence suggesting possible survival benefits and good feasibility. Freeze-dried plasma is attractive because it does not need refrigeration, making it easier to carry in rural settings. However, strong proof that these treatments save lives long-term is still missing. Costs, logistics, and training remain key barriers to wider use.

**References**

1. Smith JE, Barnard EB, Brown-O’Sullivan C, Cardigan R, Davies J, Hawton A, Laing E, Lucas J, Lyon R, Perkins GD, Smith L. The SWiFT trial (Study of Whole Blood in Frontline Trauma)—the clinical and cost effectiveness of pre-hospital whole blood versus standard care in patients with life-threatening traumatic haemorrhage: study protocol for a multi-centre randomised controlled trial. Trials. 2023 Nov 14;24(1):725.
2. Crombie N, Doughty HA, Bishop JR, Desai A, Dixon EF, Hancox JM, Herbert MJ, Leech C, Lewis SJ, Nash MR, Naumann DN. Resuscitation with blood products in patients with trauma-related haemorrhagic shock receiving prehospital care (RePHILL): a multicentre, open-label, randomised, controlled, phase 3 trial. The Lancet Haematology. 2022 Apr 1;9(4):e250-61.
3. Pusateri AE, Moore EE, Moore HB, Le TD, Guyette FX, Chapman MP, Sauaia A, Ghasabyan A, Chandler J, McVaney K, Brown JB. Association of prehospital plasma transfusion with survival in trauma patients with hemorrhagic shock when transport times are longer than 20 minutes: a post hoc analysis of the PAMPer and COMBAT clinical trials. JAMA surgery. 2020 Feb 1;155(2):e195085-.

## Group 32: How does structured quality improvement influence patient outcomes in road injury care?

*What is the impact of implementing prehospital quality-improvement (QI) programmes—combining targeted feedback, micro-education, and regular electronic outcome feedback—on adherence to traumatic brain injury (TBI) guidelines, timeliness and quality of care, clinician experience, and patient outcomes?*

**Review**

Evidence from statewide and system-level programmes shows that structured QI—training plus ongoing audit-and-feedback—can improve adherence to prehospital TBI guidelines and may translate into better outcomes for the most severely injured patients. The Arizona EPIC project (education, protocol implementation, and linked registry evaluation) reported no overall survival effect across all TBI severities, but significantly improved adjusted survival in severe TBI subgroups, supporting the premise that guideline-focused QI can matter clinically when pathophysiological stakes are highest. ￼

Across EMS more broadly, audit-and-feedback interventions typically produce small-to-moderate improvements in professional practice; effects are larger when baseline adherence is low and feedback is specific, comparative, and actionable, particularly when combined with education or reminders (“micro-education”). This aligns with general Cochrane evidence on audit and feedback. ￼

Within EMS, recent mixed-methods and quantitative reviews show that feedback (including via electronic portals and dashboards) increases protocol adherence, improves documentation quality, and is perceived to support learning and wellbeing; emerging studies describe automated hospital-outcome feeds as feasible micro-learning tools. However, direct causal links to mortality or neurological outcomes remain limited outside focused programmes such as EPIC. ￼

Taken together, the literature supports a pragmatic model: implement QI bundles that pair brief, high-yield education with regular, timely, comparative feedback (ideally clinician-to-clinician and portal-enabled), and evaluate effects on process metrics (e.g., avoidance of hypoxia/hypotension, guideline adherence) and patient-centred outcomes (survival, neurological status). Systems should prioritise rapid feedback loops and linkage to hospital outcomes to maximise learning and behaviour change. ￼

**Conclusion**

Prehospital QI programmes with targeted feedback and micro-education reliably improve care processes and adherence; subgroup evidence suggests potential outcome benefits in severe TBI, but more trauma-specific, real-world outcome evaluations are needed.

**Plain English Summary**

Giving ambulance teams quick, useful feedback on their care—together with short, focused training—helps them follow head-injury guidelines more reliably. Reviews show that feedback and dashboards improve how care is delivered, especially when the messages are clear, frequent, and compare performance with peers. A large programme (EPIC) found better survival in the most severe head injuries. Overall, these approaches improve care processes; stronger evidence is still needed to prove broad effects on survival and long-term recovery

**References**

1. Spaite DW, Bobrow BJ, Keim SM, Barnhart B, Chikani V, Gaither JB, Sherrill D, Denninghoff KR, Mullins T, Adelson PD, Rice AD. Association of statewide implementation of the prehospital traumatic brain injury treatment guidelines with patient survival following traumatic brain injury: the excellence in prehospital injury care (EPIC) study. JAMA surgery. 2019 Jul 1;154(7):e191152-.
2. Ivers N, Jamtvedt G, Flottorp S, Young JM, Odgaard‐Jensen J, French SD, O'Brien MA, Johansen M, Grimshaw J, Oxman AD. Audit and feedback: effects on professional practice and healthcare outcomes. Cochrane database of systematic reviews. 2012(6).
3. Wilson, C., Janes, G., Lawton, R. and Benn, J., 2023. Types and effects of feedback for emergency ambulance staff: a systematic mixed studies review and meta-analysis. BMJ quality & safety, 32(10), pp.573-588.

## Group 33: What is the role of telemedicine in improving post-crash outcomes?

*What is the impact of telemedicine-supported medical direction for prehospital trauma care—particularly in rural or resource-limited settings—on triage decisions, patient outcomes, costs, and transfer efficiency?*

**Review**

Telemedicine in prehospital and trauma care is an expanding field, driven by advances in connectivity and digital platforms. Systems that allow EMS crews to access remote physician input in real time are increasingly feasible, particularly for rural regions where specialist expertise may be distant.

Evidence from observational studies and pilot programmes suggests several potential benefits. First, real-time telemedicine guidance can improve triage decisions, including more appropriate activation of aeromedical retrieval and earlier transfer to trauma centres. In rural settings, this may reduce secondary transfers, which are associated with worse outcomes and increased costs. Second, access to remote physician advice can support clinical decision-making, particularly for airway management, haemodynamic support, and determining thresholds for advanced interventions.

Economic analyses, though limited, indicate that telemedicine may reduce unnecessary transfers and associated costs, while enabling earlier identification of patients needing definitive trauma care. Studies in broader emergency contexts (e.g. stroke and cardiac telemedicine) show reductions in delays and improved patient outcomes, though trauma-specific outcome data are sparse.

Challenges include maintaining reliable connectivity in austere environments, ensuring data security, and integrating telemedicine into existing EMS protocols. Provider acceptance and adequate training are also critical facilitators of successful implementation.

**Conclusion**

Telemedicine-supported medical direction in prehospital trauma care appears feasible and may improve triage accuracy, reduce delayed transfers, and contain costs, especially in rural settings. However, high-quality comparative studies demonstrating direct patient outcome benefits are limited, and further evaluation in trauma-specific contexts is required.

**Plain English Summary**

Using telemedicine, ambulance teams can connect directly with hospital doctors while treating crash patients. This support may help paramedics make better triage choices, such as whether to call a helicopter or take a patient straight to a trauma centre. Early evidence suggests this approach could reduce unnecessary transfers, save money, and speed up care in rural areas. However, strong research proving that it improves survival or recovery in trauma is still limited, so more studies are needed before it can be widely adopted.

**References**

1. Schröder H, Beckers SK, Borgs C, Sommer A, Rossaint R, Grüßer L, Felzen M. Long-term effects of a prehospital telemedicine system on structural and process quality indicators of an emergency medical service. Scientific reports. 2024 Jan 3;14(1):310.
2. Stevanovic A, Beckers SK, Czaplik M, Bergrath S, Coburn M, Brokmann JC, Hilgers RD, Rossaint R, TEMS Collaboration Group Felzen Marc Hirsch Frederik Wolff Jürgen Lapp Nils Albrecht Lothar Koerentz Christof. Telemedical support for prehospital Emergency Medical Service (TEMS trial): study protocol for a randomized controlled trial. Trials. 2017 Jan 26;18(1):43.
3. Janerka C, Leslie GD, Mellan M, Arendts G. Prehospital telehealth for emergency care: A scoping review. Emergency Medicine Australasia. 2023 Aug;35(4):540-52.
4. Bergrath S, Brokmann JC, Beckers S, Felzen M, Czaplik M, Rossaint R. Implementation of a full-scale prehospital telemedicine system: evaluation of the process and systemic effects in a pre–post intervention study. BMJ open. 2021 Mar 1;11(3):e041942.

## Group 34: EMS System Delays

*How do EMS system delays affect patient outcomes after road injury?*

**Review**

Some studies have looked at how longer EMS response or on-scene times affect outcomes after MVCs and trauma more broadly. A large population-based US study by Byrne et al (1) found that counties with longer median EMS response times to MVCs (≥ 12 minutes vs <7) had significantly higher MVC mortality, even after adjusting for rurality, transport times, access to trauma centres etc. ￼

A study by Ashburn et al (2) analysed “scene and transport times” among trauma patients: prolonged scene time was associated with increased mortality in subgroups with hypotension or penetrating injury. ￼

Other work, e.g. Gonzalez et al (3), implies that in rural settings, increased EMS prehospital time correlates with worse outcomes after MVCs. ￼

However, the evidence is mixed. Many studies find associations but are observational, so confounding is likely (e.g. more severely injured or harder-to-access patients both have longer times and worse outcomes). There’s limited data isolating “APOT” specifically (i.e. time from arrival at scene to patient care or transport) in MVCs in UK settings. Also, transport times sometimes do not show clear relationships with mortality when other factors are controlled. ￼

Prolonged EMS times are associated with higher mortality after MVCs in multiple studies, particularly in rural settings or for severely injured patients. But causation is uncertain, and there is a lack of trauma- or MVC-specific data for UK systems. For APOT particularly, its independent contribution to worse outcomes isn’t clearly delineated.

**Plain English Summary**

The evidence suggests that longer delays before EMS care after crashes are linked to more deaths, especially in rural areas and among patients who are more severely injured. Some of this delay is in getting to the scene, and some is time spent there before moving to hospital. But it’s hard to tell how much of the bad outcomes are due to the delay itself versus the severity of the crash or other factors.

**References**

1. Byrne JP, Mann NC, Dai M, Mason SA, Karanicolas P, Rizoli S, Nathens AB. Association between emergency medical service response time and motor vehicle crash mortality in the United States. JAMA surgery. 2019 Apr 1;154(4):286-93.
2. Ashburn NP, Hendley NW, Angi RM, Starnes AB, Nelson RD, McGinnis HD, Winslow JE, Cline DM, Hiestand BC, Stopyra JP. Prehospital trauma scene and transport times for pediatric and adult patients. Western journal of emergency medicine. 2020 Feb 21;21(2):455.
3. Gonzalez RP, Cummings GR, Phelan HA, Mulekar MS, Rodning CB. Does increased emergency medical services prehospital time affect patient mortality in rural motor vehicle crashes? A statewide analysis. The American journal of surgery. 2009 Jan 1;197(1):30-4.

**SA1 - Out Of Scope**

##

## SA2: How many road traffic collisions are caused by a medical event and how are these identified and managed by prehospital responders?

**Review**

Road traffic collisions (RTCs) arise from multiple factors: driver-related, road-related, and vehicle-related. Among driver-related factors, pre-existing medical conditions and acute medical episodes may either contribute to, or directly cause, crashes. Medical events can impair driving through drowsiness and inattention, or cause sudden incapacitation due to seizures, syncope or arrhythmias (1).

**How many RTCs are caused by medical events?**

The proportion of RTCs caused by medical events varies depending on data source and crash severity studied. Analysis by the US Department of Transportation identified only 1.3% (n=49867/3794712) of crashes involving emergency medical service attendance as caused by medical emergencies, with higher incidence among older drivers (2). In an Australian cohort, Baldock and Raftery (1) reported that 9.2% (n=177/1939) of hospital admissions after crashes were directly precipitated by medical events, again commonly involving older adults or conditions such as seizures and syncope. By contrast, in Norway, Breen et al. (2) reported that up to 28% (n=84/301) of fatal collisions may have resulted from medical events, either involving drivers dying from natural causes or with conditions, predominantly cardiovascular, that carry a high risk of sudden incapacitation (3).

This variation likely reflects the difficulty in determining the contribution of medical events, as well as differences in inclusion criteria (all crashes vs. hospitalisations/fatalities), suggesting that medical-event RTCs are disproportionately associated with hospitalisation or death.

**How are these identified by prehospital responders?**

Prehospital responders may suspect a medical cause based on scene and patient findings. Typical features include single-vehicle crashes, departure from the roadway, and collision with fixed objects. Witnesses may describe erratic driving or sudden collapse (4). Collisions from medical events are also proportionally more likely in the morning (2).

**How are these managed by prehospital responders?**

Management prioritises both medical and trauma care. Following JRCALC guidance, responders perform a structured primary survey, assess mechanism of injury, and consider medical precipitants such as seizures or blackouts, initiating appropriate treatment alongside trauma protocols (5).

**Plain English Summary**

Some road traffic collisions are linked to sudden medical problems, such as seizures, fainting, or heart issues. Studies report varying figures, but roughly 1 in 100 crashes, 1 in 10 hospital admissions after a crash, and 1 in 4 fatal crashes involve a medical cause. This suggests that when medical problems trigger collisions, the outcomes are often serious. Prehospital responders may notice signs such as a single car leaving the road or hitting a fixed object, and should consider the crash mechanism to help identify possible medical causes.

**References**

1. Baldock MR, Raftery SJ. A cross-sectional study of the prevalence of medical conditions as contributors to road crashes in South Australia. Traffic Injury Prevention. 2025 Jan 2;26(1):24-32.
2. U.S. Department of Transportation, National Highway Traffic Safety Administration. *The contribution of medical conditions to passenger vehicle crashes.* *Ann Emerg Med.* 2010;55:563‑4. Available from: https://crashstats.nhtsa.dot.gov/Api/Public/ViewPublication/811219. Accessed 2025 Sep 8.
3. Breen JM, Naess PA, Gjerde H, Gaarder C, Stray-Pedersen A. The significance of preexisting medical conditions, alcohol/drug use and suicidal behavior for drivers in fatal motor vehicle crashes: a retrospective autopsy study. Forensic Science, Medicine and Pathology. 2018 Mar;14(1):4-17.
4. Neal A, Carne R, Odell M, Ballek D, D'Souza WJ, Cook MJ. Characteristics of motor vehicle crashes associated with seizure: car crash semiology. Neurology. 2018 Sep 18;91(12):e1102-11.
5. Joint Royal Colleges Ambulance Liaison Committee (JRCALC). *Clinical Practice Guidelines.* 2006. Available from: https://jrcalc.org.uk/wp-content/uploads/2017/12/JRCALC_clinical_guidelines_2006.pdf. Accessed 2025 Sep 8.

## SA3: In patients with major trauma requiring airway intervention (Population), how does time to definitive airway management during nighttime hours (Intervention) compare to daytime hours (Comparator) in the period following the establishment of regional trauma networks (Outcome)?

**Review**

Electronic databases such as Pubmed/Medline, Google Scholar, and Scopus were used to identify relevant articles using search terms ‘pre-hospital’, ‘RSI’, ‘anaesthesia’, ‘airway’, ‘time’, ‘day’ and ‘night’. There was no published evidence directly addressing the question. 13 articles considered pre-hospital interventions including airway management. Out of these, three measured time to definitive airway, and four used data from UK major trauma networks.

A prospective observational study of six HEMS teams across four Nordic countries over one year measured the successful intubation in pre-hospital patients: time from scene arrival to intubation, attempt number, and complications (1). It included patients intubated with and without rapid sequence intubation (RSI). Time taken to scene was not considered, potentially influenced by time of day, and traumatic or medical aetiology was not specified.

NICE guidance of 45-minutes to RSI was examined in a retrospective cohort study of HEMS teams in South-East England delivering RSI to major trauma patients, where pre-hospital RSI was compared with Emergency Department RSI (2). Primary outcome was time from emergency call to RSI, and a secondary outcome was time from arrival (at scene or ED) to RSI. Patients received RSI quicker pre-hospitally, but factors influencing time from call to scene or call to RSI, such as time of day, were not discussed.

An observational cohort study of 20 UK HEMS units showed 1755 emergency anaesthetics undertaken over one year, 25% of which took place within 45 minutes of the emergency call (3). 1176 (67%) were undertaken in daylight hours, but time taken to RSI in daytime versus nighttime is not directly compared. Intubations not requiring drug administration were excluded (neuromuscular blocking agent administration time was used as a proxy for advanced airway management). Patients with non-traumatic aetiology were excluded. The study concluded dispatch delays were associated with increased time to pre-hospital emergency anaesthesia.

While time taken to establish a definitive airway pre-hospitally is considered in these studies, focus on major trauma patients, and the impact of time of day, is lacking. Dispatch delays, availability of helicopters at night, human and environmental factors could all be influenced by time of day, and to what extent remains uncertain.

**Plain English Summary**

When people sustain severe injuries, they sometimes need medical teams to place a tube down their throat to help them breathe before they are taken to hospital. While data has been collected on how long it takes medical teams to place these tubes, it is unclear so far whether placing them during nighttime hours has different results to placing them during the day. By measuring this, changes could be made to how medical teams operate to improve their care of trauma patients at different times of the day or night.

**References**

1. Gellerfors M, Fevang E, Bäckman A, Krüger A, Mikkelsen S, Nurmi J, Rognås L, Sandström E, Skallsjö G, Svensén C, Gryth D. Pre-hospital advanced airway management by anaesthetist and nurse anaesthetist critical care teams: a prospective observational study of 2028 pre-hospital tracheal intubations. British journal of anaesthesia. 2018 May 1;120(5):1103-9.
2. Heritage D, Griggs J, Barrett J, Clarke S, Carroll R, Lyon R, Bootland D. Helicopter emergency medical services demonstrate reduced time to emergency anaesthesia in an undifferentiated trauma population: a retrospective observational analysis across three major trauma networks. Scandinavian Journal of Trauma, Resuscitation and Emergency Medicine. 2024 Dec 27;32(1):138.
3. Turner J, Bourn S, Raitt J, Ley E, O'Meara M. Pre-hospital emergency anaesthesia in the United Kingdom: an observational cohort study. British Journal of Anaesthesia. 2020 May 1;124(5):579-84.

## SA4: What is the effect of scene time in rural road injuries on mortality, stratified by injury severity and injury type?

**Review**

The relationship between scene time and mortality in rural road traffic collisions (RTCs) is hugely complex and influenced by many distinct factors. For example, access to definitive care, injury severity, and injury type. Evidence currently shows that longer scene times are associated with increased mortality, but this is influenced by the need for advanced prehospital interventions. When looking at scene time this can be divided into response time and time on scene of a RTC.

Literature from the United States found that longer emergency medical service (EMS) response times are linked with higher mortality in RTCs in both rural and urban environments (1). When delving deeper into scene time specifically, research has found that rural crash victims experience significantly longer on scene times and mortality. However, when further stratified into injury severity score (ISS), mortality could not be predicted solely on scene time (2). A systematic review evaluating mortality in major traumatic injuries found that scene times exceeding 10 minutes showed a significant association with mortality. This effect was most pronounced in patients with severe or penetrating injuries (3). Although most RTC mortalities are due to blunt force trauma rather than penetrating injuries, similar time sensitive physiology and need for emergency care applies. (4) A large national study from Norway compared prehospital time and mortality in patients in both urban and rural environments. It found that prolonged on scene time was associated with higher rates of mortality, but rural location itself was not associated with increased mortality. (5) Overall, the literature shows that increasing pre-hospital times are associated with higher mortality in traumatic injuries, with rural trauma and RTCs being more susceptible to this. This is due to several factors including time to and on scene, interventions needed and time to a major trauma unit.

**Plain English Summary**

In road accidents the longer patients spend outside the hospital the higher their chance of death is. This is true of road accidents in both urban and rural locations. There are many factors that influence the amount of time out of hospital. These include the time it takes to get to the scene, time on scene, medical procedures that the patient needs and time it takes to get to a suitable hospital. Rural areas are more susceptible to these challenges.

**References**

1. Byrne JP, Mann NC, Dai M, Mason SA, Karanicolas P, Rizoli S, Nathens AB. Association between emergency medical service response time and motor vehicle crash mortality in the United States. JAMA surgery. 2019 Apr 1;154(4):286-93.
2. Gonzalez RP, Cummings GR, Phelan HA, Mulekar MS, Rodning CB. Does increased emergency medical services prehospital time affect patient mortality in rural motor vehicle crashes? A statewide analysis. The American journal of surgery. 2009 Jan 1;197(1):30-4.

## SA5: What are people dying from after road injury - how much of this is reversible and in what time scale?

**Review**

Among patients sustaining road traffic injuries, head injuries are the leading cause of death, reported in 44.7% to 88.8% of fatalities across studies. Haemorrhagic shock is the second most frequent cause, implicated in up to 70% of deaths. Chest, abdominal, pelvic, spinal, and extremity injuries contribute less commonly but remain important drivers of mortality.

The proportion of potentially preventable deaths varies substantially across the literature, reflecting differences in methodology and setting. Studies using statistical or model-based approaches typically report 4–17% preventable deaths, whereas expert panel reviews or autopsy-based assessments identify much higher figures, up to 68–80%. Across datasets, delays in diagnosis, prehospital intervention, and definitive care are repeatedly identified as major contributors to preventable mortality.

Timing of interventions is critical. Between 16% and 32% of deaths occur within the first hour after injury, underscoring the concept of the “golden hour” in trauma care. Within this period, the first 15 minutes appear especially crucial, with studies showing that rapid action—including haemorrhage control, airway management, and timely transfer to definitive care—significantly reduces mortality.

The global literature also highlights disparities between high-income and low- and middle-income countries (LMICs). In LMICs, where access to advanced prehospital and hospital care is limited, preventable mortality is often higher, and time-critical interventions are less likely to be delivered promptly.

**Plain English Summary**

Road traffic injuries cause many deaths worldwide, mostly from head injuries and severe bleeding. Across studies, up to 80% of these deaths could be prevented if people received faster, better care. Time is critical. Around one in three deaths happen in the first hour, and treatment within the first 15 minutes offers the best chance of survival. Quick actions such as stopping bleeding, managing the airway, and getting patients to hospital rapidly can save lives. In lower-income countries, preventable deaths are more common due to limited access to advanced trauma care and delays in reaching treatment. Overall, improving outcomes after serious crashes requires faster emergency responses, better prehospital care, and rapid transfer to specialist centres. Integrating these steps into coordinated trauma systems can help ensure people get life-saving treatment when they need it most.

**References**

1. Razzak JA, Bhatti J, Wright K, Nyirenda M, Tahir MR, Hyder AA. Improvement in trauma care for road traffic injuries: an assessment of the effect on mortality in low-income and middle-income countries. Lancet. 2022 Jul 23;400(10348):329-336. doi: 10.1016/S0140-6736(22)00887-X. Epub 2022 Jun 30. PMID: 35779549.
2. Ryan M, Stella J, Chiu H, Ragg M. Injury patterns and preventability in prehospital motor vehicle crash fatalities in Victoria. Emerg Med Australas. 2004 Aug;16(4):274-9. doi: 10.1111/j.1742-6723.2004.00622.x. PMID: 15283713.
3. Sahdev P, Lacqua MJ, Singh B, Dogra TD. Road traffic fatalities in Delhi: causes, injury patterns, and incidence of preventable deaths. Accident Analysis & Prevention. 1994 Jun 1;26(3):377-84.

## SA6: How often do UK trauma audit systems capture long-term psychological sequelae?

**Review**

Major trauma can result in long-term psychological sequelae such as post-traumatic stress disorder (PTSD), depression, anxiety, and reduced quality of life. Capturing these outcomes is important for understanding the full impact of trauma and for guiding service improvement. In the United Kingdom, two main audit systems are relevant: the Trauma Audit Research Network (TARN) and the more recently introduced National Major Trauma Registry (NMTR).

TARN’s dataset has historically focused on acute care and discharge outcomes, including mortality, discharge destination, critical care admission, and Glasgow Outcome Scale at discharge or 30 days. It does not include mandated measures of psychological health or long-term quality of life. The NMTR, launched under NHS England in 2024, continues this emphasis on the acute pathway, with no current requirement for the routine capture of psychological outcomes.

Although individual research studies in the UK have examined psychological recovery after trauma, these efforts are not integrated into national audit systems. NICE guidance recognises this as a gap and calls for the inclusion of functional and psychosocial outcomes in future registry development. At present, however, UK trauma audit systems do not routinely or systematically collect psychological outcome data, leaving an important aspect of recovery underrepresented.

**Plain English Summary**

Major trauma can affect mental health as well as the body. UK trauma audits, such as TARN and the new National Major Trauma Registry, collect short-term hospital data but do not routinely track long-term psychological recovery. This leaves an important gap in understanding patients’ full recovery after injury.

**References**

1. Trauma Audit and Research Network (TARN). *ISB 1606: Trauma Audit and Research Network Standard Dataset.* NHS Digital; 2014. Available from: https://digital.nhs.uk/data-and-information/information-standards/governance/latest-activity/standards-and-collections/isb-1606-trauma-audit-and-research-network-standard-dataset.
2. NHS England. *National Major Trauma Registry.* Outcomes and Registries Programme; 2024. Available from: https://www.england.nhs.uk/outcomes-and-registries-programme/nmtr/.
3. National Institute for Health and Care Excellence (NICE). *Major trauma: assessment and initial management (NICE guideline NG39).* 2016. Available from: https://www.nice.org.uk/guidance/ng39.

## SA7: What’s the best pain relief in the early treatment and transfer

**Review**

The question of what constitutes the “best” analgesic for early treatment and transfer after road traffic injury does not have a single answer. Evidence from both emergency medicine and anaesthesia highlights that the definition of “best” varies considerably between patients, environments, and clinical circumstances. Relevant factors include patient comorbidities, age, physiology, level of consciousness, injury type, and anticipated trajectory. Practical considerations also play a major role, such as provider training and permissions, the range of drugs available, routes of administration, anticipated transport time, monitoring capability, and safety profile in austere or dynamic settings.

The anaesthesia literature discusses the “ideal analgesic,” describing characteristics such as rapid onset, predictable titration, short duration, minimal haemodynamic or respiratory compromise, and ease of administration. However, no single drug achieves this profile. In prehospital care, options such as opioids, ketamine, nitrous oxide, and regional techniques all carry different advantages and limitations depending on context.

Given this variability, it is unlikely that a single trial could define one analgesic as superior in all situations. The more appropriate approach is a pragmatic, patient-centred selection informed by clinical judgement and available resources.

**Plain English Summary**

The question of the “best” analgesic in early trauma care cannot be answered by a single study or guideline, as suitability varies with patient, provider, drug, and context. The anaesthesia concept of the “ideal analgesic” is useful, but unattainable in practice. Prehospital options all have context-specific benefits and drawbacks. A pragmatic, patient-centred approach is therefore needed, making this question unsuitable for prioritisation within a PSP framework.

**References**

1. Galinski M, Ruscev M, Gonzalez G, Kavas J, Ameur L, Biens D, Lapostolle F, Adnet F. Prevalence and management of acute pain in prehospital emergency medicine. Prehospital Emergency Care. 2010 May 1;14(3):334-9.
2. Abebe Y, Hetmann F, Sumera K, Holland M, Staff T. The effectiveness and safety of paediatric prehospital pain management: a systematic review. Scandinavian Journal of Trauma, Resuscitation and Emergency Medicine. 2021 Dec 11;29(1):170.
3. Bradford JM, Cardenas TC, Edwards A, Norman T, Teixeira PG, DuBose J, Kempema J, Ali S, Brown CV. Racial and ethnic disparity in prehospital pain management for trauma patients. Journal of the American College of Surgeons. 2023 Mar 1;236(3):461-7.

## SA8: Does integrating medical personnel into the extrication process improve outcomes?

**Review**

The question of whether integrating medical personnel directly into the vehicle extrication process improves outcomes has not been empirically answered. No comparative study was identified that contrasts rescue-only extrication with integrated medical–rescue approaches.

Available evidence instead highlights the risks associated with entrapment and extrication delay. Registry and observational studies consistently show that entrapped patients have more severe injuries and poorer outcomes, with extrication often exceeding thirty minutes. Although predictive tools exist to estimate extrication duration, outcome studies linking integration of medical staff during the rescue phase remain absent.

Consensus guidance and doctrine, developed through expert and Delphi processes, promote a patient-centred approach to extrication. These emphasise early medical assessment, life-saving interventions such as haemorrhage control and airway management, and the use of minimally invasive or self-extrication where possible. Such frameworks imply value in medical–rescue collaboration, but they provide no direct outcome data.

Indirect evidence from physician-staffed prehospital trauma systems suggests medical involvement at scene can improve survival in severe trauma. However, these findings are not extrication-specific and may be confounded by system design and case-mix.

Overall, extrication delays clearly worsen outcomes, but whether integration of medical personnel improves survival remains unanswered, representing an important research gap

**Plain English Summary**

There is no research showing whether having medical staff work directly alongside rescuers during vehicle extrication improves survival. We know that being trapped and long delays are linked to worse outcomes, and expert guidance supports closer teamwork, but there is no clear evidence that integration itself changes outcomes.

**References**

1. Nutbeam T, Fenwick R, Hobson C, Holland V, Palmer M. The stages of extrication: a prospective study. Emergency Medicine Journal. 2014 Dec 1;31(12):1006-8.
2. Nutbeam T, Fenwick R, Smith JE, Dayson M, Carlin B, Wilson M, Wallis L, Stassen W. A Delphi study of rescue and clinical subject matter experts on the extrication of patients following a motor vehicle collision. Scandinavian journal of trauma, resuscitation and emergency medicine. 2022 Jun 20;30(1):41.
3. Nutbeam T, Fenwick R, Smith J, Bouamra O, Wallis L, Stassen W. A comparison of the demographics, injury patterns and outcome data for patients injured in motor vehicle collisions who are trapped compared to those patients who are not trapped. Scandinavian journal of trauma, resuscitation and emergency medicine. 2021 Jan 14;29(1):17.

##

## SA9: What is the impact of immediate pain relief on trauma patient outcomes? Pain from fractures or injuries can induce shock (via stress responses) and traumatise the patient psychologically. Unanswered aspects: Does early aggressive pain control (I) versus minimal pain management until hospital (C) make a difference in outcomes like reduction in anxiety/PTSD, or even physiologic benefits (lower catecholamines leading to less bleeding)? Are there ideal analgesic agents in the field?

**Review**

The impact of immediate pain relief on trauma patient outcomes remains incompletely understood. Acute pain following fractures or major injuries activates stress responses, with catecholamine release contributing to tachycardia, hypertension, and potential exacerbation of bleeding. Pain is also recognised as a psychological stressor that may increase the risk of long-term sequelae such as anxiety, depression, or post-traumatic stress disorder (PTSD).

Despite this theoretical rationale, few studies directly compare early aggressive pain control with minimal or delayed analgesia in the prehospital trauma setting. Observational data show that prehospital pain remains undertreated and that inadequate analgesia is associated with poorer patient experience and satisfaction. Some registry-based studies suggest that effective prehospital analgesia is not associated with harm and may reduce physiological stress, but definitive evidence linking it to improved survival, haemorrhage control, or long-term psychological outcomes is lacking.

The concept of the “ideal analgesic” from the anaesthesia literature—rapid onset, reliable titration, minimal haemodynamic instability, ease of administration—has been applied to the prehospital setting, but no single drug fulfils all criteria. Opioids, ketamine, nitrous oxide, and regional techniques each have context-specific advantages and limitations.

In summary, immediate pain relief is intuitively beneficial and strongly supported for humane and ethical reasons, but robust evidence for its impact on physiological or psychological outcomes in trauma patients is sparse. This question may not lend itself to traditional trial methodology and instead highlights an evidence gap suitable for observational or mixed-methods research.

**Plain English Summary**

There is little research showing whether giving strong pain relief immediately after an accident improves trauma patient outcomes. Severe pain can trigger stress responses that may worsen bleeding and can also cause lasting psychological harm such as anxiety or PTSD. While it seems logical that early, effective pain relief would help, studies have not proven benefits such as better survival or reduced long-term problems. Different drugs each have pros and cons, and none is perfect in all situations. Providing timely pain relief remains important for patient comfort and dignity, even though its wider outcome benefits are not fully known.

**References**

1. Galinski M, Ruscev M, Gonzalez G, Kavas J, Ameur L, Biens D, Lapostolle F, Adnet F. Prevalence and management of acute pain in prehospital emergency medicine. Prehospital Emergency Care. 2010 May 1;14(3):334-9.
2. Abebe Y, Hetmann F, Sumera K, Holland M, Staff T. The effectiveness and safety of paediatric prehospital pain management: a systematic review. Scandinavian Journal of Trauma, Resuscitation and Emergency Medicine. 2021 Dec 11;29(1):170.
3. Bradford JM, Cardenas TC, Edwards A, Norman T, Teixeira PG, DuBose J, Kempema J, Ali S, Brown CV. Racial and ethnic disparity in prehospital pain management for trauma patients. Journal of the American College of Surgeons. 2023 Mar 1;236(3):461-7.

##

## SA10: How long are air ambulances waiting before they can land on roads at the scene of RTCs? What are the barriers to early road closure at RTCs, barriers which result in delayed ability for air ambulances to land? What solutions would overcome this?

**Review**

A review of the available literature reveals that the specific question of how long air ambulances must wait before landing on roads at the scene of road traffic collisions (RTCs) has not been addressed empirically. No study to date has quantified the delay attributable to road closure procedures or the time aircraft spend orbiting overhead awaiting access. The closest empirical proxies include analyses of helicopter emergency medical services (HEMS) on-scene times, where landing-site distance and clinical interventions have been shown to influence overall mission duration. These findings, while relevant, do not directly measure the delays imposed by road closure.

Alongside these limited data, a body of doctrine and operational guidance provides insights into the processes and barriers. National and international guidance (e.g. JESIP, CTAC, Civil Aviation Authority standards, National Police Chiefs’ Council advice) emphasises the legal requirement to establish cordons, stop traffic in both directions, and mitigate hazards such as overhead wires or foreign object debris before a carriageway can be used as a landing zone. These requirements highlight structural, system-level barriers—principally legal, safety, and coordination issues—that are consistently acknowledged across sources. Such barriers are well known, widely explained, and form the operational context within which HEMS teams must operate.

**Plain English Summary**

There is no research showing exactly how long air ambulances wait before they can land on roads after a crash. Studies do show that landing further away adds delay, but they do not measure waiting for road closures. Official guidance makes clear that traffic must be stopped and hazards checked before a road can be used. These safety and coordination rules are well recognised barriers and explain why delays can occur, even if the actual waiting times are not reported in the scientific literature.

**References**

1. Nakstad AR, Strand T, Sandberg M. Landing sites and intubation may influence helicopter emergency medical services on-scene time. The Journal of Emergency Medicine. 2011 Jun 1;40(6):651-7.
2. Fuchs A, Huber M, Riva T, Becker S, Albrecht R, Greif R, Pietsch U. Factors influencing on-scene time in a physician-staffed helicopter emergency medical service (HEMS): a retrospective observational study. Scandinavian Journal of Trauma, Resuscitation and Emergency Medicine. 2023 Apr 14;31(1):20.
3. JESIP. *Combined Tactical Air Cell (CTAC) Guidance (incl. HELP and landing‑site coordination).* 2019. Available from: https://www.jesip.org.uk/wp-content/uploads/2024/02/Combined_Tactical_Air_Cell_CTAC_.pdf.

## SA11: What is the optimum time to pre-alert Emergency Departments of intended/inbound patients from RTCs, and who should provide these pre-alerts?

**Review**

The optimum timing of pre-alerts to Emergency Departments (EDs) for patients involved in road traffic collisions (RTCs), and the appropriate personnel to provide these alerts, is not defined in the published literature. No comparative studies were identified that assess early versus later pre-alerts in relation to patient outcomes or ED preparedness.

Guidelines from prehospital and emergency care systems emphasise the principle of early communication to allow mobilisation of trauma teams, preparation of resources, and activation of operating theatres or imaging. The Advanced Trauma Life Support (ATLS) framework, national trauma networks, and prehospital emergency medicine (PHEM) standards all recommend pre-alerting as soon as a major trauma patient is recognised, often guided by triage tools. However, these documents do not specify an exact “optimum time.”

Regarding who should provide pre-alerts, standard practice is that the lead prehospital clinician—commonly a paramedic or prehospital physician—communicates directly with the receiving ED. Some systems support relay via a control room, but direct clinician-to-clinician communication is generally preferred to ensure accuracy and effective handover.

In summary, while early pre-alert is universally recommended to optimise preparedness, no evidence defines the precise timing, and practices vary between systems. The key determinant is timely recognition of major trauma and clear communication from the responsible prehospital clinician.

**Plain English Summary**

There is no clear research on the best time to call ahead to the Emergency Department after a road traffic collision. Current guidance says pre-alerts should be made as soon as serious injury is recognised so the trauma team can prepare. Usually, the lead paramedic or doctor makes this call directly.

**References**

1. Cole E, Lecky F, West A, Smith N, Brohi K, Davenport R, ELoTS Study Collaborators. The impact of a pan-regional inclusive trauma system on quality of care. Annals of Surgery. 2016 Jul 1;264(1):188-94.
2. National Institute for Health and Care Excellence (NICE). Major trauma: service delivery. NICE guideline NG40. 2016.
3. ATLS Subcommittee; American College of Surgeons’ Committee on Trauma; International ATLS working group. Advanced trauma life support (ATLS®): the ninth edition.Journal of Trauma and Acute Care Surgery. 2013 May;74(5):1363‑6.

## SA12: What clinical signs and physiological parameters are bystanders (lay-persons and non-clinical responders) able to accurately and reliably detect/measure in the context of road injury?

**Review**

PUBMED was searched on the 24/09/25. Search parameters were refined through an iterative cycle using keywords from the question. Final search criteria included Trauma AND (Bystander OR Layperson) AND (Measurement OR Assessment) with MESH subheadings, and English language restrictions. Google Scholar was searched using these same keywords. A total of 418 articles were identified. 10 articles were selected for full text review. Four articles were determined to be of relevance to the question.

There is a wealth of literature exploring bystander first-aid, including training and provision. A systematic review of bystander first-aid in low-middle income countries suggests training courses increase bystander knowledge, skill and provision of first-aid (1). It is unclear whether this increase in skill, or increased provision, represents an increased ability to recognise clinical signs or physiological parameters.

In a prospective observational study of trauma calls in Norway, bystander delivered first-aid was evaluated by attending ambulance personnel. Where intervention was indicated, bystanders performed airway intervention or recovery position in 81% of cases, haemorrhage control in 79% of cases and hypothermia prevention in 68% of cases (2). In another prospective observational study in Austria & Germany, positioning for airway control was performed, where indicated, in ~60% of cases, haemorrhage control in ~50% of cases and hypothermia prevention in ~40% of cases (3). These studies suggest bystanders are able to identify signs of compromised airway, major haemorrhage and hypothermia.

A study from the USA found laypersons overestimate simulated blood loss when <200ml, and underestimate when >400ml. Classification of bleeding as life-threatening increased with blood volume. Guidance suggests teaching laypersons to class blood loss >150ml as life-threatening. Only 17% of participants classed 150ml as life-threatening; >1000ml was required for 90% of participants to consider it life-threatening (4). These data suggests laypersons underestimate both severity and significance of blood loss.

Additional research is required to determine whether bystanders are able to identify and measure clinical signs and physiological parameters in the context of road injury. Further work is also required to assess whether training of bystanders improves recognition.

**Plain English Summary**

There is some limited evidence to suggest untrained members of the public can recognise casualties with ‘airway problems’ (a problem with their mouth or windpipe compromising their breathing), bleeding and hypothermia (risk of further harm from cold). In cases of bleeding, bystanders are likely to underestimate both the volume of blood loss and the severity of bleeding. There are many other signs and measurements that can be identified or recorded that may be of use to emergency medical services such as level of consciousness, heart rate or breathing rate. Further research is required to determine if bystanders can accurately assess parameters such as these.

**References**

1. Balhara KS, Bustamante ND, Selvam A, Winders WT, Coker A, Trehan I, Becker TK, Levine AC. Bystander Assistance for Trauma Victims in Low- and Middle-Income Countries: A Systematic Review of Prevalence and Training Interventions. Prehospital Emergency Care. 2019 May-Jun;23(3):389-410.
2. Bakke HK, Steinvik T, Eidissen SI, Gilbert M, Wisborg T. Bystander first aid in trauma - prevalence and quality: a prospective observational study. Acta Anaesthesiologica Scandinavica. 2015 Oct;59(9):1187-93.
3. Pelinka LE, Thierbach AR, Reuter S, Mauritz W. Bystander trauma care--effect of the level of training. Resuscitation. 2004 Jun;61(3):289-96.
4. Phillips R, Friberg M, Lantz Cronqvist M, Jonson CO, Prytz E. Visual estimates of blood loss by medical laypeople: Effects of blood loss volume, victim gender, and perspective. PLoS One. 2020 Nov 12;15(11):e0242096.

## SA13: Do context specific trauma training programmes for bystanders (lay-persons or non-clinical responders) confer measurable clinical or system benefits?

**Review**

PUBMED was searched on 22/09/2025 using the following search terms with MESH subheadings: Trauma AND Training AND Bystander OR Layperson. Results were restricted to English Language. An additional search using Google Scholar was conducted using the same key words. A total of 360 articles were identified through database searches, and a further 4 articles from Google Scholar. 14 underwent full text review. 6 articles were determined to be directly relevant to the review question.

Trauma focussed first-aid or first-responder training programs are numerous. In low-middle income countries (LMICs) these programs often focus on road injury due to high prevalence of road trauma or focussed on community specific concerns such as blast injury from mines. In high-income countries (HICs), particularly North America and Western Europe there are increasing numbers of haemorrhage-control courses.

Systematic reviews of trauma training programs in LMICs suggest these courses can increase lay-person skill, knowledge and confidence in trauma first-aid, although there are concerns regarding methodology and validity of assessment. A small fraction of reviewed studies reported clinical outcomes with improvements in mortality and physiological severity score (PSS) in areas with lay-person trauma training programs (1,2).

Prospective observational studies suggest prior first-aid training is associated with increased provision of first-aid for traumatic injury including establishing scene safety, casualty extrication and positioning, haemorrhage control and hypothermia management (3,4).

Haemorrhage control courses in HICs and LMICs have also been systematically reviewed with evidence of increased knowledge, skill and practice intention for laypersons. None of the reviewed studies reported on clinical outcomes (5,6).

There is some limited evidence to suggest trauma-focussed training programs may confer measurable clinical benefit. This has primarily been assessed using mortality which is likely the easiest to define and measure. Evidence regarding impact on morbidity, or system-level impacts is lacking. It is worth noting all mortality data comes from studies in LMICs and may not translate to HICs with established pre-hospital care systems.

**Plain English Summary**

Trauma focussed training programs for members of the public are available globally. There is a small amount of evidence, from low-middle income countries, that these programs may reduce deaths amongst casualties of traumatic injury, however there is no evidence available from high-income countries with established emergency care services. Furthermore, there is some evidence to suggest increased levels of first aid training increase the likelihood of first-aid being delivered, and delivered correctly, by members of the public. Further research is required to establish if these training programs reduce non-fatal injury, or offer other benefits to emergency care systems.

**References**

1. Balhara KS, Bustamante ND, Selvam A, Winders WT, Coker A, Trehan I, Becker TK, Levine AC. Bystander Assistance for Trauma Victims in Low- and Middle-Income Countries: A Systematic Review of Prevalence and Training Interventions. Prehospital Emergency Care. 2019 May-Jun;23(3):389-410.
2. Callese TE, Richards CT, Shaw P, Schuetz SJ, Issa N, Paladino L, Swaroop M. Layperson trauma training in low-and middle-income countries: a review. journal of surgical research. 2014 Jul 1;190(1):104-10.
3. Bakke HK, Steinvik T, Eidissen SI, Gilbert M, Wisborg T. Bystander first aid in trauma - prevalence and quality: a prospective observational study. Acta Anaesthesiologica Scandinavica. 2015 Oct;59(9):1187-93.
4. Pelinka LE, Thierbach AR, Reuter S, Mauritz W. Bystander trauma care--effect of the level of training. Resuscitation. 2004 Jun;61(3):289-96.
5. Consunji R, Mekkodathil A, Abdelrahman H, El-Menyar A, Peralta R, Rizoli S, Al-Thani H. Can “Stop The Bleed” training courses for laypersons improve hemorrhage control knowledge, skills, and attitudes? A systematic review. European journal of trauma and emergency surgery. 2024 Dec;50(6):2775-98.
6. Abdelrahman H, Mekkodathil A, El-Menyar A, Consunji R, Rizoli S, Al-Thani H. The effect of external bleeding control training courses on lay first-person responders' knowledge, skills, and attitudes in low-and middle-income countries: a systematic review. European Journal of Trauma and Emergency Surgery. 2025 Dec;51(1):252.

## SA14: Should more in depth follow ups/cognitive assessments be completed on patients involved in a high speed collision?

**Review**

The question of whether patients involved in high-speed collisions should routinely undergo more in-depth follow-up or cognitive assessment has not been directly answered in the trauma literature. No trials or comparative studies were identified that examine structured neurocognitive follow-up specifically in this patient group.

Evidence from traumatic brain injury (TBI) research demonstrates that even mild injuries, such as concussion, can result in persistent symptoms including cognitive impairment, fatigue, and psychological distress. High-energy mechanisms like road traffic collisions are strongly associated with TBI, even in the absence of abnormalities on acute imaging. Observational studies also show that patients discharged without structured follow-up may continue to experience unrecognised difficulties that impact recovery, return to work, and long-term quality of life.

Current guidance, such as NICE recommendations on head injury, supports providing information at discharge and follow-up for those with risk factors for persistent symptoms. However, it does not mandate universal cognitive assessment for all high-speed collision survivors. Trauma registry data also highlight the high prevalence of psychological morbidity after major trauma, yet specific follow-up protocols based on mechanism of injury remain limited.

In summary, while direct evidence is lacking, there is a strong rationale to consider structured follow-up, including cognitive or psychological assessment, for patients involved in high-speed collisions.

**Plain English Summary**

Patients involved in high-speed crashes may suffer hidden effects such as concussion, memory problems, or psychological distress, even if scans look normal. Research has not tested routine follow-up in this group, but evidence from head injury studies suggests it could help. Structured checks may support recovery and spot problems early.

**References**

1. Silverberg ND, Iverson GL. Etiology of the post-concussion syndrome: Physiogenesis and Psychogenesis revisited. NeuroRehabilitation. 2011;29(4):317-29.
2. National Institute for Health and Care Excellence (NICE). Head injury: assessment and early management. NICE guideline NG232. 2023.
3. Stulemeijer M, Vos PE, Bleijenberg G, van der Werf SP. Cognitive complaints after mild traumatic brain injury: things are not always what they seem. Journal of Psychosomatic Research. 2007 Dec;63(6):637-45.

## SA15: What is the scope and burden of postcrash injuries and mortality resulting from electric vehicle crashes?

**Review**

The scope and burden of post-crash injuries and mortality resulting from electric vehicle (EV) crashes have not been comprehensively characterised in the scientific literature. To date, most publications addressing EV safety focus on collision dynamics, battery fire risks, and vehicle engineering, rather than clinical outcomes for occupants or other road users.

Registry and epidemiological studies from high-income countries report overall trends in road traffic injury and mortality but rarely distinguish EVs from internal combustion engine (ICE) vehicles. Some transport safety analyses suggest that EVs may differ in crash profile due to factors such as increased vehicle weight, different acceleration patterns, and altered crash compatibility with smaller vehicles. These design differences could influence injury severity, particularly in collisions involving vulnerable road users. However, comparative injury or mortality data specific to EV crashes are limited.

There is emerging concern in case reports and safety reviews regarding post-crash risks unique to EVs, such as delayed thermal runaway, battery fires, toxic smoke exposure, and challenges for extrication and responder safety. These factors may indirectly influence patient outcomes by complicating rescue, delaying definitive care, or increasing secondary injury risk.

In summary, while the burden of road injury is well described globally, the specific contribution of EV crashes to injury patterns, mortality, and responder risk remains poorly quantified. This represents a growing evidence gap as EV adoption accelerates. Systematic linkage of crash registries, trauma databases, and fire/rescue records is needed to define the scale of the problem and guide prevention, vehicle design, and post-crash care strategies.

**Plain English Summary**

We don’t yet know exactly how electric vehicle (EV) crashes affect injury or death rates compared with other vehicles. Some studies suggest EVs may cause different injury patterns because they are heavier and accelerate differently, while case reports highlight risks such as battery fires and smoke. These hazards can complicate rescue and delay care. Overall, the impact of EV crashes on patients and responders remains poorly understood and needs further research as EV use increases.

**References**

1. Yu Q, Ma L, Yan X. Modeling occupant injury severities for electric-vehicle-involved crashes using a vehicle-accident bi-layered correlative framework with matched-pair sampling. Accident Analysis & Prevention. 2024 May;199:107499.
2. NTSB. Safety risks to emergency responders from lithium-ion battery fires in electric vehicles. National transportation safety Board Records management division, CIO-40490 L’enfant plaza. SWWashington, DC. 2020;20594.
3. Zhang B, Bewley RL, Tanim TR, Walker LK. Electric vehicle post-crash Recovery—Stranded energy issues and mitigation strategy. Journal of Power Sources. 2022 Dec 30;552:232239.

## SA16: Does moving EMS from a transport benefit to a treatment benefit (or hybrid) improve sustainability for EMS funding?

**Relevance**

In the UK, ambulance services are funded through the NHS as part of a block contract, not per patient transport. Unlike the US system, there is no financial penalty for treating patients at the scene rather than conveying them. Therefore, this funding-focused question is not relevant to UK practice.

**Recommendation**

Remove question

## SA17 - Moved to Group 1

## SA18: What are the experiences of family members/carers whilst the patient is receiving early hospital care for their injuries?

**Review**

The experiences of family members and carers during the early hospital care of trauma patients are poorly described in the literature. No studies focus exclusively on this phase for adult patients following road traffic collisions, but several related bodies of work provide important insight.

Qualitative studies in paediatric trauma show that family members frequently experience acute stress, anxiety, and uncertainty while their child receives emergency or intensive care following a traffic accident. Parents emphasise challenges in communication with clinical staff, a need for clear and timely information, and the emotional toll of witnessing acute interventions. Some also describe elements of post-traumatic growth in the aftermath of these experiences. Similar themes are found in broader trauma care, where caregivers describe distress linked to uncertainty about prognosis, difficulties navigating hospital processes, and unmet psychosocial needs.

Taken together, these studies suggest that family/carer experience during early hospital care is a critical but under-researched dimension of trauma. While findings are not specific to road injury or the earliest hospital phase, the recurring themes of communication, emotional distress, and need for support indicate this as an important gap for future qualitative and mixed-methods research.

**Plain English Summary**

Research on the experiences of families during early hospital care after road crashes is limited, though related studies in trauma and paediatrics highlight stress, uncertainty, and the importance of clear communication. Further road injury–specific qualitative work is needed.

**References**

1. Ni ZH, Lv HT, Wu JH, Wang F. Post-traumatic growth in caregivers of children hospitalized in the PICU due to traffic accident: a qualitative study. BMC nursing. 2023 Feb 23;22(1):48.
2. Wasilewski M, Reis L, Vijayakumar A, Leighton J, Hitzig SL, Simpson R, Mayo AL, Leslie GC, Vogt K, McFarlan A, Haas B. Peer support experiences and needs across the continuum of trauma care: A qualitative study of traumatic injury survivor, caregiver, and provider perspectives. Injury. 2025 Apr 1;56(4):112259.
3. Ohene LA, Power KJ, Raghu R. Parents’ perception of family centred care for children hospitalized through road traffic accident: A qualitative study at two tertiary settings in Ghana. International Journal of Africa Nursing Sciences. 2019 Jan 1;11:100176.

##

## SA19: Do resuscitation adjuncts that do not require refrigerated storage (e.g. TXA, calcium, fibrinogen concentrate, prothrombin complex concentrate) reduce early mortality either alone or in combination among trauma patients with hemorrhagic shock compared to standard care resuscitation?

**Review**

There is a substantial body of trauma literature examining the effects of these adjuncts. TXA is supported by large randomised trials (e.g. CRASH-2) showing reduced mortality when given early in bleeding trauma patients, though not specific to road injury. Calcium supplementation, fibrinogen concentrate, and prothrombin complex concentrate have been evaluated in trauma and perioperative contexts, with evidence largely derived from observational studies, small trials, or military series. These studies address trauma haemorrhage broadly and include patients from varied mechanisms such as penetrating and blunt injury, of which road traffic collisions form only a proportion.

The key point is that the efficacy of these adjuncts is a trauma-wide question. Their biological rationale and clinical effect are independent of the specific mechanism of injury. As such, the outcomes of interest (mortality, haemostasis, transfusion requirement) are not uniquely answerable for road injury patients.

A more road traffic collision related topic is already addressed more specifically in the related question on public access TXA for road traffic collision casualties, Group 2. That question focuses directly on RTC‑specific mechanisms, contexts, and bystander administration, whereas the present question concerns trauma haemorrhage more broadly and is not mechanism‑dependent.

Although important, this research area is already being studied in the context of trauma care as a whole. It is unlikely that road injury–specific evidence will provide additional value. We therefore recommend that this question is removed from scope for a road injury–focused prioritisation exercise.

**No References**

## SA20: What legal and data-sharing constraints exist for automated alerts from vehicles or wearables?

**Review**

Automated alerts from vehicles (telematics, automated driving systems) or wearables (health trackers, fitness sensors) raise complex legal and regulatory constraints in terms of data sharing. Key UK laws include the UK GDPR and the Data Protection Act 2018, which regulate what counts as personal data, special categories (e.g. health), lawful basis for processing, data subject rights, and responsibilities of data controllers/processors. ￼

In the case of automated vehicles, the Automated Vehicles Act 2024 and the Automated & Electric Vehicles Act 2018 introduce obligations for entities to collect or share safety-related data, particularly in use, for crash/incident investigation, regulatory monitoring, and insurance. These laws may require authorised self-driving entities (ASDEs) to make anonymised or limited datasets available under certain conditions. ￼

Wearables present different issues. Many commercial wearables are not medical devices under UK law, meaning that regulation (via MHRA) may not apply, limiting formal obligations around safety/data accuracy. Available privacy policies often permit manufacturers wide discretion over data collected, shared, or sold, unless elevated to medical device status or used under a clinician-patient arrangement.

Other constraints include consent (explicit, informed), purpose limitation (using data only for stated purposes), security obligations, anonymisation or pseudonymisation if data is shared beyond original controller, data subject rights, liability concerns (e.g. incorrect alerts, breach of privacy), and oversight (regulators such as ICO in the UK). Emerging proposals (e.g. EU Data Act, AI Act) may impose further obligations.

**Plain English Summary**

When phones, smartwatches, or automated cars send alerts using your data, UK and EU privacy laws limit how that data can be used. You generally must have consent, especially for health or special information, and companies must be clear about how they collect, store, and share it. Lawmakers are working on rules to balance safety, innovation, and your privacy.

**References**

1. Association of British Insurers (ABI). *Data sharing principles for automated vehicles*. London: ABI; 2024. Available from: https://www.abi.org.uk/globalassets/files/publications/public/motor/2024/data-sharing-principles-for-avs.pdf. Accessed 2025 Sep 22.
2. Information Commissioner’s Office (ICO). *Guide to the UK General Data Protection Regulation (UK GDPR).* Available from: https://ico.org.uk/for-organisations/uk-gdpr-guidance-and-resources/. Accessed 2025 Sep 22.
3. Radanliev P. Privacy, ethics, transparency, and accountability in AI systems for wearable devices. *Front Digit Health.* 2025;7:1431246. Available from: https://www.frontiersin.org/articles/10.3389/fdgth.2025.1431246/full. Accessed 2025 Sep 22.

## SA21: Pedestrians knocked down - how are they rescued from the road?

**Review**

The scientific literature does not directly address the question of how pedestrians struck by vehicles are “rescued” from the roadway. No dedicated body of evidence exists that specifically examines removal methods, whether by bystanders or professional responders, in this context. Instead, relevant insights are distributed across broader domains. Studies of bystander intervention emphasise the importance of ensuring scene safety, basic life support, and the risks and benefits of moving casualties. Research into extrication and patient handling, such as the EXIT project, highlights the principle of gentle movement and minimising unnecessary manipulation, but these studies primarily focus on trapped vehicle occupants rather than pedestrians on the carriageway. Operational safety guidance from emergency services literature covers traffic management, road closures, and responder positioning, but again does not treat pedestrian rescue as a distinct subject. Given these overlaps, the issue is perhaps best considered under three related headings: (i) bystander actions and safety at the scene, (ii) extrication and patient handling practices, and (iii) operational management of the crash environment. Framing the question within these existing domains ensures it is grounded in areas where empirical work and guidelines already exist, rather than maintained as an isolated research gap.

**Plain English Summary**

There isn’t any research that looks specifically at how pedestrians hit by vehicles are removed from the road. Instead, useful information comes from three related areas: bystander actions to keep people safe, safe ways of moving injured patients, and how emergency services manage the crash scene. It makes more sense to treat this topic under those headings rather than as a separate question.

**No References**

## SA22: How do we define 'bystander' in the context of road injury?

**Review**

Recent work confirms that the definition of “bystander” in the context of road injury is not consistently described in the scientific literature. While bystanders are frequently mentioned in studies of prehospital care, first aid, and resuscitation, the term is variably applied to untrained laypersons, trained lay responders, off-duty professionals, and even emergency services arriving informally before formal dispatch. This lack of precision complicates comparisons across studies and limits the generalisability of findings. Importantly, current international efforts are underway to clarify and standardise nomenclature in this field, recognising that consistent definitions are essential for evaluating interventions, training programmes, and bystander-enabled care pathways. Because active research is in progress to resolve this issue, this question—although highly relevant—should be deprioritised from the current process, avoiding duplication while ensuring that emerging consensus work is integrated once published.

**Plain English Summary**

At the moment, there is no clear agreement on what the word “bystander” means after a road crash. It could mean an untrained passer-by, a first aider, or even an off-duty professional who stops to help. Researchers are already working on creating a standard definition so that studies and training use the term in the same way. For this reason, this question should not be prioritised further.

**No References**

## SA23: Is prehospital imaging (e.g. handheld ultrasound) feasible or beneficial in guiding early transport decisions?

**Review**

The role of prehospital imaging, particularly point-of-care ultrasound (POCUS), has been increasingly explored in trauma and critical care. Feasibility studies across Europe, North America, and military settings demonstrate that ultrasound can be performed in the prehospital environment, including by helicopter EMS and ground paramedics with specific training. Reported applications include focused assessment with sonography for trauma (FAST/eFAST), lung ultrasound, and limited cardiac views. These studies confirm technical feasibility and acceptable image quality under field conditions.

The potential benefit of prehospital ultrasound lies in triage and transport decisions. For example, identifying free intraperitoneal fluid or absent cardiac activity may support prioritisation of direct transfer to a major trauma centre, activation of massive transfusion protocols, or termination of futile resuscitation. Similarly, thoracic ultrasound can aid diagnosis of pneumothorax or haemothorax when clinical findings are equivocal.

However, outcome evidence is limited. Observational series suggest changes in management occur in 10–30% of prehospital trauma cases where ultrasound is used, but no randomised controlled trials have shown improvements in survival or morbidity. Barriers include training requirements, maintenance of operator competence, device cost, and potential delays to scene time. Emerging technology (e.g. handheld ultrasound probes connected to smartphones) offers greater portability and integration with telemedicine, which may enhance feasibility.

Conclusion: Prehospital ultrasound is feasible and may influence clinical decision-making, particularly around transport and triage. However, robust evidence that it improves patient outcomes is lacking. Its role is best considered investigational, with further research required to establish benefit in routine trauma care.

**Plain English Summary**

Portable ultrasound machines can be used by ambulance or helicopter teams before a patient reaches hospital. They can sometimes show hidden bleeding or lung injuries and may help decide where a patient should be taken. Early studies show it is possible and occasionally changes care, but there is no strong proof yet that it improves survival or recovery. More research is needed before ultrasound can be recommended as standard practice in crash care.

**References**

1. Lucas B, Hempel D, Otto R, Brenner F, Stier M, Marzi I, Breitkreutz R, Walcher F. Prehospital FAST reduces time to admission and operative treatment: a prospective, randomized, multicenter trial. European Journal of Trauma and Emergency Surgery. 2022 Aug;48(4):2701-8.
2. Lin KT, Lin ZY, Huang CC, Yu SY, Huang JL, Lin JH, Lin YR. Prehospital ultrasound scanning for abdominal free fluid detection in trauma patients: a systematic review and meta-analysis. BMC Emergency Medicine. 2024 Jan 7;24(1):7.
3. Gamberini L, Tartaglione M, Giugni A, Alban L, Allegri D, Coniglio C, Lupi C, Chiarini V, Mazzoli CA, Heusch-Lazzeri E, Tugnoli G, Gordini G. The role of prehospital ultrasound in reducing time to definitive care in abdominal trauma patients with moderate to severe liver and spleen injuries. Injury. 2022 May;53(5):1587-1595. doi: 10.1016/j.injury.2021.12.008. Epub 2021 Dec 8. PMID: 34920877.
