# Supplementary material for: Identifying research priorities for post-collision care in the United Kingdom: outcomes and methodological adaptations from the final prioritisation workshop
Source: Scand J Trauma Resusc Emerg Med. 2026 May 27;34:127. doi: 10.1186/s13049-026-01628-y (PMC13397751; doi:10.1186/s13049-026-01628-y)
Supplement: Supplementary file 4 — Supplementary Material 4 [file 13049_2026_1628_MOESM4_ESM.pdf]

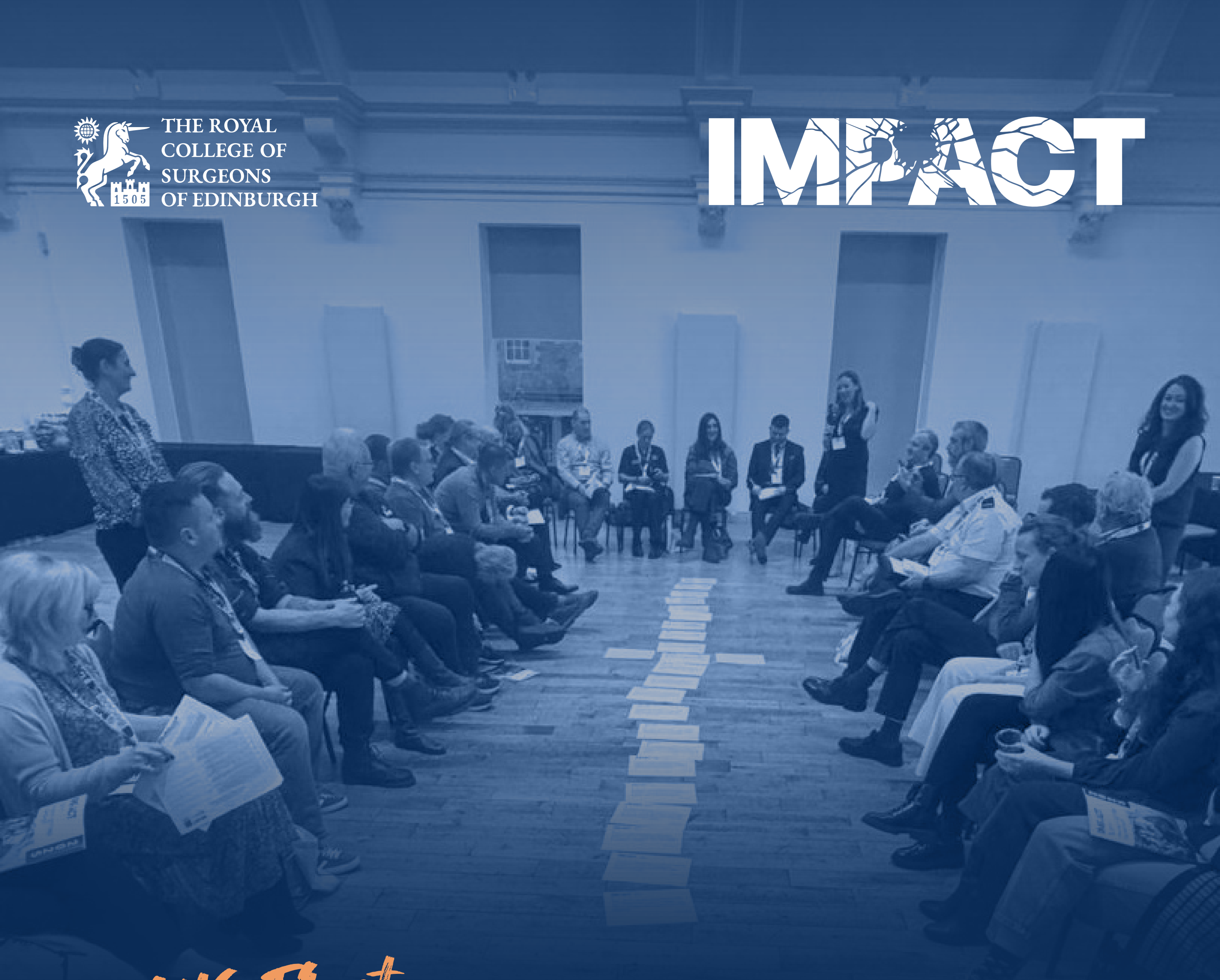

THE ROYAL COLLEGE OF SURGEONS OF EDINBURGH

IMPACT

a UK First

Road Injury, Post-Collision Care Priority Setting Partnership Report

vision zero SOUTH WEST

Devon Air Ambulance

TRANSPORT SCOTLAND CÒMHDHAIL ALBA

The Top Ten Research Priorities

1. After a road traffic collision, which types of deaths could potentially be avoided with the right help at the right time – and when are those critical moments where quick action can make the biggest difference?

2. What are the most reliable signs, at the scene of a road traffic collision, for spotting hidden life-threatening injuries such as internal bleeding or brain trauma?

3. How can police, fire, and medical teams work better together at the scene of a road traffic collision to speed up rescue and improve patient outcomes, and what training or systems support this best?

4. Can using technology, such as mobile apps, video calls, or live coaching, help people give better first aid and improve outcomes for those injured in road traffic collisions, when compared to just using a phone call for guidance?

5. What details should automatically be sent from a vehicle after a road traffic collision to help emergency services respond faster and more accurately, without sending false alerts?

6. What are the most effective ways to teach and support bystanders to provide first aid after road traffic collisions (including community training, telemedicine and digital tools), and how can the benefits of these approaches be measured?

7. What kinds of training, tools, and guidelines help emergency call handlers (dispatchers) better recognise serious injuries from road traffic collisions and send the right help quickly?

8. In road traffic collisions, how do factors such as age, gender, and background influence the types of injuries people get and the care they receive, and what can be done to reduce inequalities?

9. Which urgent treatments work best for people trapped after a road traffic collision, and how can non-medical responders safely provide them?

10. What does a successful recovery after a road injury really mean, and are researchers measuring what matters most to patients?

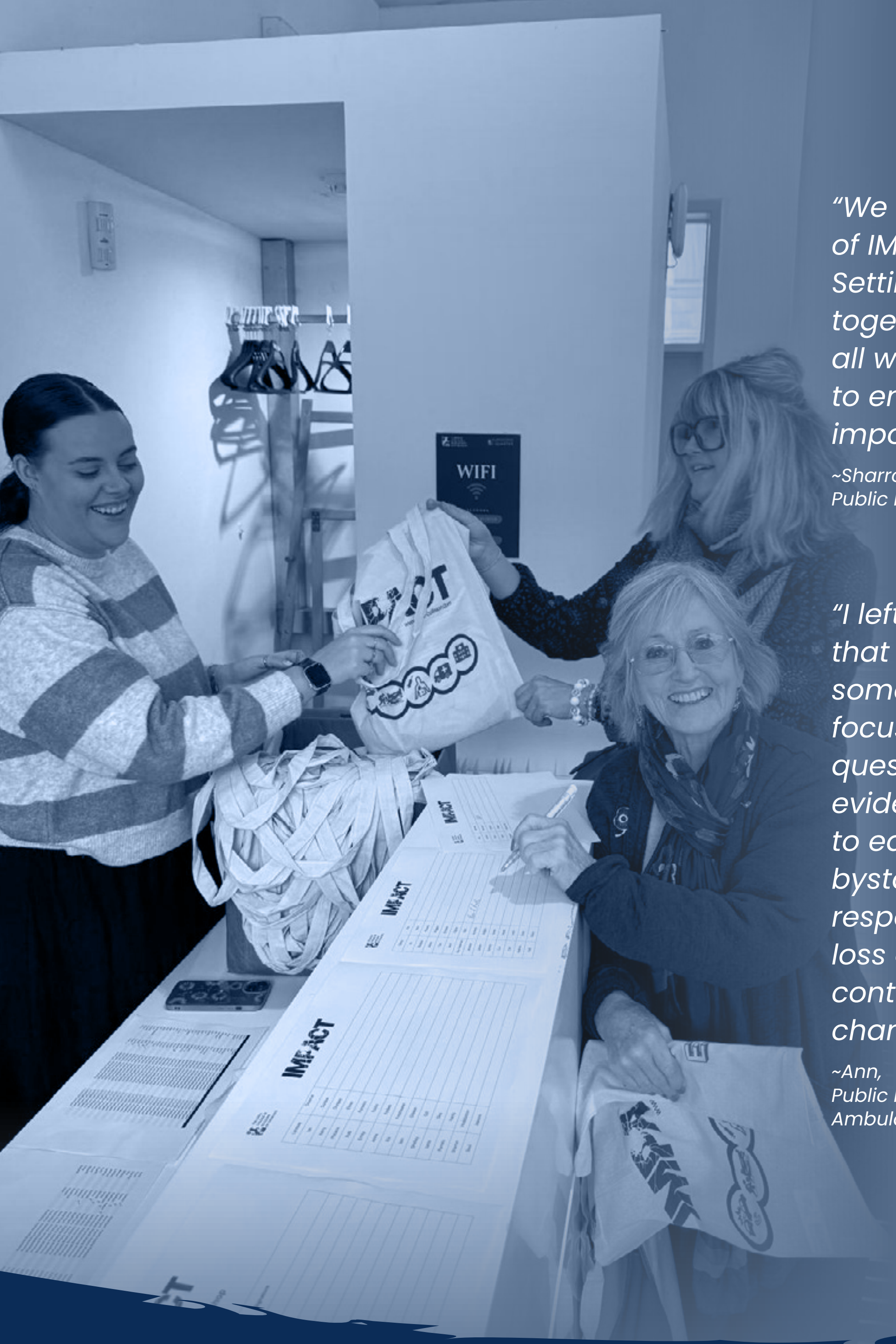

"We were so grateful to be part of IMPACT's Research Priority Setting Workshop... it brought together genuine passion from all who were brought together to engage and discuss the important issues"

~Sharron and Nicola,
Public Participants

"I left Edinburgh feeling hopeful, that this is the beginning of something transformative. By focusing research on the right questions, we can develop evidence-based strategies to educate and empower bystanders, improve emergency responses, and prevent needless loss of life. To everyone who contributed: your voices will change the future."

~Ann,
Public Participant and founder of Devon Air Ambulance

The success of this process was made possible by individuals with lived experience of road injury, whether through their professional role, as a bystander, as a family member or carer of someone affected, or as someone who has personally experienced a road injury.

Contents

SECTION 1

P5. Introduction
P6. Project Brief
P9. Our Process
P10. Our Participants

SECTION 2

P11. Our Methodology
P14. Pre-Workshop Survey
P15. Count of Professional category
P18. Trauma Informed
P20. The Top Ten

SECTION 3

P26. Emerging themes & Further Reflections
P31. What we found
P34. What happens now?

ADDITIONAL INFORMATION

P37. Glossary
P38. Acknowledgments
P39. Professionals
P42. References

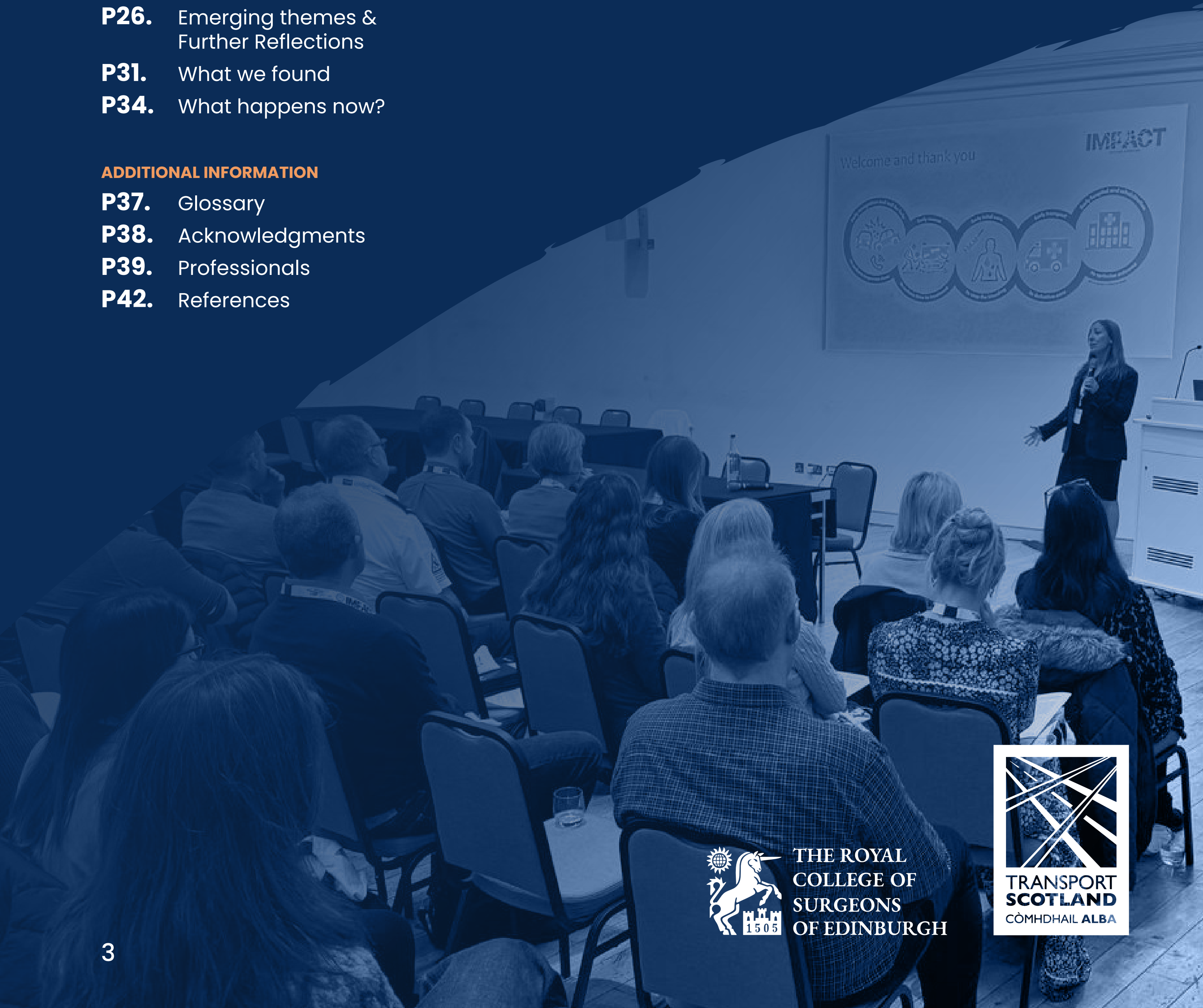

THE ROYAL COLLEGE OF SURGEONS OF EDINBURGH

TRANSPORT SCOTLAND
CÒMHDHAIL ALBA

“A road traffic collision doesn’t just affect those immediately involved, but sends ripples into families, schools, businesses across our communities. It is vital that we get post-collision care right, minimising the physical and emotional toll of these incidents as much as we can.

We would like to thank IMPACT and all those involved in this workshop for developing and prioritising these research questions so we can increase our understanding of where action, and resources, should best be spent”.

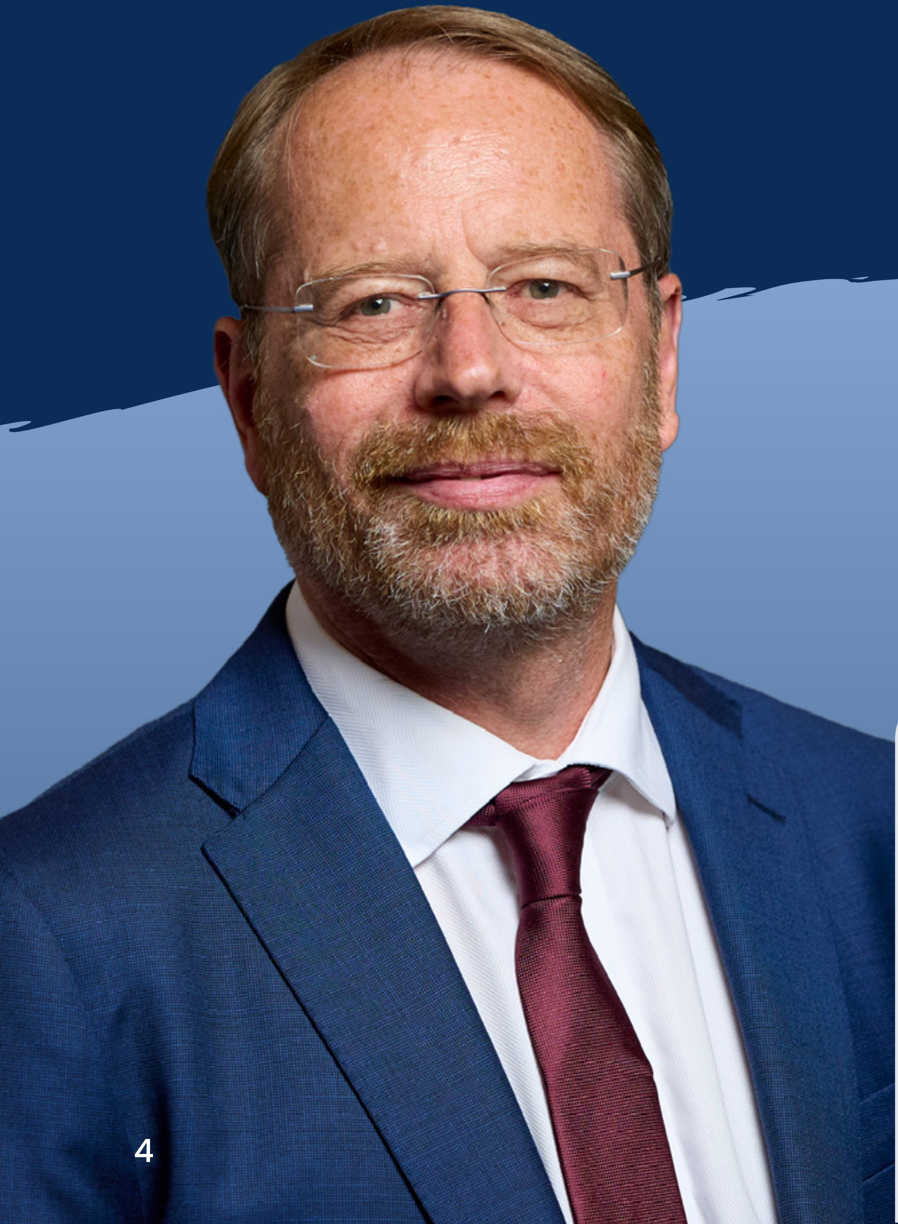

Andy MacNae MP,

President of PACTS and the Chair of the Transport Safety APPG

Introduction:

This was the first Road Injury, Post-Collision Care Priority Setting Partnership (PSP) in the UK, the process highlighting the diverse, complex and multi-faceted aspects of those involved in road traffic collision injury - and the gaps in research and innovation within this space.

This PSP marks a significant milestone in shaping the future of post-collision care. The final priorities outlined in this report provide a clear roadmap for research, policy, and service development, placing the voices of those with lived experience at the heart of the process, with the ultimate goal of improving patient outcomes.

These priorities will serve as a long-term framework for integrating post-collision care into national and international research agendas, guiding protocol development, knowledge translation, implementation science, and global collaboration to drive meaningful improvements in survival and recovery.

This is just the beginning of this journey, our ambition is to centre these priorities within research, policies at all levels, innovation, and system working, future partnerships. Most importantly, our end goals are to end preventable deaths and disability, and improve health outcomes for all those involved in road traffic collisions regardless of age, sex, ethnicity or background.

Project Brief

Road injury remains a leading cause of death and disability worldwide. Approximately 1.2 million people are killed in road traffic collisions each year, with a further 20–50 million individuals injured and often left with long-term disabilities.

While significant progress has been made in preventing road traffic collisions through safer vehicles, better road design, and enforcement of traffic laws, many deaths and serious injuries are still caused by gaps in the care that follows a road traffic collision. Delays in recognising severe injuries, difficulties in rescuing trapped victims, inconsistent first aid, slow transport, and variability in hospital trauma care all contribute to preventable poor outcomes.

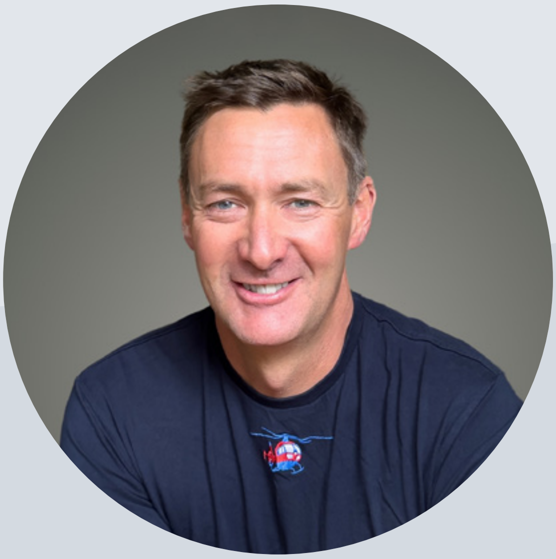

"Road traffic collisions happen everywhere, but the way emergency care is delivered varies widely. To save more lives, we need a structured, evidence-based approach".

~Professor Tim Nutbeam,
Professor of Emergency Medicine

Director, IMPACT -
Centre for Post-Collision Research, Innovation and Translation

In other medical emergencies, such as a heart attack, survival rates improved dramatically when a clear "Chain of Survival" was introduced, a series of essential steps that must happen quickly and effectively. We've adapted this idea for road injuries, creating five critical links in the care process, from the moment of the road traffic collision through hospital treatment and rehabilitation.

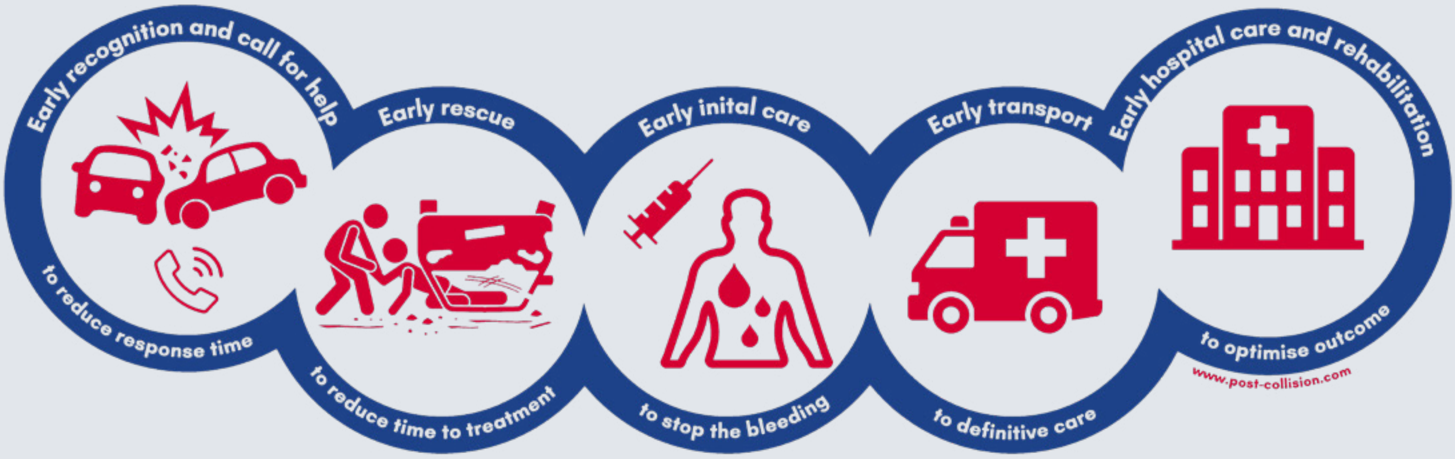

By strengthening every link, we reduce delays, improve care quality, and give patients the best chance of survival and recovery. This framework also helps ambulance services, hospitals, and policymakers collect better data and focus improvements where they matter most. Our goal is a shared vision for post-collision care, bringing different sectors together to save lives.

This PSP centred around the James Lind Alliance methodology, with adjustments and accommodations made throughout to enable access for those with lived experience of road traffic collision injury.

We began by selecting and inviting a small group of people with the relevant expertise and experience, to help guide and oversee the project - this was our Steering Group. Ours was made up of patients with lived experience of serious road traffic injury, family members, representatives from; fire and ambulance services, clinicians, trauma psychologists, researchers and policy partners.

We launched a national survey, asking people with lived experience, either within their professional role or as a member of the public, to submit questions they'd like answered by research.

“What do you think needs to be better understood, improved or changed?”

We gathered a total of 179 questions, with an additional 73 questions from other research literature, giving us 252 questions - these were refined and themed, producing 34 overarching questions and a further 23 standalone questions.

We then held a short ‘interim prioritisation’ process, which produced 23 questions to take to an in-person workshop, where structured breakout group conversations produced a final ‘Top Ten’ Priorities. The workshop was attended by a diverse range of professionals and members of the public with lived experience of road injury.

“After 30 years in the police and seeing the impact of fatal and serious road traffic collisions first hand, how good it was to be able to contribute to research which will transform road safety.”

~Marilyn MacQueen, IMPACT PPI and PSP Steering Group member

Our Process

1. Establish a Steering Group
This group takes responsibility for the PSP process, including oversight, direction and planning. It is made up of carers, members of the public and a range of professionals with lived experience of post-collision road injury.

2. Collect questions
Ask a wide range of people with lived experience to submit any questions they'd like to be researched. Review existing literature to see if these have already been answered or if there are any gaps.

3. Summarise the responses gathered
Questions are reviewed and sorted into themes, creating summary questions for research.

4. Check the evidence
This long list of summary questions is then checked against existing literature to ensure they are unanswered by research; questions that have already been addressed are removed.

5. Create a shortlist of summary questions
The long list of summary questions are put through a prioritisation process in order to produce a shortlist to be discussed at a workshop.

6. Hold a Workshop
The shortlist of questions is discussed in a structured workshop of people with lived experience (professionals, carers, patients, public) who collectively agree on a final Top Ten priorities for research.

Our Participants

Questions regarding personal characteristics and circumstances were self-reported and optional at certain stages of this process.

All of our participants were over the age of 18 and had lived experience within the UK.

We collected participants' demographic from all stages of the process, including at question collection stage and from workshop participants. From those participants that provided demographic information:

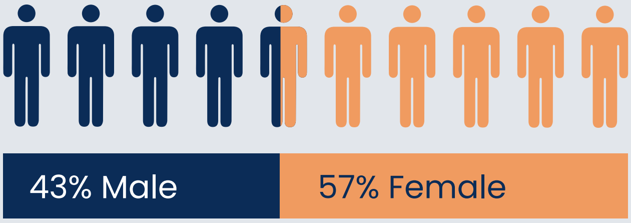

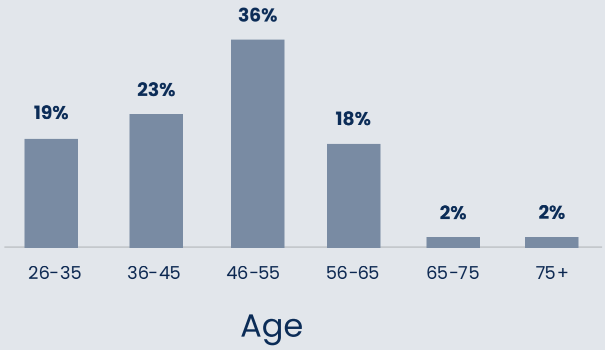

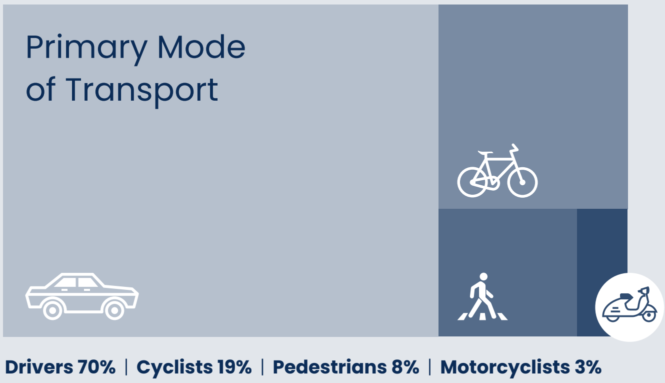

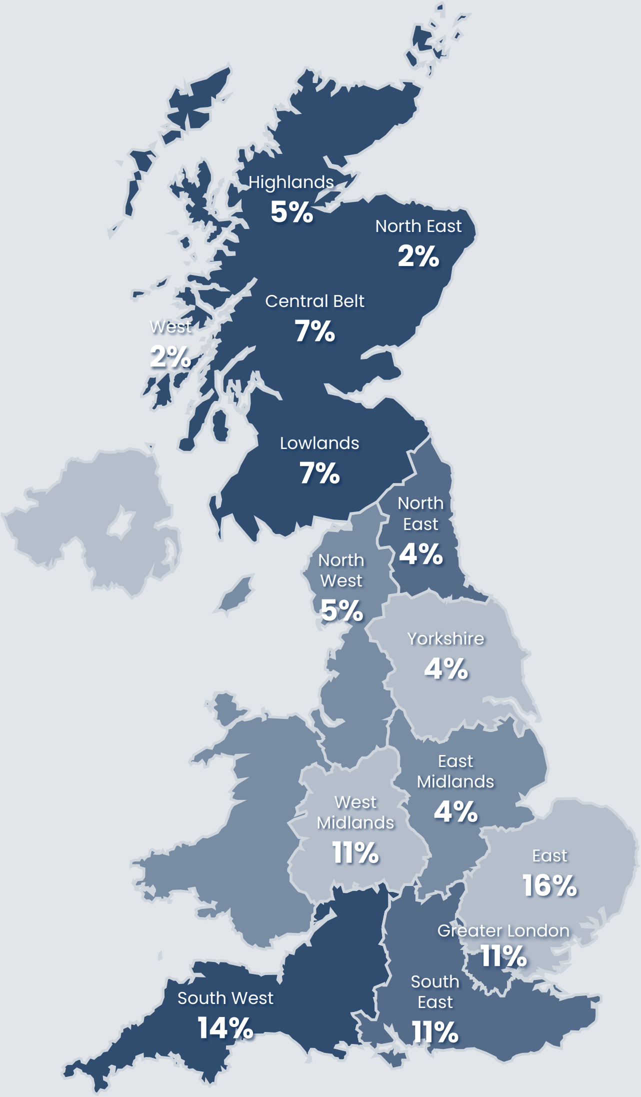

"I had such a great day, learnt so much and am incredibly inspired by the discussion. Having members of the public in attendance reminded me of why we do what we do."

~(Healthcare professional)

SECTION 2

Our Methodology

1. Steering Group

The PSP was overseen by a Steering Group, composed of patients with lived experience of serious road traffic injury, family members, representatives from fire and ambulance services, clinicians, trauma psychologists, researchers, and policy partners. The Steering Group provided strategic direction throughout the process, including developing the research plan and approach, ensuring the questions were within the boundaries of this project, and in preparing for the final prioritisation workshop.

2. Scope of Project

This Priority Setting Partnership is focused on the Road Injury Chain of Survival, which begins at the moment a road traffic collision occurs and continues through rescue, care, transport, and rehabilitation. We welcomed questions that aim to improve any part of this post-collision response, for example, how bystanders can be better supported to give first aid, how rescue services can work more effectively, or how recovery and rehabilitation can be improved.

Scope Included:

- **Early Recognition and Call for Help –** Spotting serious injury and getting help quickly.
- **Early Rescue –** Safely freeing people trapped in vehicles.
- **Early Initial Care –** Giving first aid at the scene, often by bystanders or emergency responders.
- **Early Transport –** Getting injured people to the right hospital without delay.
- **Early Hospital Care and Rehabilitation –** Treating injuries and supporting recovery in hospital and beyond.

Scope Excluded:

- Road traffic collision prevention strategies
- Pre-road traffic collision behaviour or enforcement
- Infrastructure improvements (road layout, signage, etc)
- Vehicle safety technology focussed on prevention
- Policy or legislation focussed on prevention
- Prevention Education Campaigns

3. Initial Survey

Potential research questions were submitted through a national open survey distributed across patients, carers, bystanders, clinicians, rescuers and other professionals. The survey opened on 4th July 2025 and closed on 31st August 2025, and was publicised on the IMPACT website, shared through IMPACT social media channels, and circulated via the Centre's bi-monthly newsletter to broaden visibility and encourage participation.

Responses were provided in free-text format and were not restricted to predefined categories.

We received 179 questions and added an additional 73 questions from relevant research literature. Only four questions were out of scope for this PSP.

What drove these questions:

Some respondents provided context to their questions. Across submissions, there was a consistent call to translate evidence into practice, specifically embedding medical decision making within extrication, standardising bystander definitions and protocols, and tackling 'persistent myths' with accessible, sensitive communication.

Participants highlighted the reality of 'stretched resources', urging 'right-time/right place' investment, smarter dispatch and minimising non-essential activity. Lived-experience contributions emphasised both physical and psychological risk, reinforcing the need for family/carer support and trauma-informed approaches throughout.

4. Collection, Analysis and Evidence Checking.

To ensure complete confidentiality and compliance, we had the project lead separate the data into the following:

- **Master Spreadsheet:** only accessed by the Project Manager
- **Tracker Spreadsheet:** only accessed by the Project Manager, entries were anonymised and demographic information separated, codes and references were embedded into the question cells.
- **Working Spreadsheet:** accessed by the Steering Group and the PSP project team.
- Questions were reviewed, checked for existing evidence and then grouped based on themes, and overarching questions were produced. Standalone questions were also identified and recorded.

This process produced 34 overarching questions and 23 standalone questions which were then individually reviewed to see if they had already been researched. These questions were then longlisted as 41 questions.

5. Create a shortlist of summary questions.

The confirmed longlist of questions were reviewed by the Steering Group to decide which ones should move forward. Each member scored every question from 1 to 5 based on the level of priority, relevance and how impactful they thought it was. We then applied the median for each question, which provided a noticeable drop in score after the 23rd question, providing a manageable number to move forward to the workshop.

The questions were then re-written in plain and accessible language, with lay summaries, ensuring they could be understood by the diverse range of stakeholders at the workshop.

Pre-Workshop Survey for members of the public

We recognised that there would be members of the public with lived experience of road injury who might not be able to physically attend the workshop due to the nature of their injuries. We created an online survey which could be completed either on a laptop or via a smart device, with the accompanying guides and information.

We worked with several patient and public facing organisations who supported the selection and recruitment of individuals with lived experience. We had responses from three members of the public, these were displayed on the questions sheets at the workshop and used to supplement the workshop discussions.

Public Breakdown:
*some members fall into more than one category

Members of the public (7)	
Family members of someone road injured	4
Survivor of road injury	3
Bystander	3
**Professionals (34)**	
Police/Fire and Rescue	4
Healthcare Professionals (including Technicians, Paramedic, Doctor, ACP)	13
Medical College	5
Third Sector (including organisations involved with support and advocacy, government, health, research and work-related road safety)	6
Private (including consultancies who specialise in data and research in post-collision investigation and car safety, legal sector and rescue services)	5
University	1

"Fantastic day. I learned so much and the perspectives of others changed some views of my own and helped me to have a far bigger understanding of other services."

~(third sector professional)

Count of Professional Category

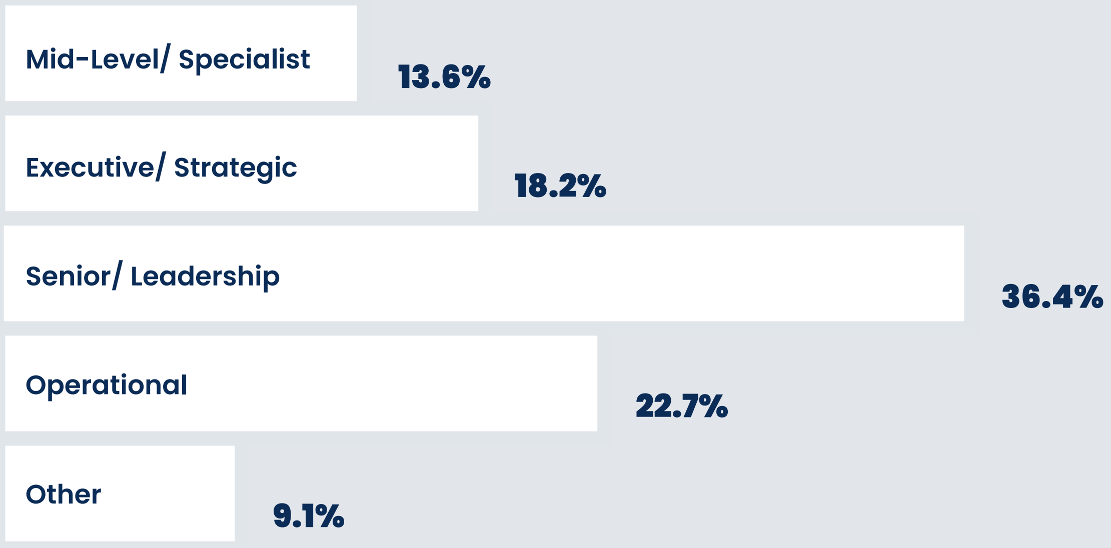

The primary objective of the workshop was to jointly agree the final Top Ten questions. A Nominal Group Technique (NGT) was used. Put simply, this is a structured approach that enables a group to generate ideas and agree priorities, with the overall aim of ensuring that everyone has an equal voice.

It provides a framework for input from people with different roles and levels of confidence, and supports the group in moving towards a clear, shared set of priorities.

Participants were divided by the PSP Chair into three groups of approximately 13 people, ensuring that each group included more than one member of the public.

Each group was supported by a facilitator. In addition, a trained counsellor was present throughout the day and moved between groups to provide reassurance and respond to any needs that arose.

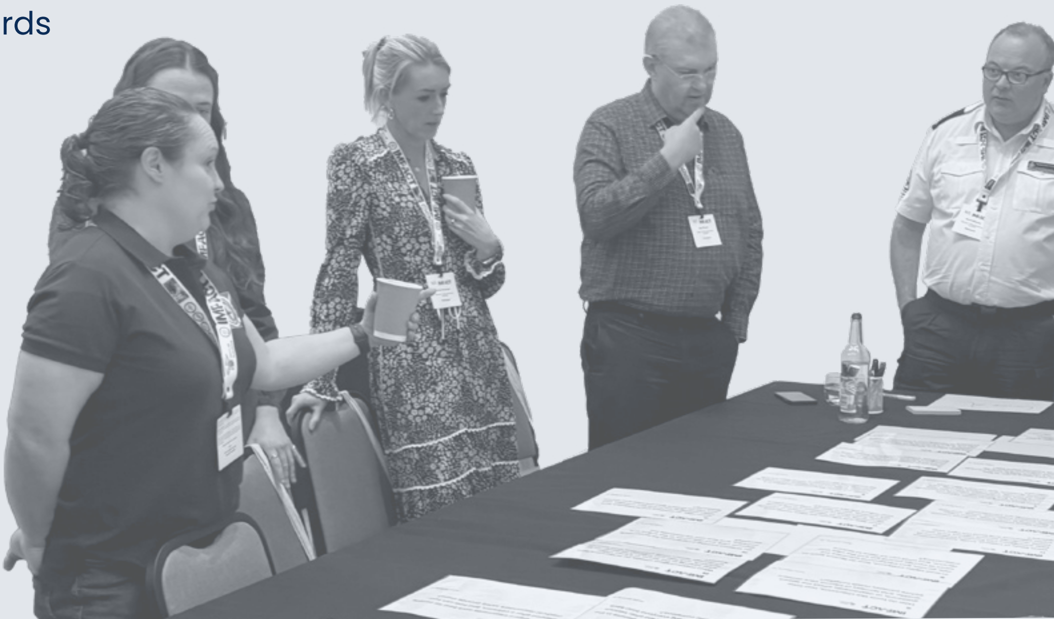

The Workshop Process

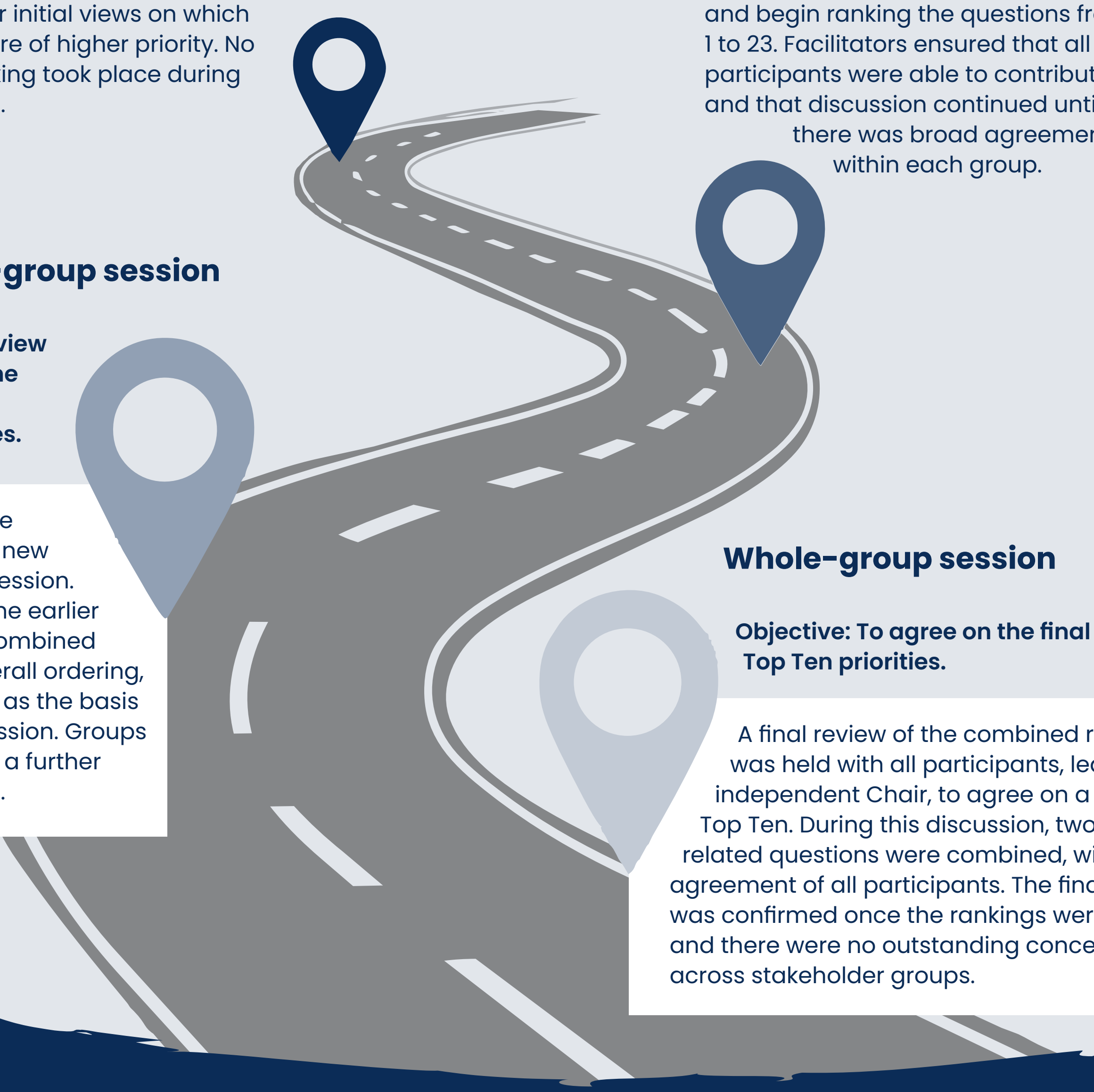

First small-group session

Objective: To allow participants to share views and reflect on the pre-workshop review of the questions.

Participants discussed the questions and shared their initial views on which they felt were of higher priority. No formal ranking took place during this session.

Second small-group session

Objective: To carry out an initial ranking of the questions.

Participants remained in the same groups and were invited to discuss and begin ranking the questions from 1 to 23. Facilitators ensured that all participants were able to contribute and that discussion continued until there was broad agreement within each group.

Final small-group session

Objective: To review and further refine rankings using combined scores.

Participants were reallocated into new groups for this session. Rankings from the earlier sessions were combined to create an overall ordering, which was used as the basis for further discussion. Groups then completed a further ranking exercise.

Whole-group session

Objective: To agree on the final Top Ten priorities.

A final review of the combined rankings was held with all participants, led by an independent Chair, to agree on a shared Top Ten. During this discussion, two closely related questions were combined, with the agreement of all participants. The final order was confirmed once the rankings were stable and there were no outstanding concerns across stakeholder groups.

“Facilitating the PSP showed me how important it is to make decisions together. The supportive, open atmosphere brought professionals and people with lived experience into honest conversations that shaped clear, meaningful priorities”

~Dr Emily Foote, Facilitator

Engaging with the public

We embedded a trauma-informed approach throughout the PSP process, ensuring that participants had opportunities to meet and discuss the process, the objectives, their role and provide explicit reassurances that they could withdraw at any point, without judgement, question or repercussion.

We adopted the perspective that participation in these activities could be therapeutic, providing a consistent framework for facilitation, evaluation, and alignment with IMPACT's long-term public engagement strategy.

***"The Teams meeting beforehand meant I was well prepared and confident coming into the day. I appreciated being re-assured that I could leave at any point and felt in control of what I felt I could be involved in. I appreciated being greeted and introduced to people and also the workshop sessions were well run, making sure everybody was able to express their views...I think having people who had experienced RTCs in each group was beneficial as it is very different from being a professional at the scene.
I also learnt a lot which was a bonus."***

~(member of the public with road injury lived experience)

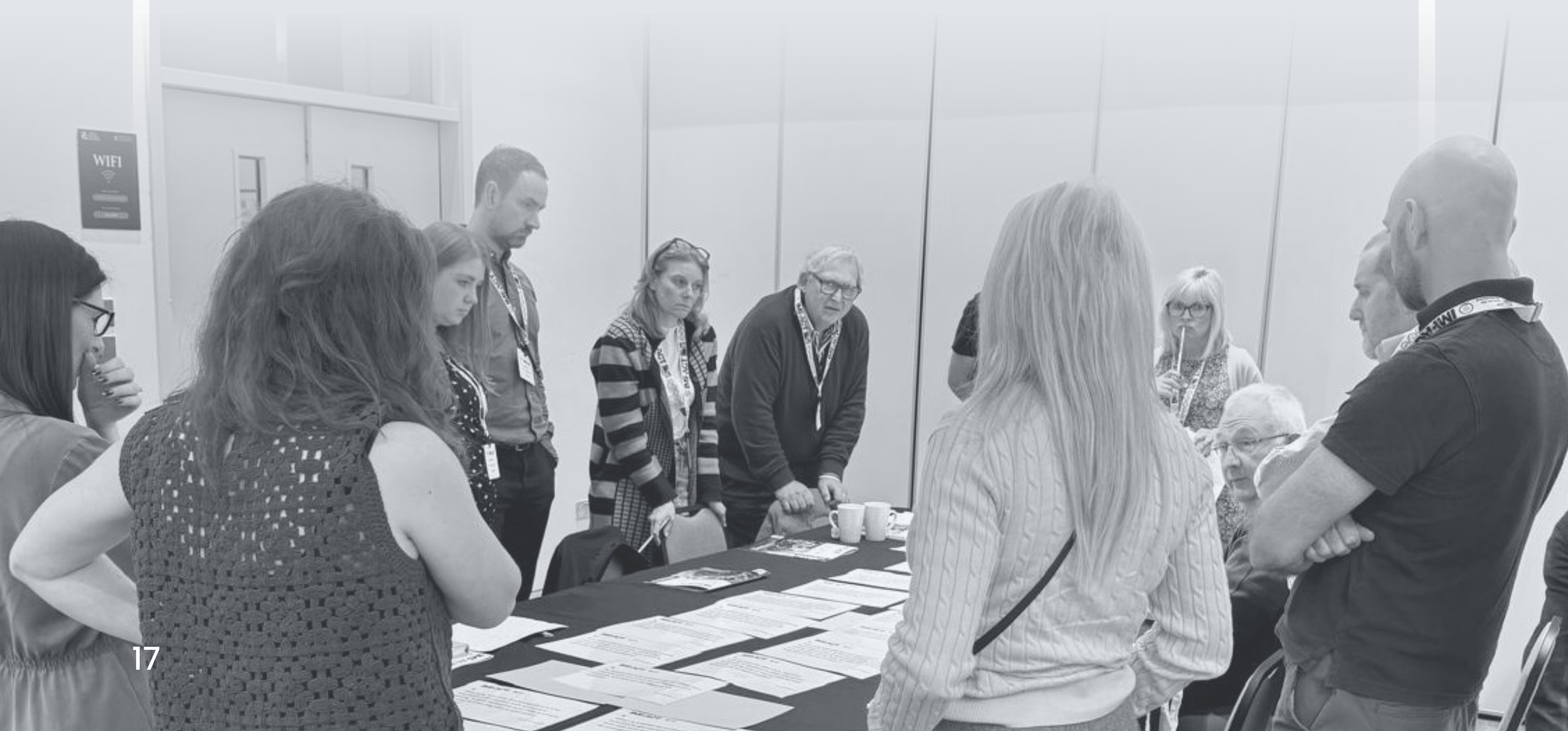

Safety, Trustworthiness, Choice, Collaboration, Empowerment, Cultural Consideration

1 Steering Group/ Critical Friends

Composed of clinicians, researchers and two PPIE members to ensure the PSP process was:

- Grounded in trauma-informed principles
- Lived-experience perspectives informed language
- Survey design
- Safeguarding measures
- The creation of safe, supportive spaces for participants

2 Survey

- Accessibility, alternatives methods offered and provided
- Guides, handbooks and information provided

3 Pre-Workshop

- Pre-meet via Teams or Zoom
- Accessibility needs discussed
- Recruitment supported by specialist services
- Facilitator briefings included:
    - A summary of the nature of lived experience they would have in their group
    - Training on including the public voice in discussion, ensuring support, empowerment and inclusion.

Trauma Informed

We used a trauma-informed approach throughout, which acknowledges the impact of trauma on people with lived experience and responds to six principles. We integrated this understanding into how we designed, communicated, and made decisions within the PSP, so that all participants feel safe, respected, and able to contribute fully.

4 Pre-Workshop

- Recruitment of specialist pastoral support.
- Provision of private Wellbeing Room.
- Venue assessed and accessibility arrangements made.
- Each breakout group had no less than two members of the public to ensure peer to peer support.
- Remote rankings were integrated into group discussions to ensure equal voice and influence while preserving consensus.
- Facilitators de-briefed with members of the public following prioritisation exercises to ensure they'd felt heard.
- Members of the public had a designated person to meet and greet.

5 Post-Workshop

- Follow up communications were sent to check in and offer additional follow up support if needed.
- Evaluation included.

Cross-cutting theme:

Feedback on language, tone and layout of communications was sought and provided at every stage by both professionals and members of the public.

"I appreciated being involved in this research and found reading the papers and the subsequent 'discussions' very interesting, informative and stimulating. The expertise of all those taking part was clear to me as a lay person and I hope that this research will promote a greater shared practice to the benefit of those who need post-collision care in the future".

~ Brian Lee,
member of the public and
Steering Group member

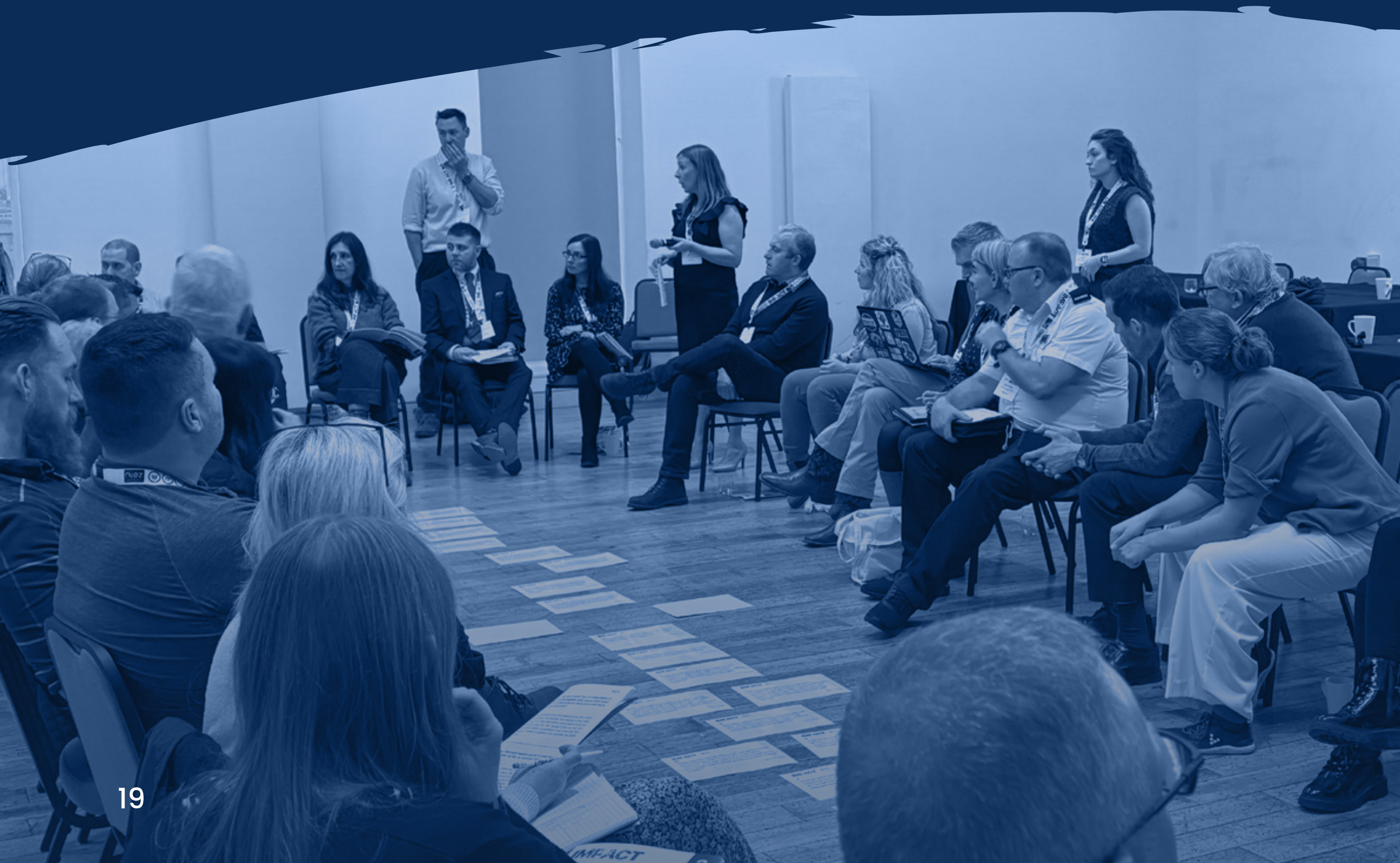

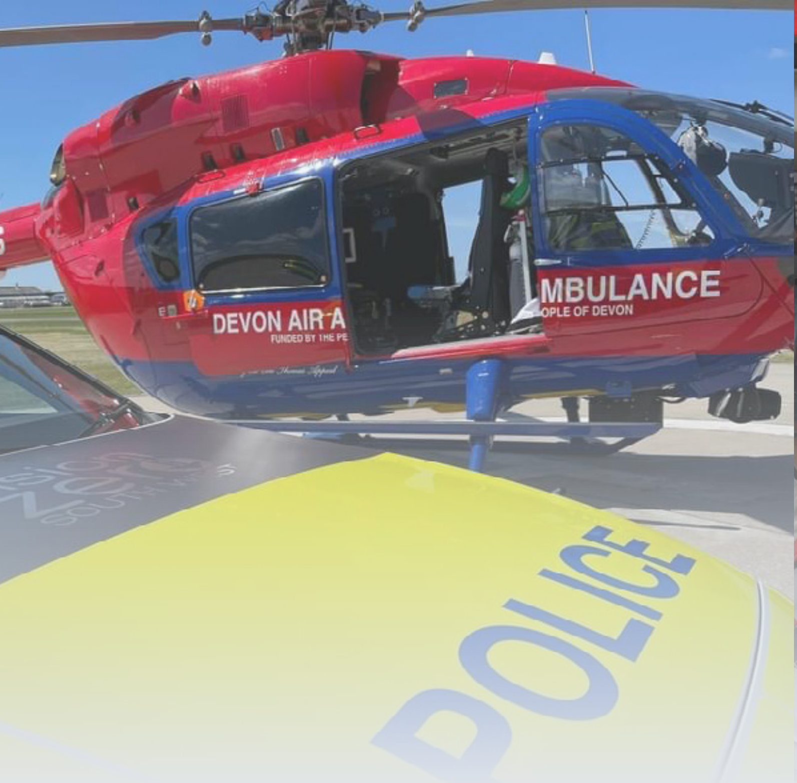

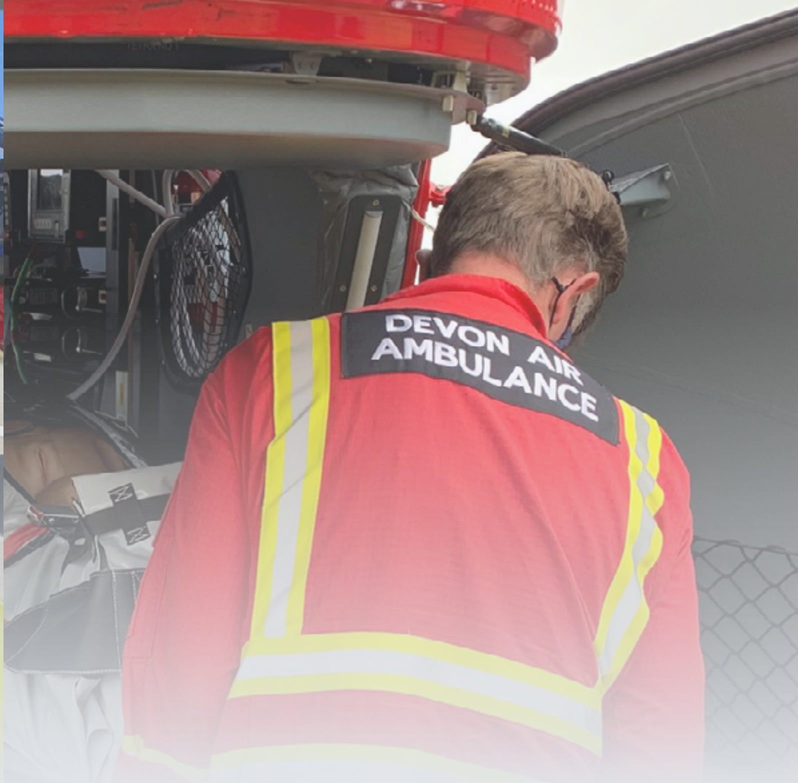

The Top Ten

Following this rigorous and collaborative PSP process, which gathered questions from across the community and refined them through multiple stages of evidence checking and prioritisation, we are pleased to present the Top Ten research priorities, each accompanied by a plain-English summary.

1.

After a road traffic collision, which types of deaths could potentially be avoided with the right help at the right time – and when are those critical moments where quick action can make the biggest difference?

Injuries from road traffic collisions lead to many deaths worldwide, mostly from head injuries and severe bleeding. Across studies, up to 80% of these deaths could be prevented if people received faster, better care.

Time is critical. Around one in three deaths happen in the first hour, and treatment within the first 15 minutes offers the best chance of survival. Quick actions such as stopping bleeding, managing the airway, and getting patients to hospital rapidly can save lives.

In lower-income countries, preventable deaths are more common due to limited access to advanced trauma care and delays in reaching treatment. Overall, improving outcomes after serious road traffic collisions requires faster emergency responses, better prehospital care, and rapid transfer to specialist centres. Integrating these steps into coordinated trauma systems can help ensure people get life-saving treatment when they need it most.

2.

What are the most reliable signs, at the scene of a road traffic collision, for spotting hidden life-threatening injuries such as internal bleeding or brain trauma?

'Occult' injuries are serious internal injuries from road traffic collisions that are not obvious at first glance. A person might look fine at the scene, but still have life-threatening damage, especially to the head or abdomen. Researchers have developed scoring tools, like the Occult Score and Transfer Score, to help identify these hidden injuries and decide who should go to a trauma centre. These tools use road traffic collision details, not just visible injuries or vital signs. However, more work is needed to test these tools in real-life settings, especially in rural or lower-income areas, and to help dispatchers and bystanders use this knowledge effectively.

3.

How can police, fire, and medical teams work better together at the scene of a road traffic collision to speed up rescue and improve patient outcomes, and what training or systems support this best?

When someone is trapped in a vehicle after a road traffic collision, police, fire, and medical teams all need to work together quickly. Research shows that delays often happen when roles are unclear or when too many responders crowd the scene. Newer strategies, like the EXIT method and joint training between fire crews and paramedics, can make rescues faster and safer. However, these approaches are not used everywhere, and still lack strong evidence on how they affect patient survival. More real-world testing and better coordination policies are urgently required.

4.

Can using technology, such as mobile apps, video calls, or live coaching, help people give better first aid and improve outcomes for those injured in road traffic collisions, when compared to just using a phone call for guidance?

New technology like apps, video calling, and real-time coaching can help bystanders give better first aid in emergencies. These tools have been shown to improve survival after cardiac arrest by helping people start CPR quickly and do it properly. However, most tools focus on heart-related emergencies, not the kinds of injuries seen after road traffic collisions. For trauma, evidence is still limited. Some studies show dispatcher help by phone may support life-saving actions, but results are mixed. More research is needed to develop technology that works in road traffic collision situations and gives clear, fast help for bleeding, airway problems, and other trauma injuries.

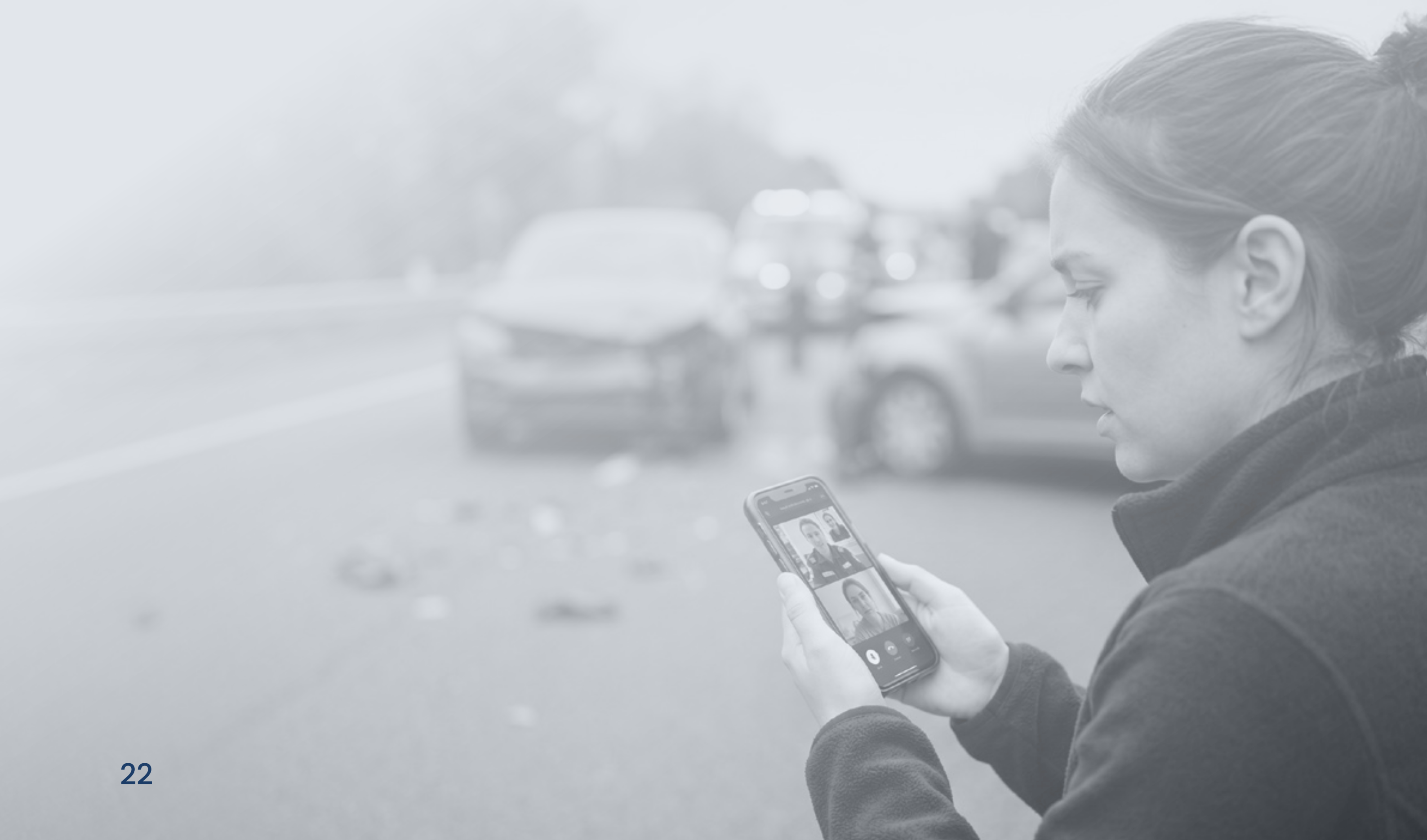

5.

What details should automatically be sent from a vehicle after a road traffic collision to help emergency services respond faster and more accurately, without sending false alerts?

eCall and Advanced Automatic Crash Notification (AACN) systems help emergency services respond quickly to serious road traffic collisions. They work by automatically sending key information such as crash severity, seatbelt use, airbag deployment, and exact location to dispatch teams. This helps decide what level of help is needed, even if the people involved can't call for help themselves. Research shows these systems can reduce missed serious injuries and improve response. However, they can also send too many false alarms. To improve, systems must reliably send data, even in rural areas, and use smarter algorithms that learn from past road traffic collisions to better predict who needs urgent care.

6.

What are the most effective ways to teach and support bystanders to provide first aid after road traffic collisions (including community training, telemedicine and digital tools), and how can the benefits of these approaches be measured?

Teaching members of the public how to recognise and respond to life-threatening injuries after a road traffic collision can save lives, and simple tools such as the sBATT score show that serious trauma can often be identified using visible clues, such as confusion, heavy bleeding, fast breathing, or being trapped in a vehicle, without the need for medical equipment. Early evidence suggests that bystanders can sometimes recognise critical problems such as airway issues, bleeding, or hypothermia, but they often underestimate how severe bleeding is and may struggle to judge how urgently help is needed. Many other signs, including changes in alertness, heart rate, or breathing, could potentially support emergency responders, though it remains unclear whether bystanders can reliably assess them.

As new approaches such as community training, telemedicine support, and digital tools become more common, further research is needed to understand which methods most effectively teach and support bystanders to provide first aid after road traffic collisions, and how best to measure the benefits of these approaches in real-world situations.

7.

What kinds of training, tools, and guidelines help emergency call handlers (dispatchers) better recognise serious injuries from road traffic collisions and send the right help quickly?

When someone is badly hurt in a road traffic collision, emergency call handlers (dispatchers) must quickly decide what help to send. Current systems often miss serious injuries or send too much help. Some new tools, like computer programmes that analyse what callers say, can support dispatchers, especially when they are unsure. Training and clear protocols also help, but many are not specific to road traffic collisions. Most research comes from wealthy countries, so we don't know what works best elsewhere. More research is needed to create simple, road-specific tools that help dispatchers send the right help, quickly and reliably, every time.

8.

In road traffic collisions, how do factors such as age, gender, and background influence the types of injuries people get and the care they receive, and what can be done to reduce inequalities?

Who you are can affect how you're treated after a road traffic collision. Older people often get hurt more easily, even in low-speed road traffic collisions, and women are more likely to be trapped in vehicles and have different types of injuries than men. People with lower income or education may also face delays in getting help. Current triage systems don't always account for these differences, which can lead to unfair care. To fix this, we need better tools that consider age, gender, and background when making emergency decisions, and safer vehicles designed with all types of people in mind.

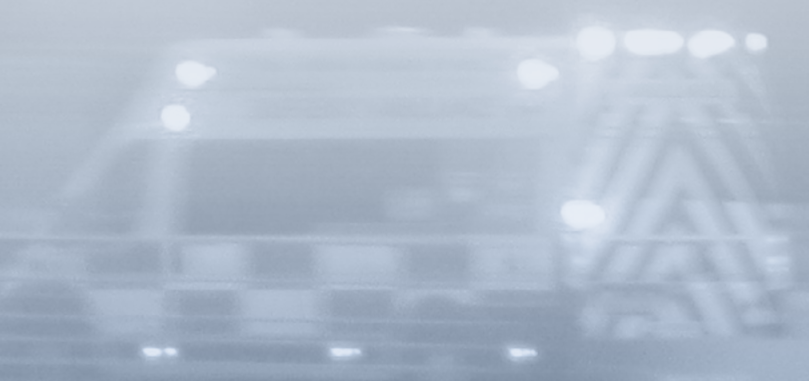

9.

Which urgent treatments work best for people trapped after a road traffic collision, and how can non-medical responders safely provide them?

Entrapped patients are at higher risk of severe injuries that require urgent treatment. There is, however, little high-quality evidence for which interventions are more effective in this group. Current practice relies on wider trauma evidence and expert consensus, focusing on early fluid resuscitation and medication to mitigate the consequences of muscle breakdown. Bystanders may have a role in initial management, but evidence is lacking. Further research is needed to identify the most effective interventions and improve survival in this group.

10.

What does a successful recovery after a road injury really mean, and are researchers measuring what matters most to patients?

There are many outcome measures for defining "successful outcomes" following trauma, but the outcome measures which exist are not universally applied in research. Further the commonly used outcome measures have not been developed in line with what patients consider to be successful outcomes which is largely around their subjective assessment and sense of self rather than the degree of functional disability. More work is needed to define a suitable outcome measure and align research with consistently applying it in studies.

SECTION 3

Emerging Themes and Further Reflections on the Top Ten

Reframing Vulnerability: Insights for Future Engagement

The evaluation included questions exploring both the possibility that participation may benefit the individual as well as have adverse effects. A small number of healthcare professionals indicated strongly that this workshop brought up difficult feelings for them, however, also indicated the value and meaning they found from connecting with others with similar experiences.

Measuring these aspects was intentional and recognises the importance of:

- Creating a safe space for participants to make participation possible
- Ensuring that a trauma informed approach was integrated into the design of the day.

Such findings indicate that despite the emotionally complex nature of the subject matter, participation can be rewarding and empowering, potentially providing benefits which go beyond the stated aims of the PSP. This invites a fundamental shift in how we might think about engagement within research, particularly how we see participation through the lens of 'vulnerability'.

Importantly for IMPACT, embedding this understanding within our research culture will ensure we are designing approaches that prioritise emotional safety, acknowledge complexity, and embrace the mutual benefits of shared experiences for both the public, clinicians and researchers.

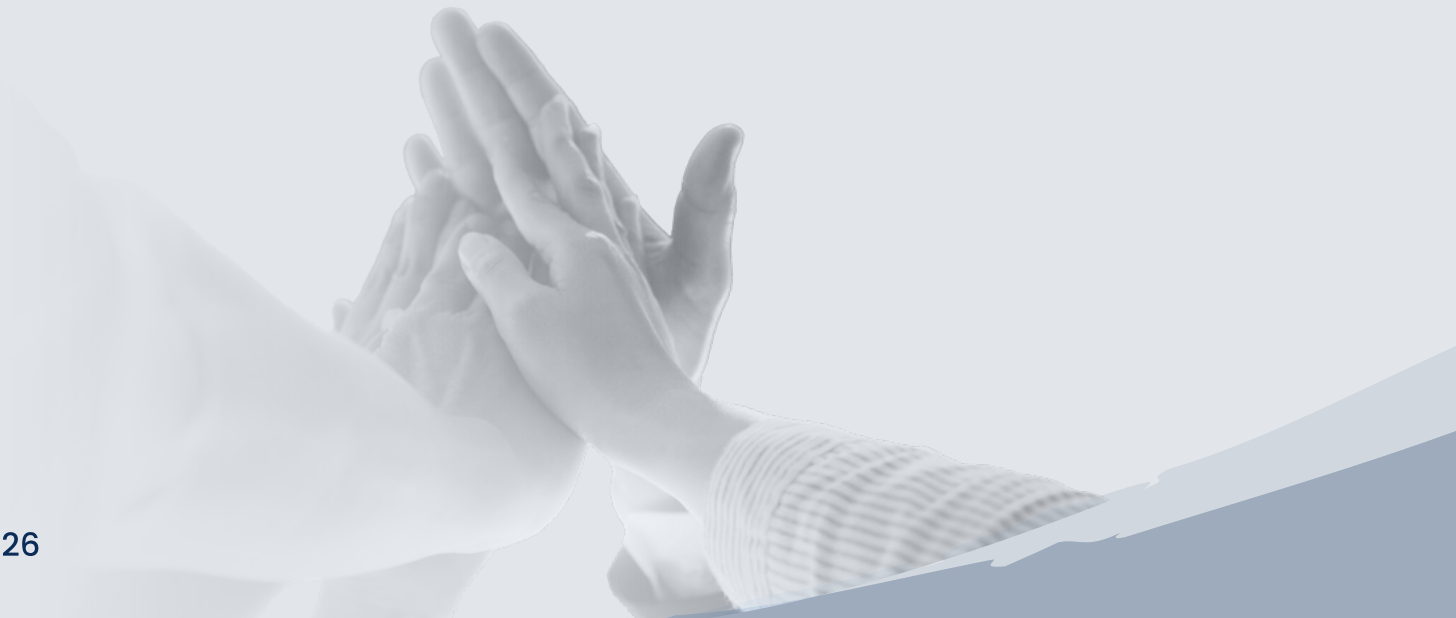

What do the Top Ten tell us?

The Top Ten collectively span the Road Injury Chain of Survival, however, they are most prominent in the first three links (Early Recognition, Early Rescue and Early Initial Care).

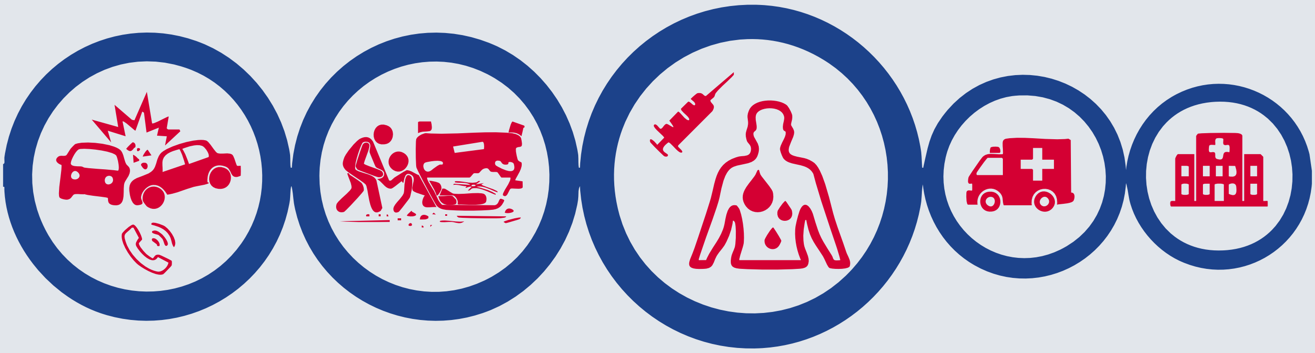

Distribution of Top Ten research priorities across the Road Injury Chain of Survival - each circle is proportional to the number of Top Ten priorities addressing that specific link.

There were three priorities that participants felt spanned across all the links in the survival chain:

- Understanding and reducing inequalities, specifically around age, sex, gender or background.
- Defining what 'successful recovery' looks like to the patient, reflecting real-life wellbeing rather than medical 'markers'.
- How information is joined up across the system.

Many of the top questions relate to death that might be prevented by acting quickly at key moments, and by spotting hidden but life-threatening injuries, at the scene. A second dominant theme relates to system-coordination and decision making, how emergency response services work together, how dispatch services can be supported to recognise serious injury and how vehicle technology and data might enable more accurate emergency response.

These priorities fit with national road safety strategic aims, specifically the use of technology to strengthen (not replace) human judgement in early care. They highlight the need to tackle inequalities and focus on long-term recovery, reminding us that early care should improve not just survival but also people's future health, independence and overall quality of life (as defined by the individual).

The importance of context in shaping priorities

Post-collision response and road injury is experienced and perceived differently, as well as there being inequalities in risk, recoveries and health outcomes. We went into this process asking the question:

> ***"Does sex, mode of travel, socioeconomic or living environment status of the survey participant, impact the nature of the research question submitted?"***

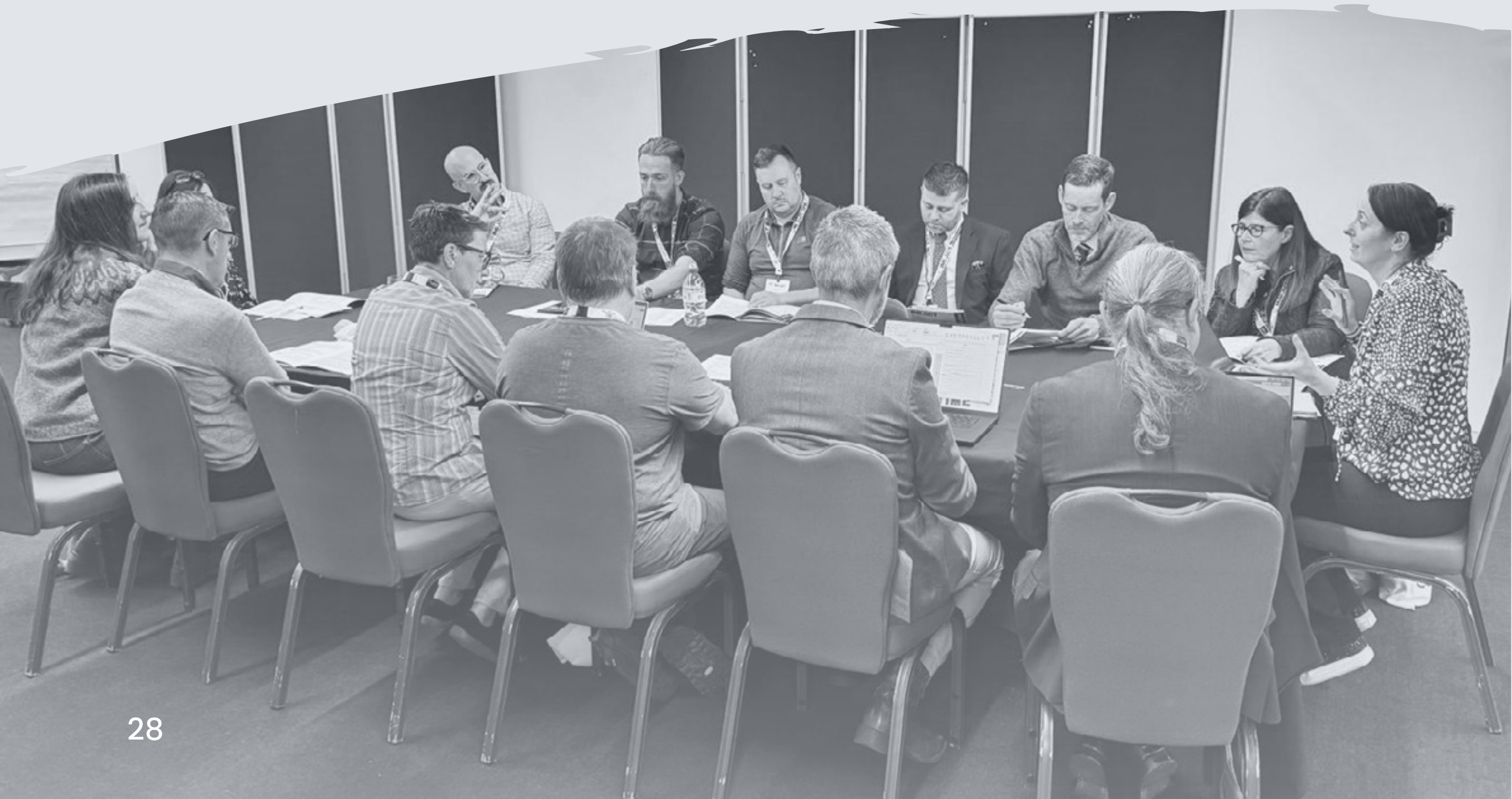

By linking ***who*** is speaking to ***what*** they prioritise throughout, we could use this process as a springboard to trying to understand some of the nuances in this area of research and inform an equitable, targeted and impact-orientated approach to the final research questions as well as our wider research programme.

We reviewed the questions submitted where demographic information had been provided, noting themes that came up.

We identified six themes:

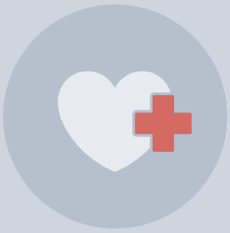

Clinical care and on-scene management:

This relates to immediate clinical response at the scene of a road traffic collision within the first few minutes/ hours, how responders make clinical decisions and what techniques, tools, procedures could influence patient outcomes.

Included:
- Immediate interventions
- Pain management
- Extrication
- TXA
- Airway
- Pain relief

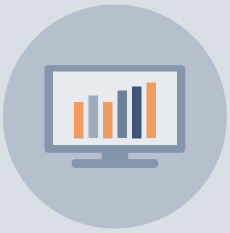

Technology and data:

The tools and data flows that enable recognition, decision-support, and information-sharing. There was an enthusiasm to use technology to improve many of the domains in the Road Injury Chain of Survival. It was future-focussed, looking at data integration, digital tools, tech enabled pathways.

Included:
- eCall
- Vehicle telemetry
- Data access
- Digital tools for faster responses
- Vehicle data
- Road safety

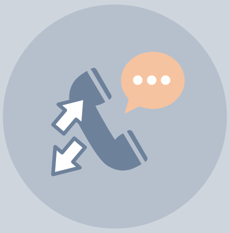

Communication and call triage:

This relates to the call-handling process, including the phrasing, terminology, questions and decision-making used by the call-handler when a road traffic collision is reported. It includes call-centre triage, dispatcher algorithms, phrasing of questions and how risk is assessed from a phone conversation. This was kept deliberately narrow to reflect that call triage is a specialist skill and a distinct step in the chain of survival. It also reflects how people felt that improvement in this area could have a cascading benefit for timelines of care and resource allocation.

Included:
- Call-centre scripts
- Dispatcher prompts
- Terminology consistency

Systems and coordination:

This focussed on overall functioning of the first responder system such as multi-agency working, pathway design, standards, resource deployment etc within ambulance, fire, police, hospital services. It included themes relating to national standards, variations in practice, handovers, learning and outcome frameworks. There was a difference in the overall objective of the question from respondents here, which seemed to speak to system reliability rather than individual clinical actions, highlighting gaps in consistency, communication between services and outcomes frameworks.

Included:

- Multi-agency processes
- Resource deployment
- Learning loops
- National standards
- System-wide
- Non-healthcare response
- Governance

Public/bystander readiness:

This theme included questions concerning the role of members of the public at the scene of a road traffic collision, including knowledge, confidence levels, training and what actions they should take/not take before clinical responders arrive. This theme provided insight into opportunities for public empowerment, such as education, national campaigns, or simple tools that could improve outcomes in the first few minutes before an ambulance arrives

Included:

- Training
- Public access interventions
- Empowerment strategies
- Public first aid

Recovery, equity and safety:

This captured concerns around rehab, follow-up, psychological support and inequities experienced across different communities. It combined both the recovery journey and structural inequities that might shape someone's experience of post-collision care. It picked up on how the nature of what happens in the post-collision space is important for longer term recovery and health outcomes.

Included:

- Rehabilitation
- Psychological support
- Sex disparities
- Postcode inequities
- Cultural barriers
- Vehicle/road safety
- Holistic care
- Holistic support
- Barrier

We were then able to explore these themes in relation to the sex, overall deprivation levels and environment deprivation levels in the area of the country the survey respondent was from. For this we used Indices of Multiple Deprivation (IMD) and Living Environment-IMD (LE-IMD) scores, and the legal definition of sex, see Glossary for definition.

We could also explore the depth of how these themes manifested, i.e "within this group, what themes dominate attention", and the breadth "across this group, how widely was this theme raised".

Understanding these nuances enables us to begin thinking about the 'what next' - how these patterns could inform IMPACT's approach to future research agenda, where translation efforts might need to be more targeted and concentrated, or responding more broadly to widely shared priorities.

What we found:

Below we outline the emerging themes and trends within the data. While several other themes emerged, they are not discussed in depth here as they did not show distinctive patterns or insights beyond the broader themes already described.

Clinical Care and On-Scene Management

This theme was seen as the ***highest priority across all demographic groups***. Regardless of sex, deprivation level, or transport mode, participants consistently emphasised the importance of spotting serious injuries early, making rapid and accurate decisions at the scene, and delivering effective first-hour care.

Communication and Triage

Communication & Call Triage was seen as a much higher priority in high-deprivation areas than across other demographic groups - where it was seen as the lowest priority.

This suggests that people in more deprived areas particularly value accurate early assessment during the emergency call, seeing this as a crucial point where serious injuries can be missed, or responses delayed.

Technology and Data

Participants from more deprived areas placed much greater emphasis on Technology and Data than those from lower-deprivation backgrounds. Low IMD and LE-IMD groups placed this as a much lower priority than High IMD and LE-IMD group, where the theme emerged as the top priority.

This could indicate a belief, particularly among people in more disadvantaged settings, that better information flow, connected systems, and vehicle/scene data could help overcome gaps in early recognition and response.

Recovery, Equity and Safety

When exploring the depth of this theme, it was highly prioritised by groups from areas of high-deprivation across overall IMD and LE-IMD, however, it was considered the lowest priority when adjusting for breadth across high-deprivation LE-IMD.

This could indicate a concentrated, equity-critical burden (high-impact issues) across certain groups which is not yet widely voiced across all respondents.

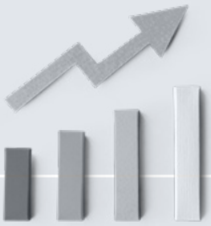

Priorities across groups

High deprivation

For participants in areas of higher overall deprivation, this theme came through strongly and was considered close to the top of their priority list. When we explored this by LE-IMD, the priority pivoted towards Technology & Data, highlighting how environmental disadvantage could possibly impact views on what will improve early response.

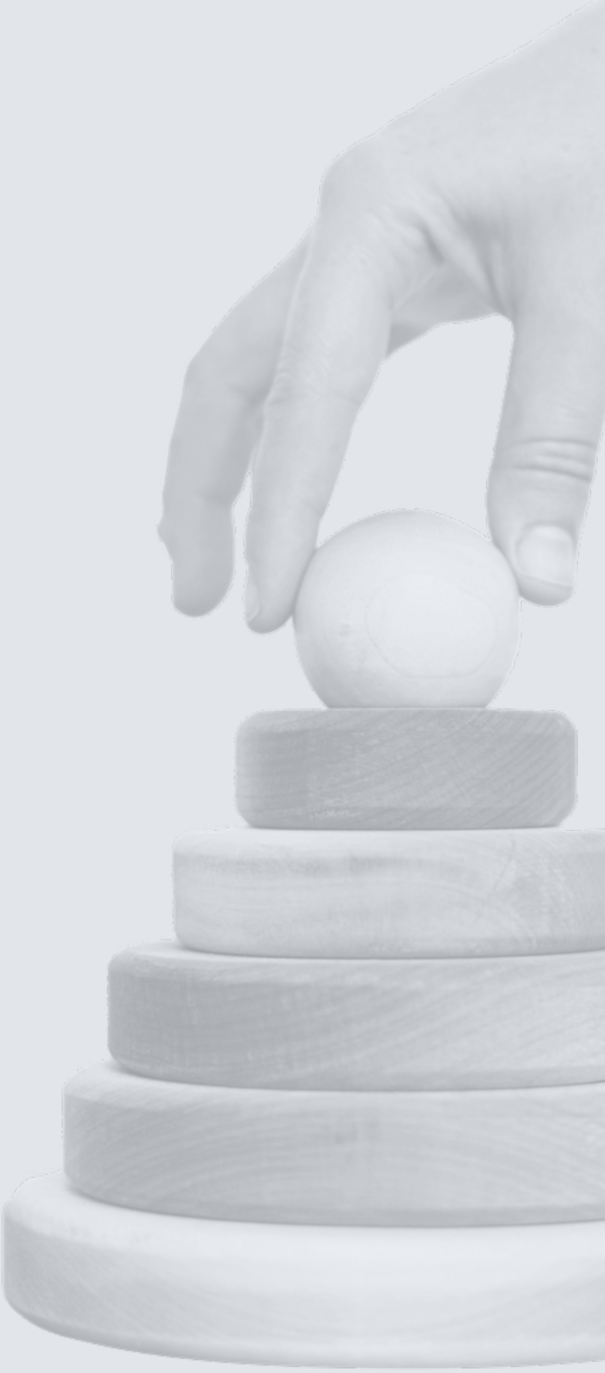

Low deprivation

Participants in lower-deprivation areas placed relatively more emphasis on Public/Bystander Readiness, and Systems & Coordination

This indicates this group is more likely to readily notice system-level inefficiencies (handover reliability, coordination, SOPs).

Sex differences

Women prioritised Technology & Data and Recovery, Equity & Safety, aligning with known differences in road traffic collision biomechanics, injury patterns, and barriers to rehabilitation or symptom validation.

Men prioritised Clinical Care & Scene Management and Bystander Readiness, reflecting a focus on the visible, immediate aspects of emergency response.

Primary transport mode

Among drivers, priorities centred on what happens at the scene and how the system works end-to-end, with substantial attention to recovery, equity, and technology as enablers of better decisions and smoother care pathways.

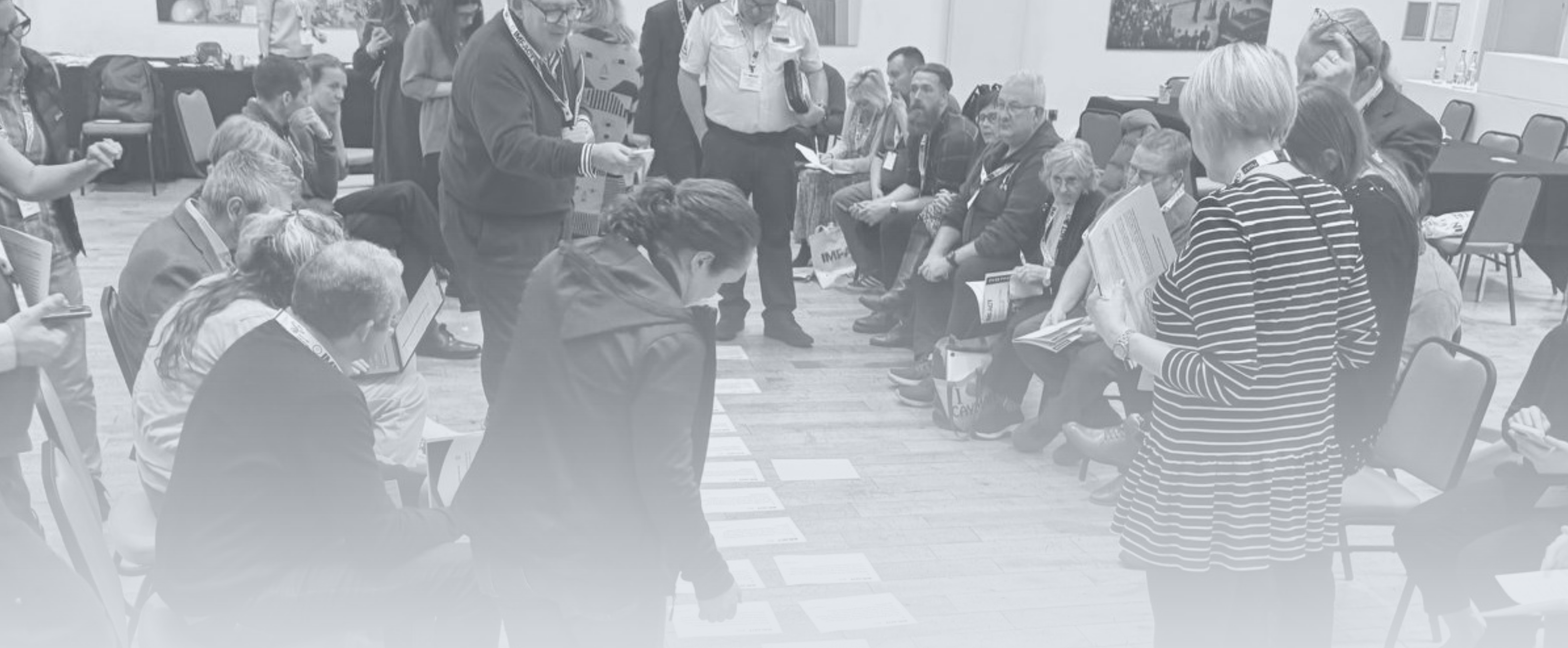

Summary

In summary, the Top Ten's early-link focus is not accidental. It reflects the consistent pattern seen at the question-collection stage from both men and women, from drivers, and across broad IMD groups: a clear priority to improve what happens first after a road traffic collision.

Alongside this, women's submissions tended to provide additional depth to the three cross-cutting priorities, particularly around inequalities, recovery, and information gaps. From a translational perspective, this suggests strong value in co-designing research and interventions with women and in prioritising advances in technology and data, both of which may strengthen implementation and increase impact.

Equity-related themes also showed high depth within the groups most likely to experience disadvantage. The decision at the workshop to treat inequalities as a cross-cutting theme is therefore well-founded and aligns with principles widely recognised across health research: some issues are vital not because they are raised by the majority, but because they matter most to those at greatest risk. Inequalities are, by their nature, unlikely to be raised by majority groups, but the degree with which they were expressed by disadvantaged groups in this PSP gives them clear translational importance.

System coordination, while valued across all submissions, was most visible where capacity and reliability are already stronger. People who experience more predictable services appear more likely to notice issues such as handover reliability or inconsistencies in multi-agency working. For translation, this suggests a staged approach: piloting system-coordination improvements in lower-deprivation areas where adoption readiness is higher, then adapting and tailoring these approaches for higher-deprivation settings to ensure equitable benefit across communities.

> **"It was incredible to have a group of people with such varied experience and expertise in the room. I learnt a huge amount…I'm excited to see what happens next!"**
> **~ Healthcare professional**

What happens now?

The identification of a Top Ten list of priorities is not the end of the journey, it is the beginning of a critical phase where impact is realised. To ensure that the priorities influence research, policy, and practice,

1. **Strengthen public and lived-experience involvement**
   People with lived experience were central to shaping these priorities and will continue to play a key role going forward; the following goals will ensure that the priorities remain grounded in real-world experience and continue to reflect the needs of those affected:

   - **Maintain** involvement in interpretation and translation of the priorities into researchable questions.
   - **Create** opportunities for co-production, ensuring lived experience shape new studies, proposals and dissemination.
   - **Support** advisory and oversight roles, enabling people with lived experience to guide decision-making as research develops.
   - **Embed** meaningful involvement in evaluation, ensuring ongoing relevance and authenticity as evidence progresses.

2. **Enable and influence funding**
   Maximising research impact requires strong engagement with funders and commissioning services. The following goals aim to build shared understanding of need and encourage funders to invest in evidence that will strengthen outcomes across road safety and post-collision care:

Work with research funders, charitable organisations and commissioning bodies to highlight the importance of these priorities.

Support researchers to align proposals with these priorities, strengthening coherence across the sector.

Provide evidence and context to support themed funding calls and targeted investment.

Facilitate visibility of emerging projects so funders can clearly identify how new studies respond to the PSP.

3. **Integrate Priorities into National and Local Strategies**
   This work focuses on influencing policy, strategy and system design, ensuring research questions are embedded within wider safety and health agendas. To ensure the priorities have this system-level influence:

   - Share the findings with national, regional and local organisations responsible for road safety, trauma, emergency care and health planning.
   - Support alignment with existing frameworks, including those based on the Road Injury Chain of Survival.
   - Promote consideration of these priorities in policy development, commissioning decisions and improvement programmes.
   - Work with system-leaders to embed the priorities within strategic guidance and long-term planning.

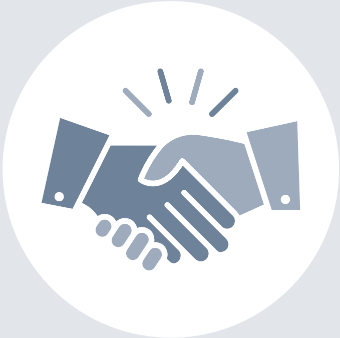

4. **Promote Transparency and Open Access**
   Transparency will enable broader uptake, reduce duplication and encourage coordinated action across the sector:

   - All PSP outputs, including the summary report, methodology, and final priorities, are made publicly available.
   - Resources are shared through open-access platforms wherever possible.
   - Clear signposting and targeted dissemination to support the application of findings across different audiences and settings.

5. **Deliver a Coordinated Communication and Dissemination Plan**
   To sustain momentum, we will adopt a collaborative approach to ensure persistent awareness and ongoing engagement:

Continue developing a strategic communications plan that ensures outputs and developments reach the right audiences at the right time.

Use multiple channels such as peer-reviewed publications, social media, events, professional forums, and lived-experience networks to share progress.

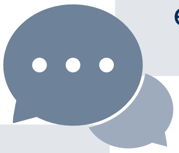

Work with partners (including patient groups, professional bodies, charities, and research networks) to amplify the priorities.

Highlight examples of emerging work responding to the priorities, encouraging knowledge sharing and follow-through.

6. **Support Translation of Priorities into Research and Practice**
   To mobilise these questions into funded studies, the following aims will support the development of high-quality research:

   - Facilitate workshops and researcher–stakeholder meetings to help translate broad priorities into specific, fundable research questions.
   - Encourage cross-sector partnerships, bringing together road safety experts, healthcare providers, academics, lived-experience contributors, and policymakers.
   - Promote multisite and interdisciplinary approaches, recognising that many priorities cross organisational and sector boundaries.

We will continue to encourage our partners to share progress, success stories and real-world impact case studies to create a collective picture of advancement.

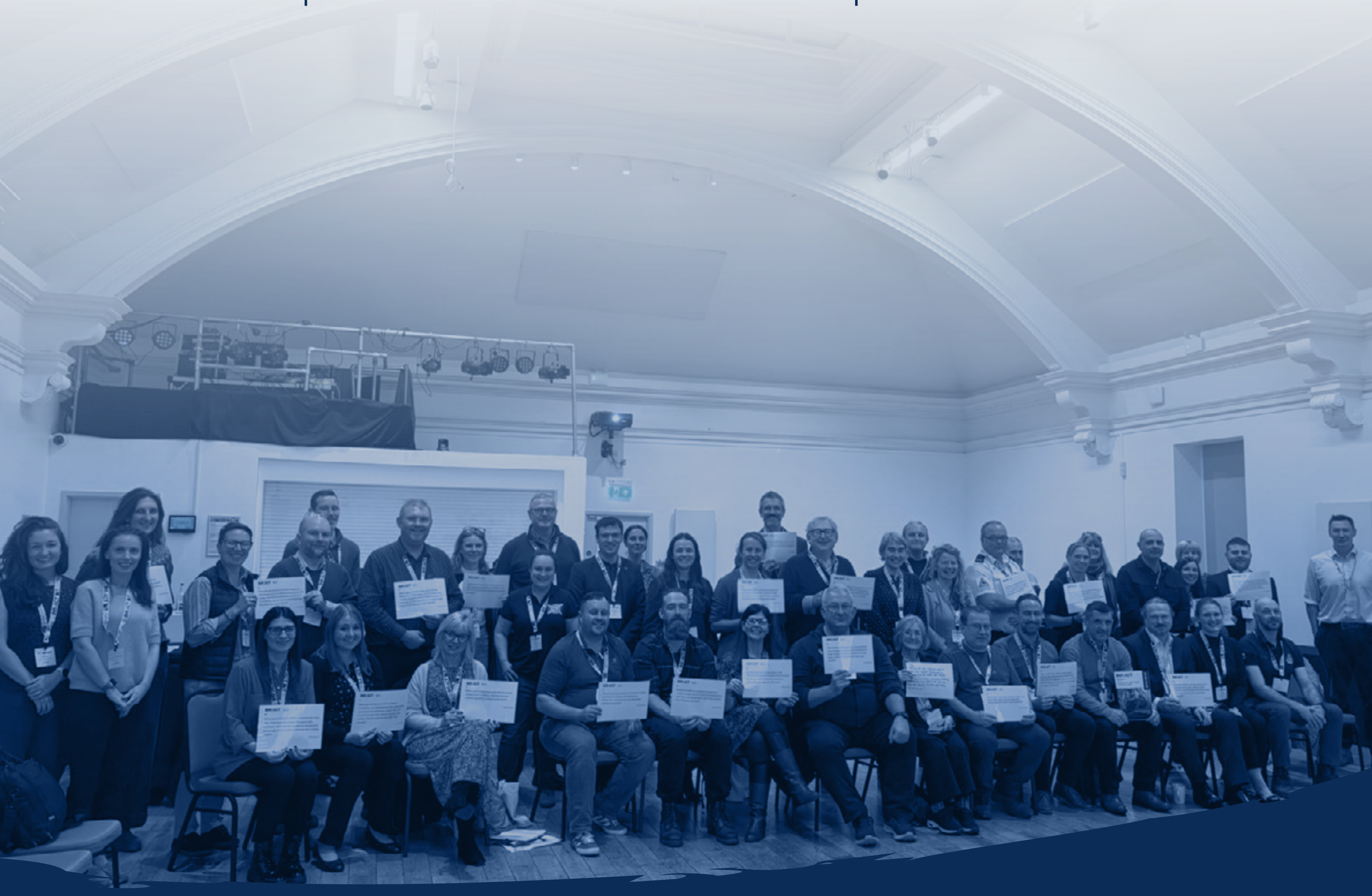

GLOSSARY

Index of Multiple Deprivation (IMD)
The Index of Multiple Deprivation is a way of measuring how disadvantaged different neighbourhoods are. It combines information about income, employment, education, health, crime, housing and access to services to give each area a score. Think of this as context about where people live, not a definitive measure of any individual.

Lower IMD numbers (1-4) mean an area is more deprived, while higher numbers (5–10) mean less deprived.

Index of Multiple Deprivation – Living Environment (IMD-LE)
The Living Environment part of IMD looks specifically at how much the physical conditions where people live might affect their health. We've explored this as it contains data around road safety related to pedestrians and cyclists collisions.

Socio-economic Status (SES)
Socio-economic status describes someone's ***social and economic position***. It can include income, education, job type, housing and other life circumstances.

Higher SES usually means more financial or social resources; lower SES means fewer. In this project, SES was not directly measured, but IMD and IMD-LE act as proxies to help identify patterns linked to advantage or disadvantage.

Demographic
A demographic is a characteristic used to describe groups of people.

Examples include age, sex, deprivation level, ethnicity, or primary mode of transport. Looking at demographics helps us understand whether certain groups have different experiences, needs, or priorities.

Median Score
The median is a type of average.

If you line up all the scores from lowest to highest, the median is the one in the middle. It is useful because it isn't skewed by very high or very low scores, making it a good way to understand what most people think or prefer.

Definition of sex used in this report
In this project, "sex" was used based on the legal/biological definition (male/female). This choice was made because:

- The project included an inequalities lens (sex-linked differences in access or outcomes), and
- Biomechanics play a role in road traffic collisions (men and women can experience different injury patterns, forces and recovery needs).

Using this definition helped us explore whether sex-based differences influenced the questions people asked, the needs they expressed, or the priorities they identified.

ACKNOWLEDGMENTS

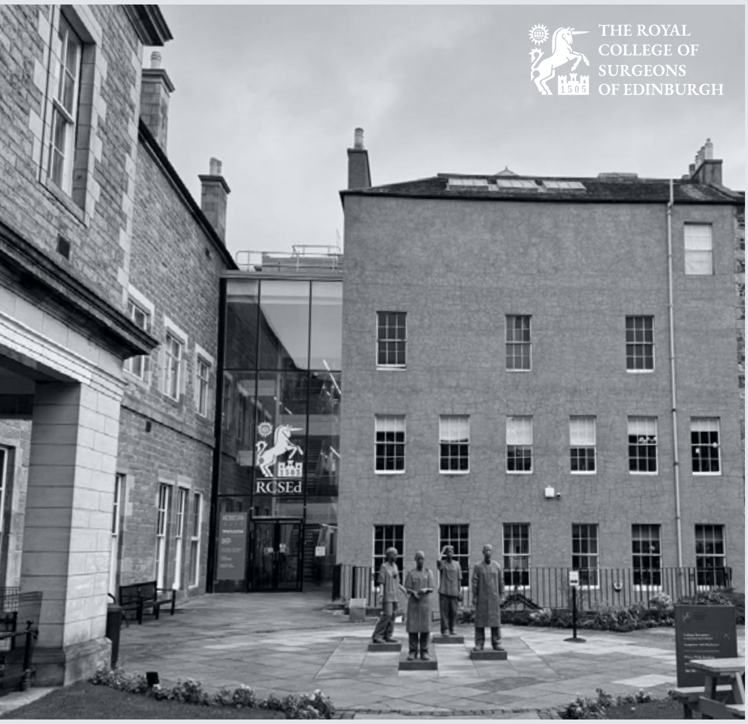

Project Partners -
Royal College of Surgeons,
Faculty of Pre-Hospital Care
Dr Pam Hardy
Gillian Mitchell and the
Faculty Development Team

Steering Group Members
We would like to acknowledge and sincerely thank the Steering Group for their time, effort and sharing their expertise and experience.

Stakeholder Representatives
Dr Elizabeth Box -
Royal Automobile Club (RAC)

Dr Claire Baker -
Imperial College London

Ian Dunbar OBE -
Fire and Rescue

Ian Maritt -
Humberside Fire and Rescue & United Kingdom Rescue Organisation

Ianto Guy -
Transport Research Laboratory (Collision)

Patient and Public Representatives
Brian Lee
Marilyn MacQueen

Healthcare Representatives
Professor Ed Barnard
Rob Fenwick
Dr Louise Johnson
Nigel Lang
Dr Caroline Leech
Professor Tim Nutbeam

Workshop Facilitation

Chair
Dr Laura Cottey

PSP Workshop Facilitators
Kerry Dungay
Dr Emily Foote
Dr Lauren Rodgers

Pastoral Support
Charlie Bowyer - Psychotherapeutic Counsellor at Eventide Counselling

Literature Review Authors
Dr Nick Aveyard
Dr Lily Eckersley-Jones
Cally Greiner-Cooper
Dr Celia Lugt
Kershun Mathew
Dr Kirsten Raphael
Dr George Russam
Dr Gregory Smith
Dr Luke Tester
Dr Jennifer Todd
Dr Rosanna Watts

Workshop Participants
Patients and Public
Nicola Brown
Lisa Challinor
Ellie Challinor-Hughes
Melanie Elmer
Sharron Huddleston
Ann Ralli

PROFESSIONALS

Name	Organisation	Role
Claire Baker	Imperial College London	Research Fellow
Ed Barnard	UK Defence (Research & Clinical Innovation) East Anglian Air Ambulance	Defence Professor of Emergency Medicine
Charlotte Bowyer	Eventide Counselling	Psychotherapeutic Counsellor
Elizabeth Box	RAC Foundation	Research Director
Jennifer Buchanan	Fieldfisher	Partner
Stephen Cash	Forensic Collision Investigation & Reconstruction Ltd	Principal Consultant
Ross Chalmers	Police Scotland	Roads policing officer
Andrea Connolly	NHS Lothian	Major trauma mental health nurse
Laura Cottey	Academic Department of Military Emergency Medicine	Emergency Medicine Registrar
Naomi Dodds	Faculty of Pre-Hospital Care (FPHC), Royal College of Surgeons of Edinburgh	FPHC North of Scotland Representative
Ian Dunbar (OBE)	Ian Dunbar Training and Consultancy	Director
Kerry Dungay	IMPACT - The Centre for Post-Collision Research, Innovation and Translation	Project Coordinator
Rob Fenwick	Betsi Cadwaladr University Health Board	Consultant Nurse
Emily Foote	Devon Air Ambulance/ NHS	Emergency Medicine doctor
Anna Forbes	Essex and Herts Air Ambulance /PHOTON	Flight Doctor
Els Freshwater	Scottish Ambulance Service	Advanced Practice Critical Care Clinical Lead
Iain Gibson	Scottish Ambulance Service	Paramedic
Shelley Gill	Brake, the road safety charity	Regional manager, Scotland
Ianto Guy	Transport Research Laboratory (TRL)	Principal Consultant - Collision Investigation

Pamela J Hardy	Faculty of Pre-hospital Care	Chair
Tom James	The RAF	Wing Commander and Medical Doctor
Saul Jeavons	Association for Road Risk Management / The Transafe Network	Chair of ARRM / Director of Transafe Network
Danny Kerr	Scottish Ambulance Service	Clinical Effectiveness Lead: Major Trauma
Ian Maritt	United Kingdom Rescue Organisation	Director of Education and Development
Alison Moggach	Scottish Ambulance Service	Advanced Nurse Practitioner
Gary Morgan	BASICS	Chief Officer
Jane Ogilvie	Scottish Fire and Rescue	Watch Commander- Training
Alastair Richards	Project PACKINGTON MoD	Paramedic Military Vehicle Specialist
Calum Reid	The Royal College of Surgeons Edinburgh	Faculty Development Manager
Lauren Rodgers	IMPACT - The Centre for Post-Collision Research, Innovation and Translation	Senior Research Coordinator
Connor Russell	Scottish Ambulance Service	Technician
Zoe Smeed	BASICS Scotland	Senior Medical Advisor
Andrew Smith	Faculty of Pre Hospital Care, Royal College of Surgeons of Edinburgh	Honorary Secretary, Chair Elect
Carl Smith	College of Paramedics	Head of Clinical Development Emergency and Critical Care
Matt Staton	Agilysis	Head of Consultancy
Paul Steinberg	The Road Safety Trust	Director of Communications and Engagement
Richard Stevenson	Police Scotland	Clinical Governance Advisor
Paul J Trafford	Motorsport UK	Chief Medical Officer
Margaret Winchcomb	Parliamentary Advisory Council for Transport Safety (PACTS)	Deputy Executive Director

FUNDING

This PSP is jointly funded by Transport Scotland and Vision Zero South West.
Transport Scotland's contribution supports the printing and publication of materials, open access fees for publications, and dissemination activities. Vision Zero South West provides funding for project coordination, survey administration, and organisation of the final prioritisation workshop. The funders had no role in the design, conduct, analysis, interpretation, or prioritisation decisions, which remain entirely under the authority of the Steering Group.

CONFLICT OF INTEREST STATEMENT:

All members of the PSP Steering Group and project team were asked to declare any personal, professional, or financial interests that could influence their involvement. No conflicts were identified that were considered to affect the integrity or independence of the process.

REFERENCES

1. World Health Organization. Global status report on road safety 2023. Report No: Global Geneva: World Health Organization [Internet]. 2023; https://www.who.int/publications/i/item/9789240086517

2. Reported road casualties in Great Britain, provisional estimates: 2024 - GOV.UK [Internet]. [cited 2025 Nov 16]. https://www.gov.uk/government/statistics/reported-road-casualties-great-britain-provisional-results-2024/reported-road-casualties-in-great-britain-provisional-estimates-2024. Accessed 16 Nov 2025

3. Department for Transport. Road Safety Strategy [Internet]. London; 2026. https://assets.publishing.service.gov.uk/media/695e2cff8832ab3a48513809/road-safety-strategy.pdf

4. Cuthbertson J, Drummond G. Prehospital Care Post-Road-Crash: A Systematic Review of the Literature. Prehosp Disaster med. 2025;40:94–100. https://doi.org/10.1017/S1049023X25000202

5. Nutbeam T, Stassen W. The road injury chain of survival: A framework for improving trauma outcomes. Injury. 2025;112285. https://doi.org/10.1016/j.injury.2025.112285

6. JLA Guidebook | NIHR JLA [Internet]. [cited 2025 Aug 20]. https://www.jla.nihr.ac.uk/jla-guidebook. Accessed 20 Aug 2025

7. Nutbeam T, Lee B, Johnson L, Leech C, Baker CE, Barnard EB, et al. Identifying Research Priorities for Post-Collision Care in the United Kingdom: Protocol for a Road Injury Priority Setting Partnership [Internet]. OSF Registries; 2025 [cited 2026 Jan 27]. https://doi.org/10.17605/OSF.IO/NB9YM

8. Tong A, Synnot A, Crowe S, Hill S, Matus A, Scholes-Robertson N, et al. Reporting guideline for priority setting of health research (REPRISE). BMC Med Res Methodol. 2019;19:243. https://doi.org/10.1186/s12874-019-0889-3

9. Khan MN, Das S. Advancing traffic safety through the safe system approach: A systematic review. Accident Analysis & Prevention. 2024;199:107518. https://doi.org/10.1016/j.aap.2024.107518

10. Mackway-Jones K. bestbets.org: Odds on favourite for evidence in emergency medicine reaches the world wide web. Emergency Medicine Journal. 2000;17:235-a-236. https://doi.org/10.1136/emj.17.4.235-a

11. Bretherton CP, Hirst R, Gacaferi H, Gower J, Exell L, Johnston S, et al. Research priorities for the management of major trauma: an international priority setting partnership with the James Lind Alliance. BMJ Open. 2024;14:e083450. https://doi.org/10.1136/bmjopen-2023-083450

12. Smith J, Keating L, Flowerdew L, O'Brien R, McIntyre S, Morley R, et al. An Emergency Medicine Research Priority Setting Partnership to establish the top 10 research priorities in emergency medicine. Emerg Med J. 2017;34:454–6. https://doi.org/10.1136/emermed-2017-206702

13. Abdullah N, Nutbeam T, Saunders CJ, Wylie C, Lang N, Stassen W. Consensus-based research priorities for post-collision care in the Western Cape province of South Africa. African Journal of Emergency Medicine. 2025;15:100900. https://doi.org/10.1016/j.afjem.2025.100900

14. Goolsby C, Bosson N, Banjo E, Dacuyan-Faucher N, Schlesinger S, Whitfield D, et al. National research agenda for postcrash care. J Trauma Acute Care Surg. 2025;98:942–50. https://doi.org/10.1097/TA.0000000000004589

15. World report on road traffic injury prevention [Internet]. [cited 2025 Nov 16]. https://www.who.int/publications/i/item/9241562609. Accessed 16 Nov 2025

16. Harmsen AMK, Giannakopoulos GF, Moerbeek PR, Jansma EP, Bonjer HJ, Bloemers FW. The influence of prehospital time on trauma patients outcome: a systematic review. Injury. 2015;46:602–9. https://doi.org/10.1016/j.injury.2015.01.008

CONTACT INFORMATION

Postal address:
IMPACT - Centre for Post-Collision Research,
Innovation and Translation,
Devon Air Ambulance,
5 Sandpiper Court,
Harrington Lane,
Exeter
EX4 8NS.

If you should have any enquiries or feedback please contact:
hello@post-collision.com

For more information on IMPACT and the work we do, please visit our website:
www.post-collision.com

SUGGESTED CITATION:

Road Injury Priority Setting Partnership. Report on Post Collision Research Priorities 2025. United Kingdom: Devon Air Ambulance, IMPACT - Centre for Post-Collison Research, Translation and Innovation; 2026. DOI (10.1186/s13049-027-01589-7)
